# Design, Synthesis, and Evaluation of New 1*H*-Benzo[*d*]imidazole Based PqsR Inhibitors as Adjuvant Therapy for *Pseudomonas aeruginosa* Infections

**DOI:** 10.1021/acs.jmedchem.3c00973

**Published:** 2024-01-03

**Authors:** Fadi Soukarieh, Alaa Mashabi, William Richardson, Eduard Vico Oton, Manuel Romero, Jean-Frédéric Dubern, Shaun N. Robertson, Simone Lucanto, Zoe Markham-Lee, Tomás Sou, Irena Kukavica-Ibrulj, Roger C. Levesque, Christel A. S. Bergstrom, Nigel Halliday, Barrie Kellam, Jonas Emsley, Stephan Heeb, Paul Williams, Michael J. Stocks, Miguel Cámara

**Affiliations:** † School of Life Sciences, University of Nottingham Biodiscovery Institute, University of Nottingham, Nottingham NG7 2RD, U.K.; ‡ School of Pharmacy, University of Nottingham Biodiscovery Institute, University of Nottingham, Nottingham NG7 2RD, U.K.; § The National Biofilms Innovation Centre, University of Nottingham Biodiscovery Institute, University of Nottingham, Nottingham NG7 2RD, U.K.; ∥ Department of Pharmacy, Uppsala University, Uppsala SE-751 23, Sweden; ⊥ Institut de Biologie Intégrative et des SystèmesUniversité Laval, Quebec G1V 0A6, Canada

## Abstract

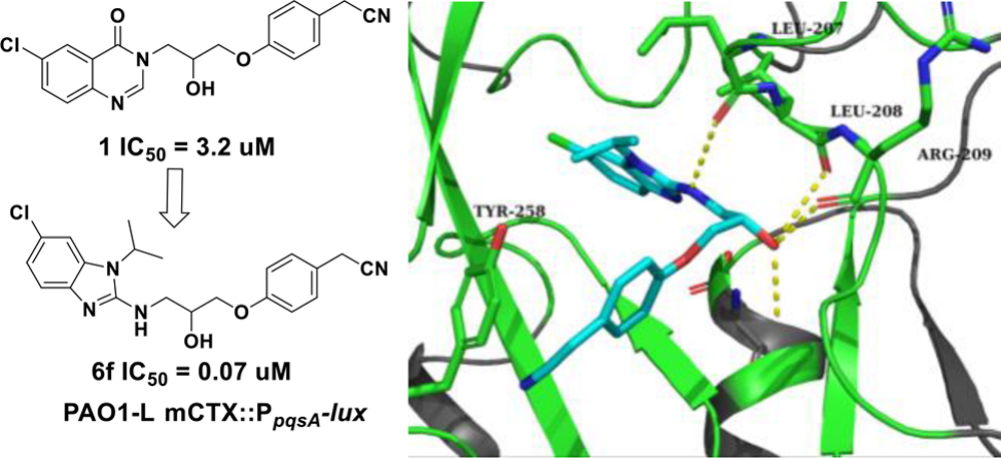

*Pseudomonas aeruginosa* is one of the top priority pathogens that requires immediate attention according to the World Health Organisation (WHO). Due to the alarming shortage of novel antimicrobials, targeting quorum sensing (QS), a bacterial cell to cell signaling system controlling virulence, has emerged as a promising approach as an antibiotic adjuvant therapy. Interference with the *pqs* system, one of three QS systems in *P. aeruginosa*, results in reduction of bacterial virulence gene expression and biofilm maturation. Herein, we report a hit to lead process to fine-tune the potency of our previously reported inhibitor **1** (IC_50_ 3.2 μM in *P. aeruginosa* PAO1-L), which led to the discovery of 2-(4-(3-((6-chloro-1-isopropyl-1*H*-benzo[*d*]imidazol-2-yl)amino)-2-hydroxypropoxy)phenyl)acetonitrile (**6f**) as a potent PqsR antagonist. Compound **6f** inhibited the PqsR-controlled P_*pqsA*_-*lux* transcriptional reporter fusion in *P. aeruginosa* at low submicromolar concentrations. Moreover, **6f** showed improved efficacy against *P. aeruginosa* CF isolates with significant inhibition of pyocyanin, 2-alkyl-4(1*H*)-quinolones production.

## Introduction

Inhibition of quorum sensing (QS), a cell-to-cell signaling mechanism used by bacterial populations to control the production of virulence traits and antibiotic resistance mechanisms, has attracted the attention of antibacterial drug discovery research over the past two decades.^1,2^ Unlike antibiotics, the concept behind this approach relies on combatting bacterial virulence without affecting the viability of the organism and hence inducing milder selective pressure, which may lead to a lower rate of resistance development.^3^ Due to the central role of QS systems in the control of virulence gene expression within bacterial populations, pharmacological inhibition of these systems provides a promising strategy as an antibiotic-adjuvant to reduce bacterial virulence.^4,5^*Pseudomonasaeruginosa* (PA) is a Gram-negative opportunistic bacterium and a common cause of nosocomial infections particularly in cystic fibrosis (CF) and immunocompromised patients.^6^ PA possesses three QS systems known as *las, rhl*, and the Pseudomonas Quinolone System (*pqs*).^7^ These systems produce signal molecules, known as autoinducers (AIs), which upon reaching a certain threshold concentration at a high population density, activate their corresponding receptor proteins and form complexes which in turn induce the transcription of AI biosynthetic genes as well as those that code for numerous virulence factors.^3^ The chemical classes of AIs are structurally diverse where the *las* and *rhl* systems use *N*-acylated-l-homoserine lactone derivatives, while the *pqs* system relies on 2-alkyl-4(1*H*)-quinolone derived compounds (AQs).^7^ The biosynthesis of AQs including the signal molecules 2-heptyl-3-hydroxy-4(1*H*)-quinolone (PQS) and 2-heptyl-4-hydroxyquinoline (HHQ) depends on a group of enzymes (PqsA, PqsBC, PqsD, PqsE, PqsH) and the starting substrates, anthraniloyl-CoA and malonyl-CoA.^8^ Both PQS, and HHQ bind to the LysR type regulator PqsR (also called MvfR) inducing conformational changes and leading to a positive feedback loop through the transcriptional activation of the *pqsABCDE* operon.^9,10^ PqsR was therefore identified as a critical element for a fully functional *pqs* system. In fact, *pqsR* deletion mutants fail to produce *pqs*-controlled virulence genes such as elastase and pyocyanin.^11^ More importantly, the *pqs* system regulates biofilm maturation, a highly antibiotic tolerant lifestyle for maintaining bacterial populations in low nutrient environments and chronic infections.^12^ In this study, we report a hit to lead study on our previously reported PqsR inhibitor **1**(13) following a structure–activity relationship approach (SAR), which led to the discovery of new 1*H*-benzo[*d*]imidazole series of PqsR antagonists. Compound **6f** was evaluated for its effect on PA phenotypes including pyocyanin and AQ signal levels in various laboratory strains and CF isolates. The effect of **6f** on PA biofilms was also investigated to establish whether it could enhance the action of antibiotics such as ciprofloxacin or tobramycin. Finally, to gain further understanding on its suitability for further development, compound **6f** was assessed for its cytotoxicity in an A549 adenocarcinoma human alveolar basal epithelial cell line.

## Results

### Rational Design and Hit Exploration

**Figure 1:**
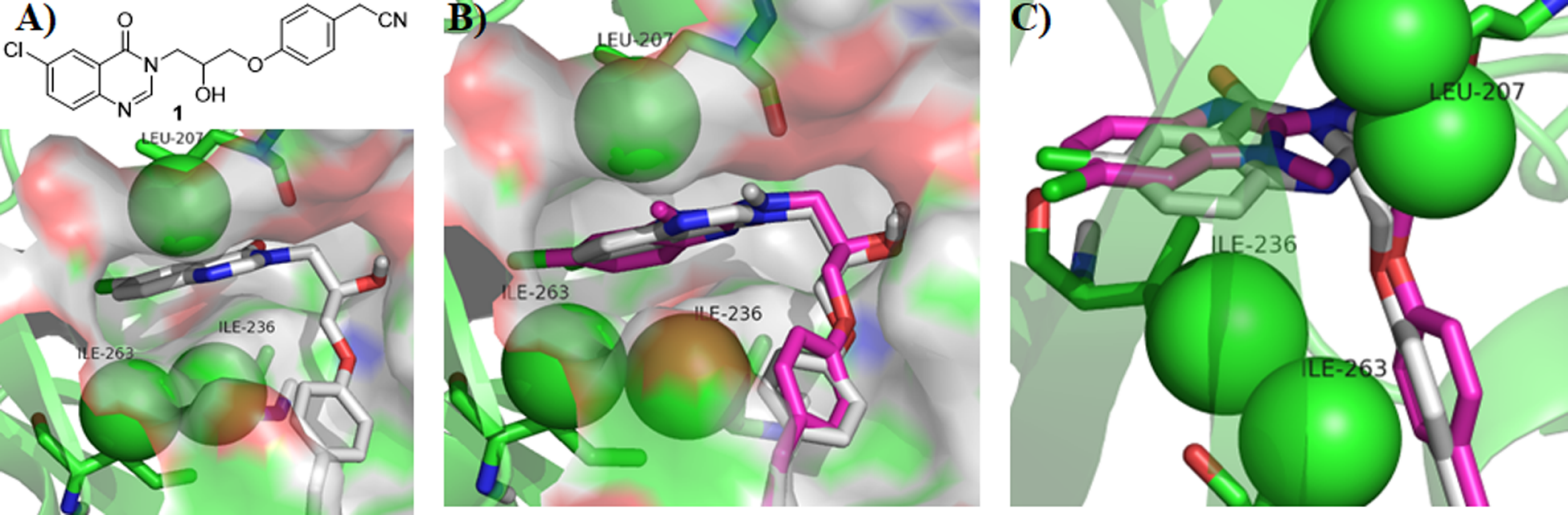
Rational design of benzo[*d*]imidazole PqsR antagonists: (A) Chemical structure of compound **1** and schematic representation of its crystal structure complexed in the PqsR^LBD^ (white sticks), green spheres represent potential lipophilic residues, PDB 7O2T. (B,C) Overlay of compounds **1** (white sticks) and **6a** (magenta sticks) poses in the PqsR^LBD^ generated using Schrödinger Suite for molecular docking (PDB 7O2T).

We previously reported the discovery and SAR of the quinazolin-4(3*H*)-one scaffold as *P. aeruginosa* PqsR antagonists, and we concluded that **1** (Table 1) was one of the most potent PqsR inhibitors from within this series.^13^ The quinazolinone series showed limited improvement of potency despite the concerted effort to diversify the SAR. The reported crystal structure in our previous study showed that the PqsR pocket is not fully occupied by this ligand and there is potential hydrophobic interactions that can be gained with Leu^207^, Ile^236^, and Ile^263^ that could enhance the potency of this inhibitor (Figure1). Therefore, alternative heterocyclic systems for the quinazolinone headgroup of **1** were considered and evaluated using molecular modeling approach (Figure 1). Interestingly, substituted [6,5] ring system, such as benzimidazole, benzothiazole, and benzoxazole heterocycles, showed promising docking results in the PqsR ligand binding domain.

**Table 1:**
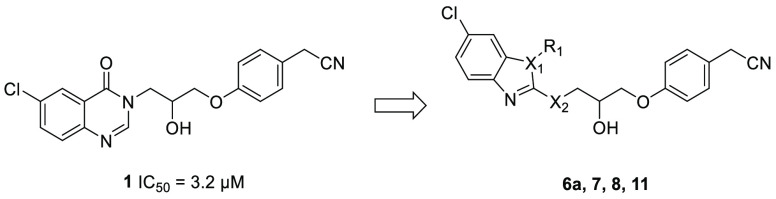
Activity of Analogues of Compound **1** with Various Heterocyclic System Replacements

compd	X_1_	X_2_	R_1_	IC_50_ (μM)^a^ PAO1-L P_*pqsA*_-*lux*	IC_50_ (μM)^a^ PA14 P_*pqsA*_-lux
**6a**	N	NH	CH_3_	0.21 ± 0.04	0.20 ± 0.02
**7**	O	NH		NA	NA
**8**	S	NH		NA	NA
**11**	N	S	CH_3_	NA	NA

a NA refers to no activity at 10 μM. IC_50_s were reported in PAO1-L P_*pqsA*_-lux and PA14 P_*pqsA*_-lux laboratory strains of *P. aeruginosa.* Values reported as mean ± SD of *n* = 2.

**Scheme 1:**
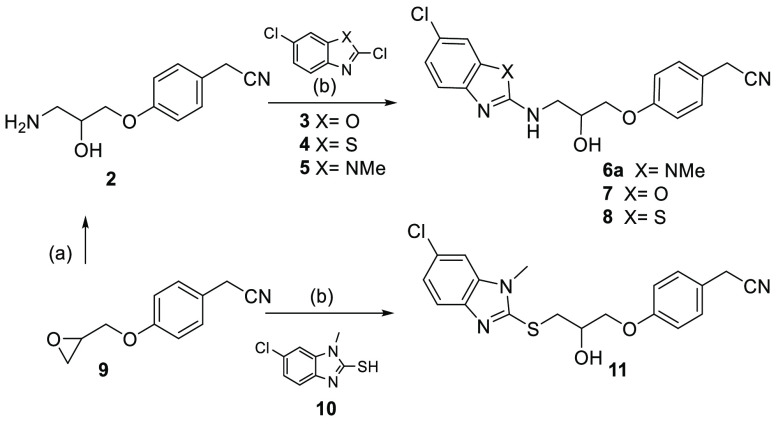
Synthetic Route for the Preparation of Analogues (**6a**, **7**, **8**, and **11**) Reagents and conditions: (a) NH_3_/MeOH, rt, 16 h, 90%; (b) microwave irradiation, Et_3_N, EtOH, 180 °C, 3 h, 10–25%.

Analogues of **1** containing the benzo[*d*]imidazole-2-amine **6a**, benzo[*d*]oxazol-2-amine **7**, and benzo[*d*]thiazol-2-amine **8** and head groups were prepared by substitution of the corresponding 2,6-dichlorobenzo[*d*]oxazole **3**, 2,6-dichlorobenzo[*d*] thiazole **4** and 2,6-dichlorobenzo[*d*]imidazole **5** with 2-(4-(3-amino-2-hydroxypropoxy) phenyl) acetonitrile **2**, which in turn was prepared from epoxide ring opening of 2-(4-(oxiran-2-ylmethoxy) phenyl) acetonitrile **9** with ammonia. While compound **11** was directly obtained following epoxide ring opening of **9** with 6-chloro-1-methyl-1*H*-benzo[*d*]imidazole-2-thiol **10** in ethanol under microwave irradiation at 180 °C (Scheme 1).^14^

Replacement with 1-methyl-1*H*-benzo[*d*]imidazol-2-thiol, benzo[*d*]oxazol-2-amine, and benzo[*d*]thiazol-2-amine in compounds **7**, **8**, and **11** abolished *pqs* inhibitory activity (Table 1). On the contrary, the 1-methyl-1*H*-benzo[*d*]imidazol-2-amine derivative **6a** demonstrated a 15-fold enhancement of activity in the *P. aeruginosa* PAO1-L laboratory strain compared to **1**. By overlaying these structures, we speculated that the benzimidazole heterocycle and R_1_ substituent are important moieties that were responsible for this improved biological activity. Hence, compound **6a** provided a new starting point for a hit to lead study which focused on the following aspects: (1) exploration of the effect of the R_1_ substituent to gauge the steric and lipophilic requirements for optimal potency, (2) introduction of polar solubilizing functional groups to improve drug-like properties and (3) investigation of the effect of halogen substitution on the benzo[*d*]imidazole and phenylacetronitrile groups.

### Structure–Activity Relationship Study of the Benzo[*d*]imidazole-2 Amine Series

**Scheme 2:**
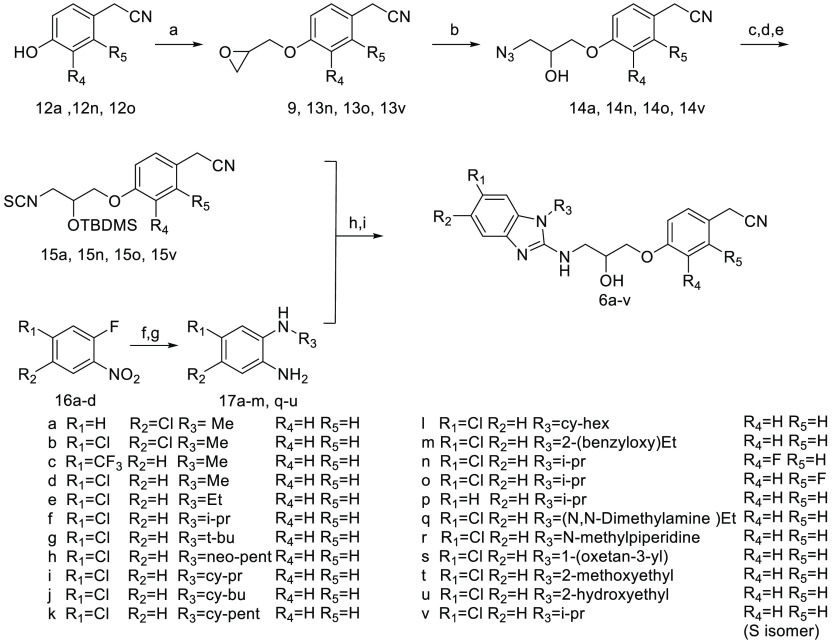
Synthetic Route for the Preparation of Benzo[*d*]imidazole Analogues **2a**–**v** Reagent and conditions: (a) eepichlorohydrin, Cs_2_CO_3_, CH_3_CN, reflux, 16 h, 25–28% or (*S*)-(−)-glycidyl nosylate for **13v**, Cs_2_CO_3_, CH_3_CN, reflux, 16 h 85–87%; (b) NaN_3_, NH_4_Cl, EtOH 40 °C, 16 h, 95–100%; (c) TBDMS-Cl, imidazole, DMF, rt, 16 h, 75–84%; (d) PPh_3_, 10% H_2_O: THF, rt, 16 h 79–90%; (e) di(1*H*-imidazol-1-yl)methanethione, DCM, rt, 16 h 21–25%; (f) R_3_-NH_2_, MeOH, reflux, 16 h, 90–95%; (g) Zn, NH_4_Cl, 10% CH_3_COOH; MeOH, rt, 1 h, 90–95%; (h) EtOH, reflux, 16 h, then DIC, Et_3_N, DMF, reflux, 16 h, 50–65%; (i) 10% TFA; MeOH, rt, 48 h 53–60%.

From initial modifications of the heterocyclic headgroup of **1**, it was evident that 2-amino-benzimidazole derivatives offered a significant improvement of potency and a new starting point for further optimization. However, the initial synthetic method suffered from both low yield and limited scalability despite its sustainability. Hence, a new synthetic strategy was used relying on the versatile coupling of two building blocks (Scheme 2): the isothiocyanate derivatives **15a**,**n**,**o**,**v** and the benzene-1,2-diamines **17a**–**m**,**q**–**u** followed by intramolecular cyclization mediated by *N*,*N*′-diisopropylcarbodiimide (DIC) to provide the desired products^15^**6a**–**v** after removal of the silyl protecting group.^16^

Isothiocyanate derivatives (**15a**, **15n**, **15o**, and **15v**) were synthesized starting from reacting epoxides (**9**, **13n**, **13o**, and **13v**) with sodium azide to afford (**14a**, **14n**, **14o**, and **14v**),^17^ which were subsequently protected with *tert*-butyldimethylsilyl chloride (TBDMS)^18^ and then reduced using the Staudinger procedure to the corresponding amines^19^ which upon reaction with di(1*H*-imidazol-1-yl)methanethione provided the desired isothiocyanates.^20^ In parallel, benzene-1,2-diamines **17a**–**m**,**q**–**u** were prepared from substitution of 1-fluoro-2-nitrobenzene derivatives **16a**–**d** with various primary amines followed by reduction of the nitro group using zinc and ammonium chloride..^21^

**Scheme 3:**
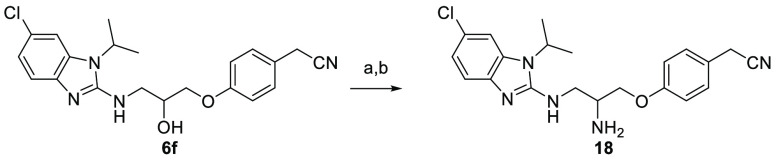
Preparation of Compound **18** with Amino Functionality Reagent and conditions: a) Ph_3_P, DIAD, diphenyl phosphoryl azide, THF (anhydrous), 45 °C, 16 h, 59%. b) Ph_3_P, 10% H_2_O: THF, rt, 16 h, 43%.

Finally, compound **18** was prepared from **6f** by a Mitsunobu reaction as outlined in Scheme 3.^22^

### *In Vitro* Evaluation of the Biological Activity of the PqsR Antagonists in *P. aeruginosa*

An extensive SAR was performed on **6a** to investigate the role of substitutions around various positions of the benzimidazole ring (groups R_1_–R_3_) as well as the aromatic tail (groups R_4_–R_5_) to fine-tune the potency of this series (**6a**–**u**) (Table 2). The activity of these analogues to inhibit the *pqs* QS system was evaluated in the PAO1-L and PA14 strains (that belong to the two major *P. aeruginosa* genomic lineages), both harboring a chromosomally integrated mCTX::P_*pqsA*_-*lux* transcriptional fusion to report for the activity of *pqs* system. Successful pharmacological blocking of AQ signal reception at the level of PqsR leads to a reduced transcription of the *pqsA-lux* genes and ultimately reduction in the luminescence readout.

**Table 2:**
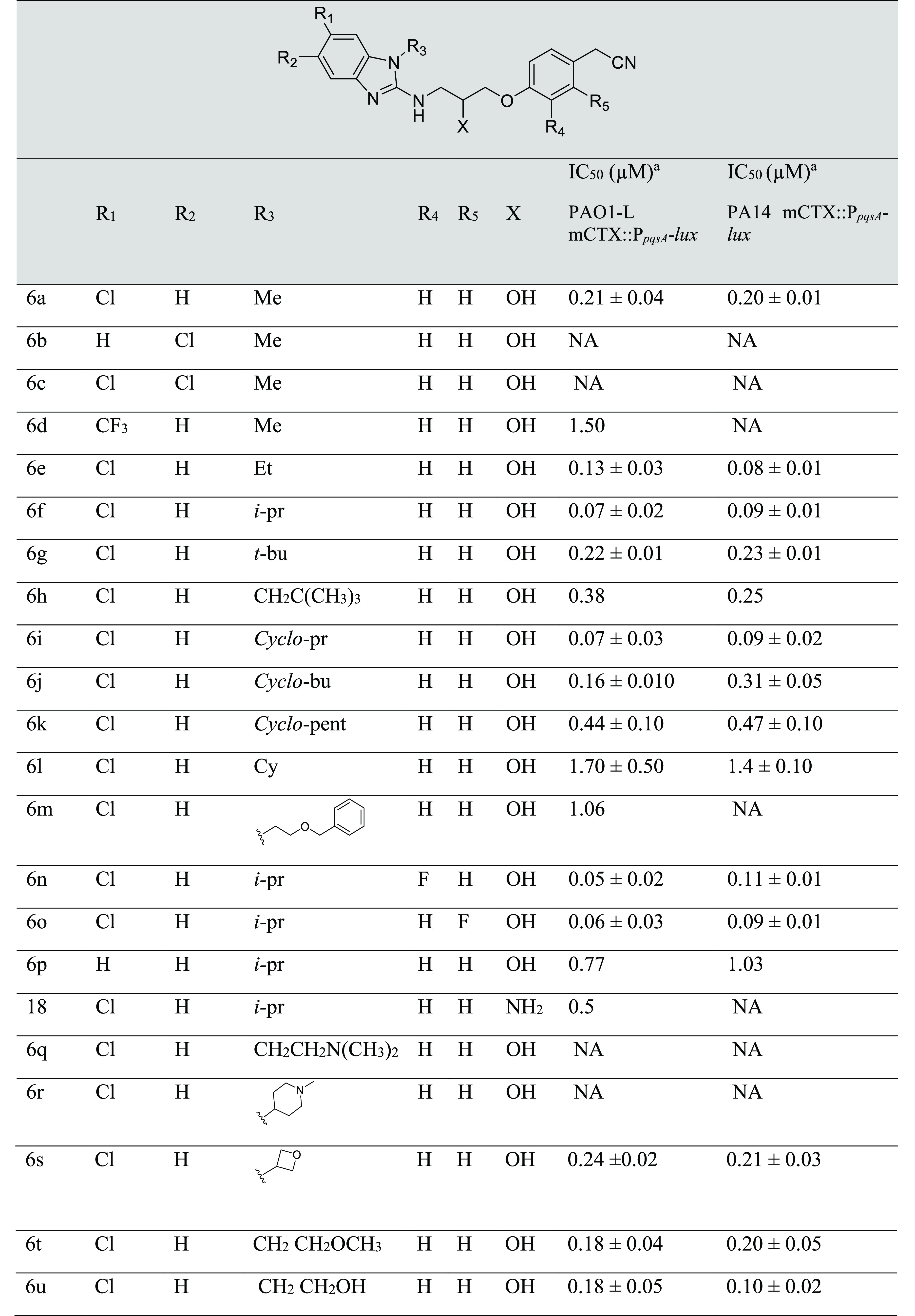
Structures and Activities of **2a**–**u** Analogues^b^

a Values are reported as mean ± SD of *n* = 2.

b NA refers to no activity at 10 μM. *pqs* inhibition and dose–response curves were determined in PAO1-L CTX::P_*pqsA*_-lux and PA14 CTX::P_*pqsA*_-lux strains.

The SAR initially investigated the effect of the chlorine atom position on potency of **6a**. To this end, moving the chlorine atom to position 5 in **6b** or introducing an additional chlorine atom as in **6c** abolished the activity in the PA bioluminescent reporter assay, while a trifluormethyl group substitution in **6d** led to 7-fold decrease in activity in the PAO1-L strain and complete loss of activity in PA14. Hence, it was concluded that substitution in the 6-position with a chlorine atom is important for biological activity and therefore this group was maintained throughout this study. Exploration of the R_3_ position proved informative as the size of the R_3_ group revealed a clear SAR for the analogues tested. For instance, compounds with an ethyl or isopropyl R_3_ substitution (**6e**, **6f**) demonstrated increasing levels of activity proportionate to the size of R_3_ particularly in PAO1 strain. In a similar fashion, cyclopropyl **6i**, cyclobutyl **6j**, and oxetane **6s** derived analogues exhibited comparable potencies to **6f** in both reporter strains. Increasing the R_3_ substituent size further led to decrease of activity relative to the substituent bulkiness as derivatives with neopentyl **6h** and cyclopentyl **6k** had approximately a 6-fold reduction in potency compared with **6f**. This effect was even more pronounced with bulkier groups exemplified in **6l** (R_3_ = hexyl, 25-fold reduction), **6m** (R_3_ = ethyloxymethylbenzene, 14-fold reduction) and **6r** (R_3_ = *N*-methylpiperidyl, not active). To enhance the physiochemical properties of the series through reducing lipophilicity, analogues with polar substituents were synthesized (**6t** R_3_ = methoxyethyl, **6u** R_3_ = hydroxyethyl, and **6q** R_3_ = dimethylaminoethyl). Compounds **6t** and **6u** were equipotent in PAO1-L with a 2-fold reduction in potency in relation to **6f**, however, a structurally related dimethylamino-ethyl substitution in **6q** obliterated the biological activity.

Considering the amino-alcohol linking group, replacing the alcohol group with a primary amine as in **18** gave a 7-fold reduction in potency in PAO1-L strain. This drop in activity was not investigated but could be associated with a change of membrane permeability or specific efflux mechanism.^23^ Despite its lack of activity in PA14, **18** represents an interesting candidate for prospective inhaled dosing owing to its basic nature.^24^

Finally, building on our previously reported series where we introduced a fluorine atom at the *meta- ortho-* of the phenyl ring in the aim to capture a further hydrogen bond interaction with the phenolic group of TYR^258^ (Supporting Information (SI), Figure S1).^13^ However, analogues (**6n** and **6o**) demonstrated similar biological activity to **6f** against PAO1-L.

Considering the aforementioned SAR study, **6f** was chosen as candidate for further pharmacological evaluation.

### Analysis of the Activity of the Enantiomers of **6f**

Following the synthetic route outlined in Scheme 2, compound **6v** (*S-*enantiomer) was synthesized using the desired enantiomerically pure epoxide **13v** (S isomer) as a starting material. **6v** was used later to determine the retention time of the *S-* enantiomer by employing analytical chiral high-performance liquid chromatography (HPLC). Subsequently, **6f** was separated into its enantiomerically pure enantiomers using HPLC with a lux-cellulose 4 chiral colum: **6v** (*S*- isomer) and **6w** (*R*- isomer) with enantiomeric excess (ee), 96.5 ± 0.8 and 91.2 ± 3.9%, respectively.

**Figure 2:**
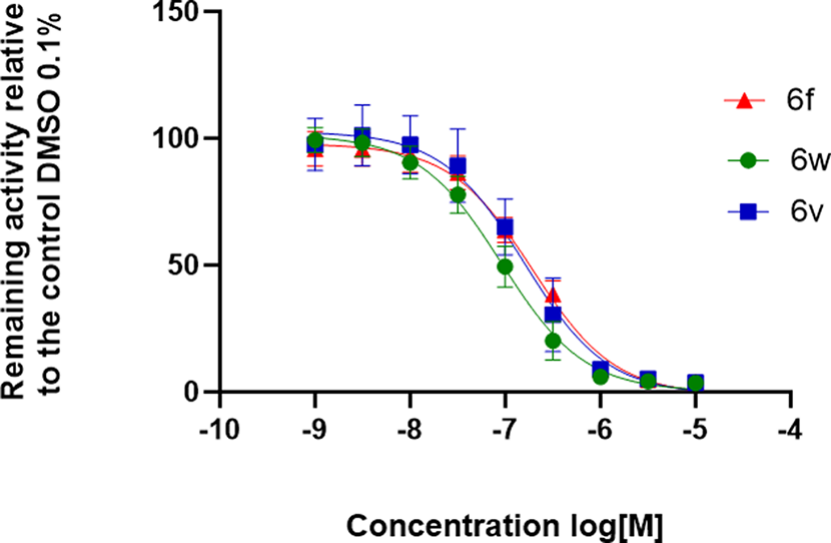
Dose–response curve for **6f** and its enantiomers **6v** and **6w** using the bioreporter strain PAO1-L mCTX::P_*pqsA*_-lux; *n* = 2.

Biological testing in PAO1-L showed that both enantiomers **6v** and **6w** have similar biological activities within the limit of the assay, and therefore, the racemic compound **6f** was used in the following pharmacological evaluations (Figure 2).

### Crystal Structure of Benzimidazole Derived Inhibitors with PqsR

**Figure 3:**
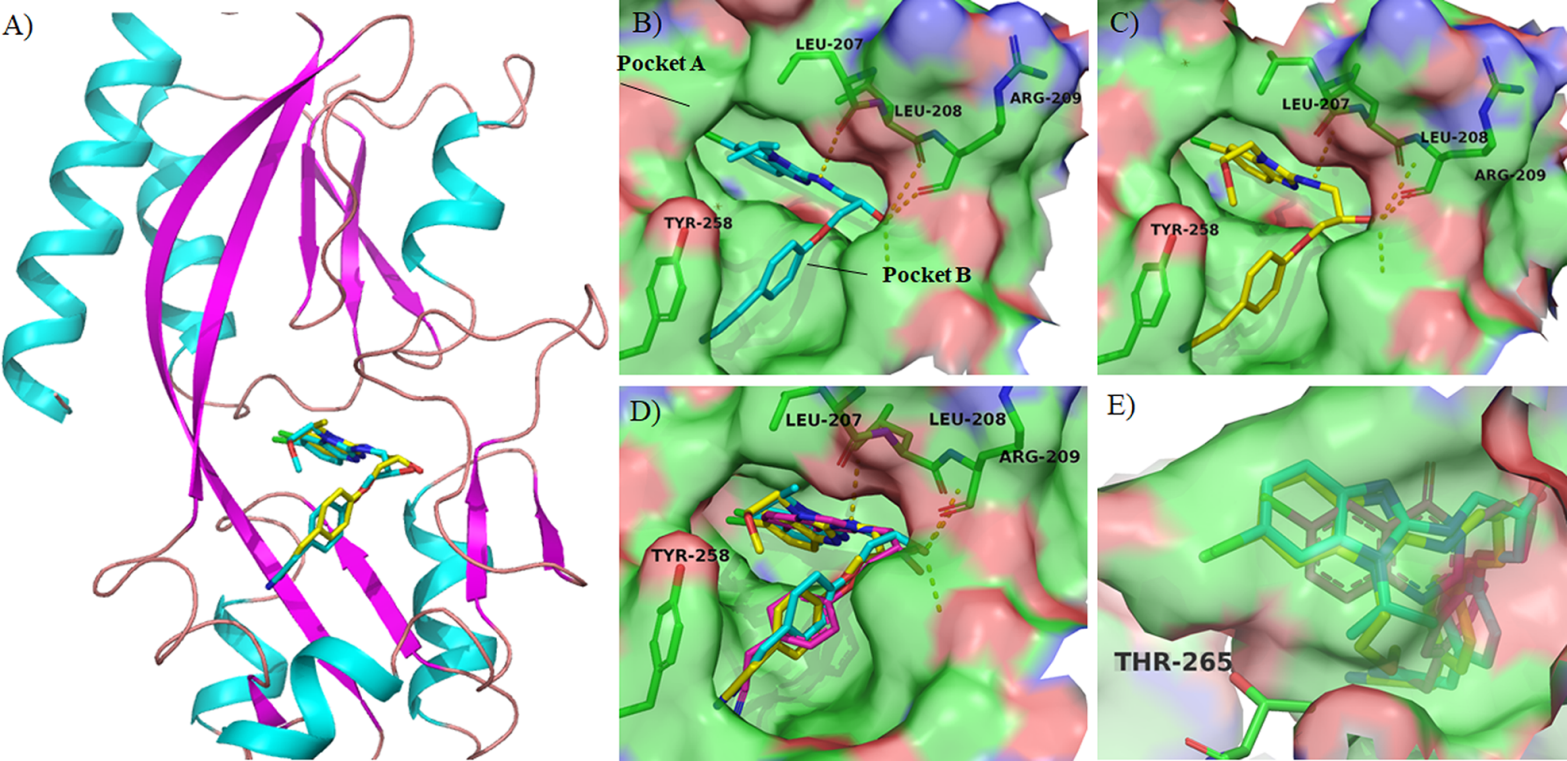
Schematic representation of crystal structure of PqsR^LBD^ complexed with PqsR antagonists: (A) Cartoon representation of PqsR^LBD^ complexed with compounds **6f** (cyan sticks and **6t** (yellow sticks). (B) Surface representation of the crystal structure of PqsR^LBD^ complexed with **6f** (cyan sticks), PDB 8Q5L at 2.9 Å. (C) Surface representation of the crystal structure of PqsR^LBD^ complexed with **6t** (yellow sticks), PDB 8Q5K at 2.8 Å. (D) Overlay of the crystal structures of **6f**, **6t**, and **1** (PDBs 8Q5L, 8Q5K, and 7O2T, respectively) (E) Close view of pocket A accommodating **6f**, **6t**, and **1**. Inhibition of AQ production.

X-ray crystallography techniques were employed in this study to gain a deeper understanding of the conformation and binding interactions of this series of inhibitors within the PqsR coinducer binding domain using crystal soaking experiments under the conditions reported by Soukarieh et al.^25^ Several reports noted that the PqsR receptor ligand binding domain consists of two subdomains known as pocket A (deep slot) and B (outer pocket) connected by an antiparallel β sheet hinge region (Figure 3A,B).^26,27^ The cocrystal structures of PqsR inhibitors reported to date revealed that the binding is dominated mainly by hydrophobic interactions in pocket A with additional hydrogen bonds with Leu^208^ or Gln^194^ in pocket B.^26^ In this study, cocrystal structures of **6f** and **6t** with the PqsR^LBD^ were obtained and revealed that both compounds bind in a similar arrangement and in comparable pose to the previous quinazolin-4(3*H*)-one series (Figure 3B–D), where the 2-amino benzo[*b*]imidazole and the 6-chloroquinazolin-4(3*H*)-one heterocyclic rings reside within pocket A with the 6-chlorine atom situated in close proximity to Thr^265^. Nevertheless, the chlorine on the benzo[*b*]imidazole ring is shifted slightly toward the Thr^265^ featuring an optimal conformation to fill the subpocket formed around the Thr^265^ region (Figure 3E). The *para*-phenyl acetonitrile group of **6f**, **6t** adopted a parallel position to the Tyr^258^ in pocket B to maintain hydrophobic interactions as the case in **1**. While the hydroxyl group position was altered slightly between the two series leading to form hydrogen bond with Leu^208^ and Arg^209^ in the benzimidazole derivatives compared to Leu^208^ and Glu^194^ in the quinazolinone derived inhibitor. However, unlike **1**,^13^**6f** and **6t** form an additional hydrogen bond between the amino group at the 2-postion of the benzimidazole ring and the backbone of of Leu^207^, which may contribute to the observed enhanced potency. Finally, the isopropyl substitution at *N*_1_ in the benzimidazole ring makes hydrophobic interaction with the lipophilic residues (Leu^207^, Leu^208^, Ile^236^, and Ile^263^) forming the lipophilic subpocket (Figure 3E)

### Effect of PqsR Inhibitors on AQ Production

**Figure 4:**
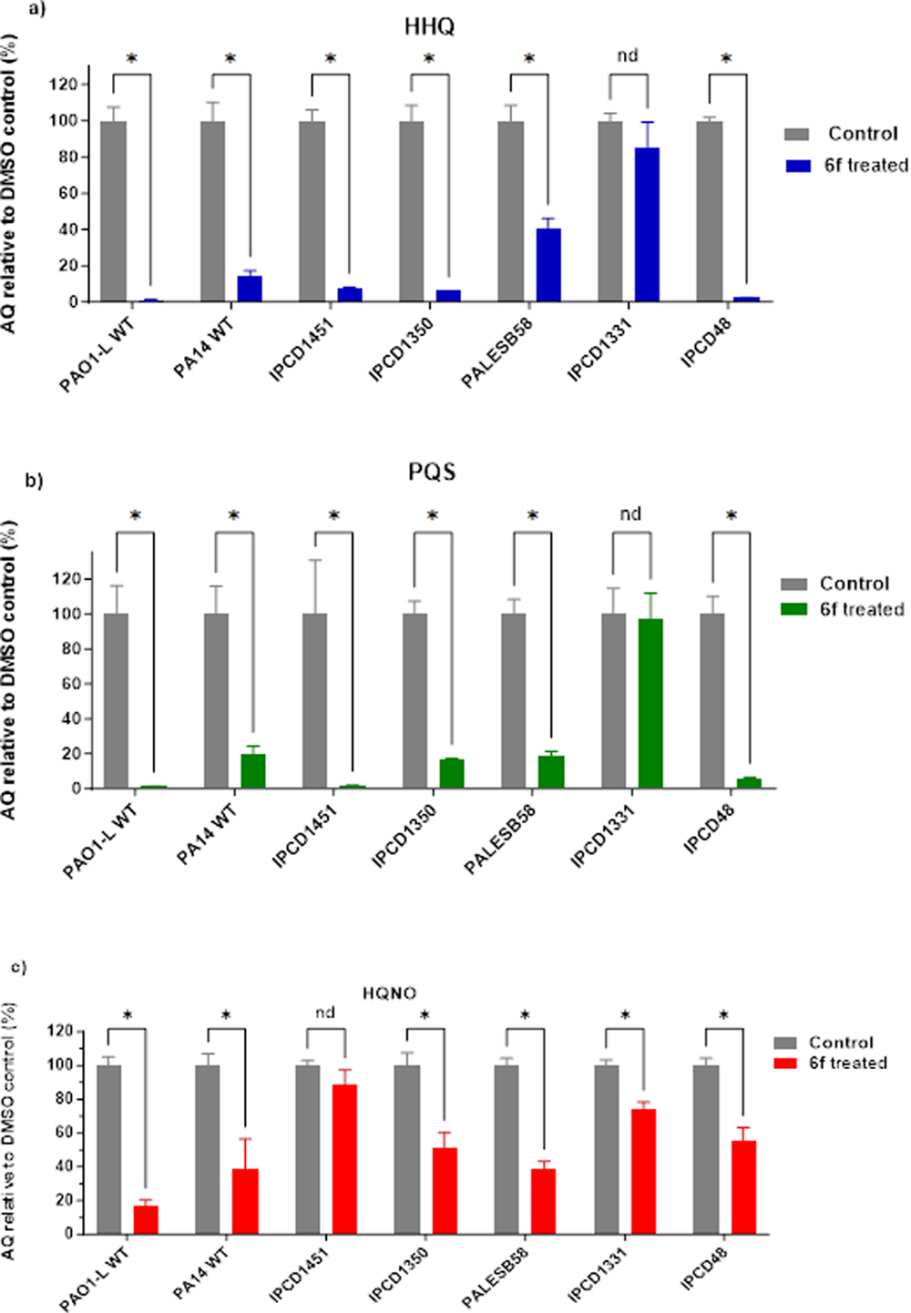
Quantification of AQ signals in various PA strains treated with 0.2 μM of **6f** in relative to DMSO vehicle control. (a) HHQ, (b) PQS, and (c) HQNO. Data are plotted as mean ± SD of *n* = 3.

PqsR directly regulates the biosynthesis of diverse AQs, of which HHQ and PQS act as QS molecules, while others such as 2-heptyl- 4-hydroxyquinoline *N*-oxide (HQNO) are potent cytochrome bc_1_ inhibitors that contribute to the environmental competitiveness of this pathogen.^9,28^ Previous studies have shown that PQS is a multifunctional iron chelator acting via PqsR-dependent and PqsR-independent pathways, contributing directly to iron acquisition and microvesicle formation.^29^ Upon binding to, and activating, PqsR, PQS, and HHQ both induce transcription of the *pqsABCDE* operon leading to elevated AQ levels, hence acting as an AI.^9^ PqsR inhibition reduces production of AQ, which can be quantified to serve as a direct readout for *pqs* system inhibition.^9^ To this end, **6f** was incubated with various *P. aeruginosa* laboratory strains and CF isolates and its impact on AQ production determined quantitatively using LCMS/MS and compared with untreated samples. HHQ and PQS levels were both substantially reduced in PAO1-L and PA14 laboratory strains that belong to each of the two main distinct phylogenetic groups that make up the population structure of *P. aeruginosa*) and in the IPCD48 CF isolate strain after treatment with 0.2 μM **6f** (Figure 4). While the LESB58 strain was less responsive to treatment, nevertheless, a moderate but significant reduction in AQ production was observed. On the contrary, IPCD1331 was the only strain in which no significant reduction in HHQ and PQS levels was observed. HQNO concentrations followed a slightly different trend where the reduction observed was less pronounced compared with HHQ and PQS with all strains showing good inhibition ranging between 20% and 60% apart from the IPCD1451 CF isolate which showed a nonsignificant reduction.

### Effect of PqsR Inhibitors on Pyocyanin Production

The production of the blue-green phenazine, pyocyanin, which is synthesized from chorismate via the multiple *phz* gene products, is positively regulated by PqsR.^30^ Pyocyanin is an active redox metabolite that promotes the generation of reactive oxygen species which contribute to the persistence of *P. aeruginosa* in the lungs of individuals with CF. It interferes with many physiological functions, including respiration, ciliary beating, epidermal cell growth, calcium homeostasis and prostacyclin release from lung endothelial cells.^31,32^

**Figure 5:**
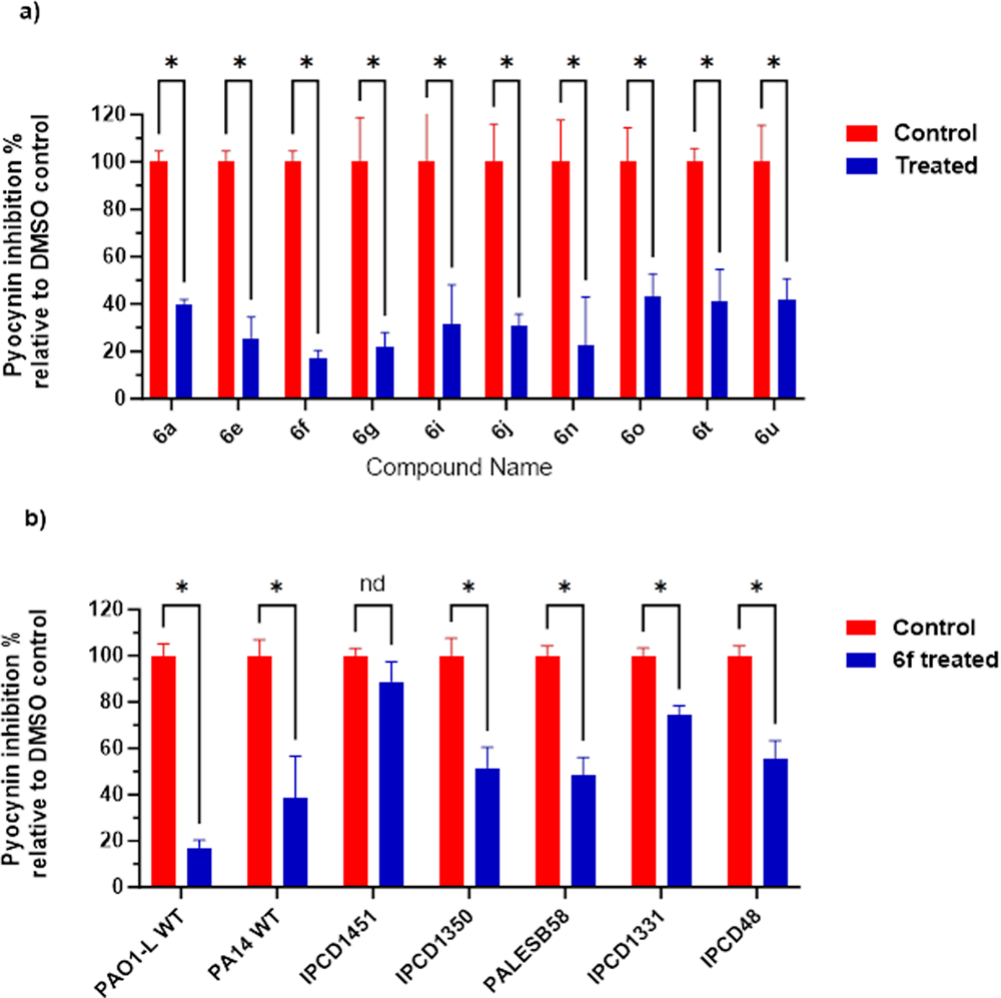
Inhibition of pyocyanin production by (a) the most active PqsR inhibitors at 3-times IC_50_ relative to the DMSO negative control and (b) **6f** at 200 nM for different PA strains. Data are plotted as mean ± SD of *n* = 3.

Here, the most active PqsR inhibitor analogues were evaluated for their effect on pyocyanin production in PAO1-L (Figure 5a). All the compounds tested inhibited pyocyanin production by >50% when used at a concentration equivalent to three times the IC_50_. In particular, PqsR inhibitors **6f** and **6g** substantially reduced pyocyanin production by ∼80%. This assay was extended further to investigate the effect of **6f** on pyocyanin production in different *P. aeruginosa* at **6f** concentration of 200 nM (Figure 5b). Interestingly, **6f** inhibited pyocyanin production in all strains except for IPCD1451 which showed nonsignificant inhibition.

### Effect of **6f** on the Antibiotic Tolerance of PA Biofilms

**Figure 6:**
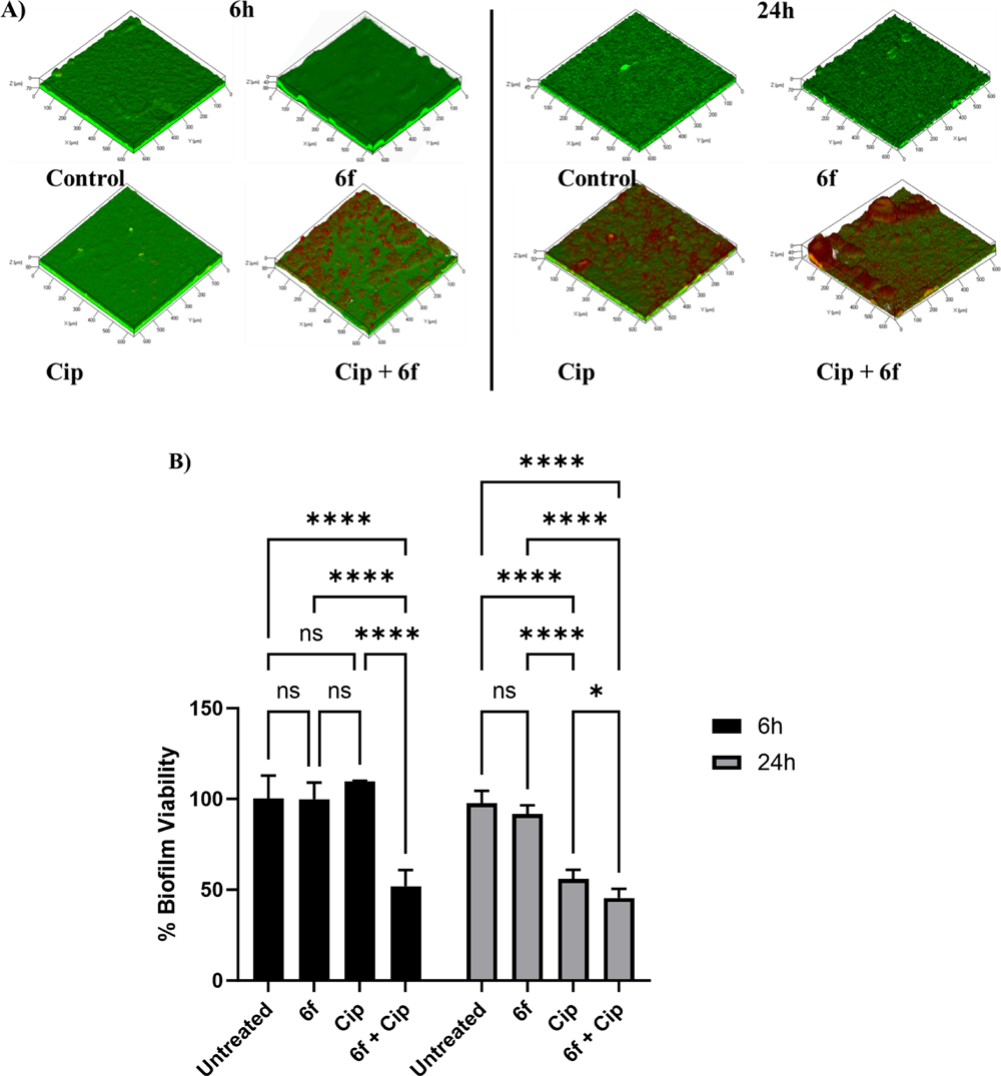
(A) Representative CLSM 3D Z-stack images of PAO1-L biofilms after 24 h growth with or without **6f** and further treatment for 6 or 24 h with no treatment (control), **6f** (10 μM), ciprofloxacin (Cip: 60 μg/mL), or a combination of **6f** and Cip (10 μM and 60 μg/mL, respectively). Live bacteria are depicted in green (SYTO9 dye) and dead cells are shown in red color (propidium iodide stain). (B) Biofilm viability assay. Bar chart showing PAO1-L biofilm viability quantified after treatment with different conditions for 6 or 24 h. The concentrations of the drugs used were ciprofloxacin 60 μg/mL (Cip) and **6f**, 10 μM. The statistical analysis was performed using 2-way ANOVA analysis (GraphPad 9.0).

Biofilms are bacterial communities that are highly tolerant to antibiotics.^33^ The *pqs* QS system has been shown to regulate PA biofilm development. Therefore, pharmacological interference with the *pqs* machinery could sensitize PA biofilms to antibiotics.^34^ To evaluate this effect, PAO1-L biofilms were grown for 48 h in the presence of **6f** (method 1). Following treatment with the broad-spectrum antibiotic ciprofloxacin (Cip), the viability of biofilm bacteria was determined using LIVE/DEAD BacLight staining and confocal laser scanning microscopy (CLSM). The effect of **6f** on biofilm viability as a single treatment or in combination with subinhibitory concentration of Cip was examined at two different time points (6 and 24 h after treatment) to establish whether this PqsR antagonist enhanced the activity of the antibiotic. **6f** alone had no significant effect on biofilm viability, however, when combined with Cip a significant potentiation of antibiotic activity was apparent at both time points examined (Figure 6). It is noteworthy that the effect was greater at 6 h treatment compared with 24 h.

**Figure 7:**
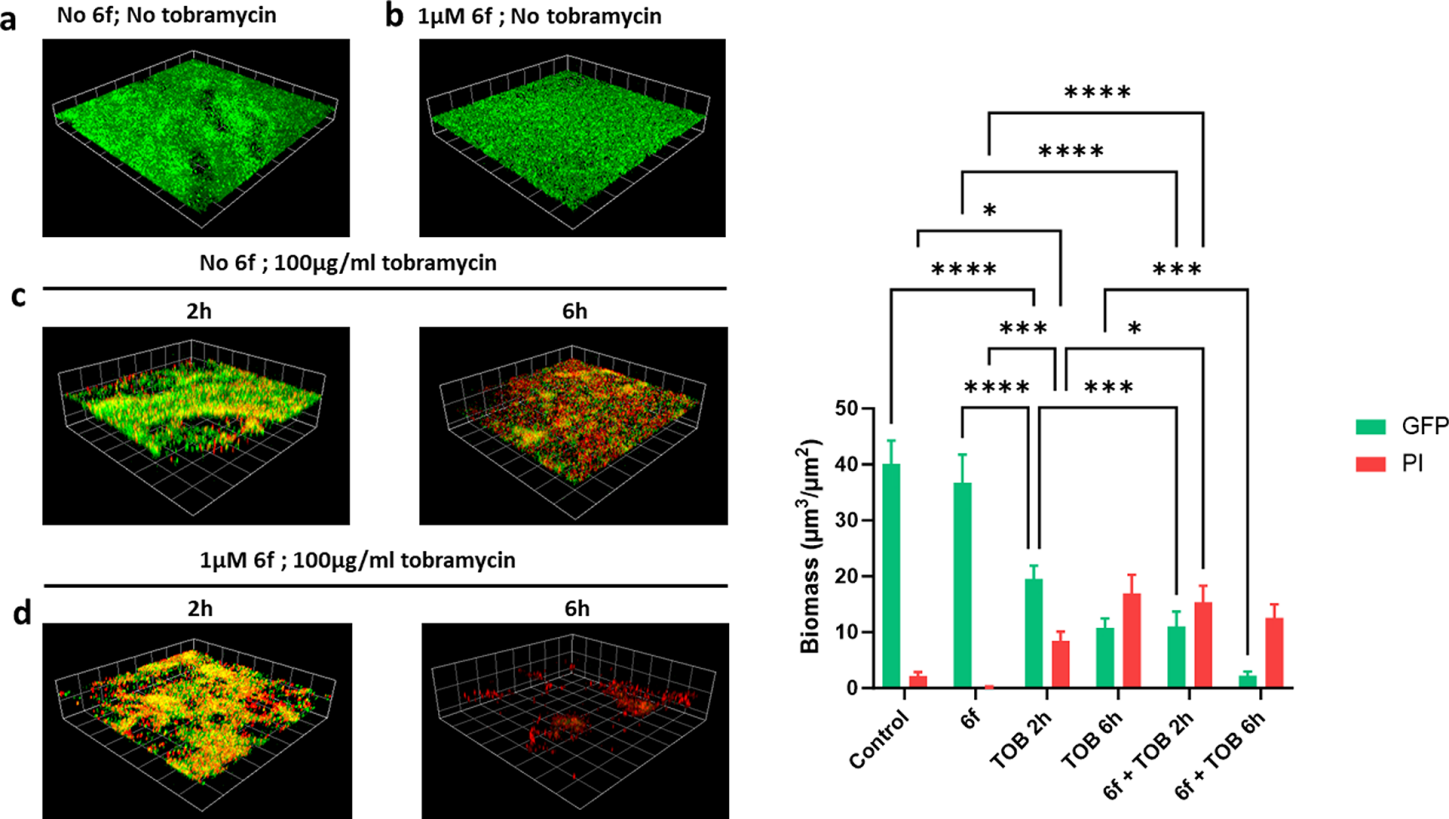
Effect of **6f** and tobramycin on GFP-labeled *P. aeruginosa* PAO1-L biofilms. (A) Representative CLSM images: (a) Untreated biofilm; (b) biofilm grown with **6f** at 1 μM for 16 h; (c) biofilm treated with 100 μg/mL tobramycin for 2 or 6 h after 16 h of growth; (d) biofilm grown with 1 μM **6f** and treated with (**6f** 1 μM), tobramycin (100 μg/mL) for 2 or 6 h after 16 h of growth. Dead cells and extracellular DNA were stained red with propidium iodide (PI). Three-dimensional (3D) sections and cross sections are shown. Scale bar represents 100 μm. (B) Quantitative analysis of biofilm biomass at different conditions of treatment compared with a solvent vehicle control.

Biofilms of PAO1-L were also tested for the impact of **6f** on the potentiation of tobramycin activity (method 2). Figure 7 shows that **6f** did not impact on the viability of preformed biofilms on its own, but when combined with tobramycin, it enhanced the killing at both 2 and 6 h of incubation with no live cells present at the later time point.

### Assessment of the Cytotoxicity of **6f** and **6n**

**Figure 8:**
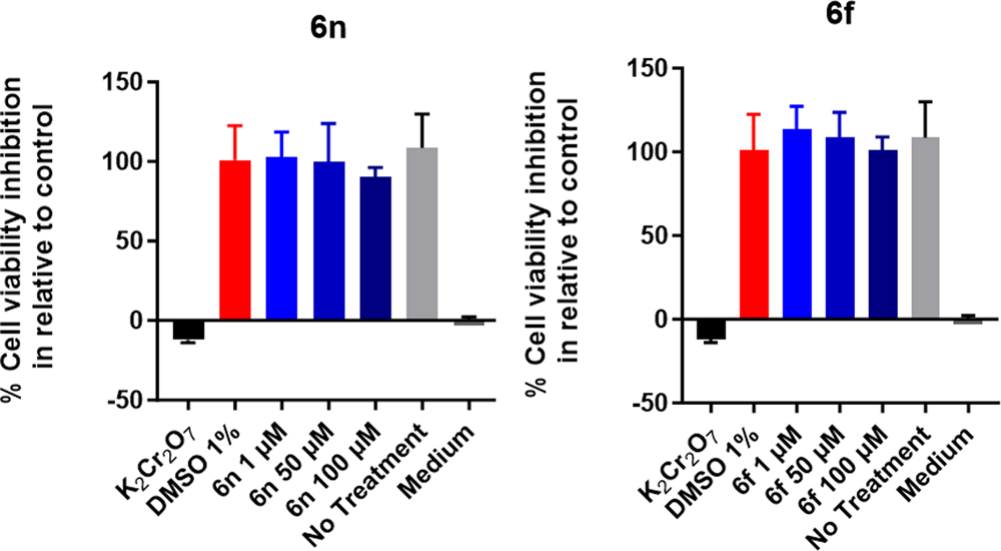
A549 human lung epithelial cell cytotoxicity assay. **6f** and **6n** were evaluated at three different concentrations compared with 1% DMSO as a negative control. Potassium dichromate was used as a positive control. Data are plotted as mean ± SD of *n* = 3.

Establishing the safety profile of new chemical entities is paramount for further drug development. Hence, the effect of two inhibitors (**6f** and **6n**) on their cytotoxicity for A549 human lung epithelial carcinoma cell lines was determined. The assay employed resazurin reduction, a sensitive fluorometric assay widely used as a standard methodology in drug discovery research.^35^ This assay relies on the ability of living cells to reduce resazurin to the fluorescent compound, resorufin, which indicates the rate of the metabolic activity as a means to quantify cell viability. The results obtained suggest that both **6f** and **6n** are not cytotoxic at concentrations up to 100 μM, which indicates a promisingly broad therapeutic index for both compounds (Figure 8).

## Discussion and Conclusions

This hit to lead study resulted in the discovery of a new potent benzimidazole derived series based on the installation of the 1*H*-benzo[*d*]imidazol-2-amine group as a replacement for the quinazolin-4(3*H*)-one from our previously published PqsR antagonist (**1**).^13^ Following optimization of the synthetic procedures, a comprehensive SAR highlighted compound **6f** (IC_50_ = 70 nM), which subsequently formed the focal point for this study.^13,36,37^ The size of the isopropyl substituent was shown to be optimal for the biological activity as smaller or larger substituents led to decrease in compound activity. Integration of a hydroxyethyl substituent in **6u** led to the improvement of calcdulated lipophilicity scores (log *P*: **6u**,2.5; **6f**: ,3.8; predicted using Instant J Chem) associated with a 2-fold loss in activity. The chiral resolution of **6f** revealed that both enantiomers (**6v**, **6w**) demonstrated comparable activity to the racemic compound **6f**. In a similar fashion to our previous findings, the chlorine atom at the 6-position of the benzo[*d*]imidazole ring proved important for biological activity. The study subsequently focused on the examination of the structural aspects of the inhibitor–receptor complex. To achieve this aim, two crystal structures of inhibitors (**6f** and **6t**) complexed with the PqsR^LBD^ were obtained. These revealed that both compounds bind in a similar manner to our previously reported PqsR inhibitor **1**(25) except for an additional interaction between the 2-amino group and Arg^209^, which may have contributed to the enhancement of activity noted for the current series of compounds. The crystal structure also corroborated our rational design that the lipophilic subpocket around (Leu^207^, Leu^208^, Ile^236^, and Ile^263^) can be exploited with lipophilic substituent at position 1 of the benzimidazole ring. However, introduction of bulkier groups than isopropyl resulted to decrease or abolishment of the activity which could be due to the limited size of this subpocket.

The next stage of this study focused on the phenotypic analysis of **6f**, which provided robust evidence of substantial *pqs* system inhibition at low concentrations (<1 μM) of **6f** manifested by reduction of AQs (PQS, HHQ, HQNO) and pyocyanin production in laboratory *P. aeruginosa* strains. It is noteworthy that the validation of activity of any antimicrobial treatment on strains from different origins and genomic content is essential to avoid costly pitfalls in further drug development, an aspect that has often been overlooked in preclinical antibacterial discovery research.^38^ Hence, these assays were also performed on a panel of *P. aeruginosa* isolates belonging to different groups^39^ to show that **6f** maintained its activity in a wide range of strains except for IPCD1331 and IPCD1451. The reasons behind the differential responses of these strains were not further pursued but may be linked to variations in the level of *pqs* activity or due to membrane permeability issues or to up-regulated multidrug efflux pumps.^23^

The impact of **6f** on enhancing the sensitivity of PA biofilms to antibiotics was then studied as, in this surface associated lifestyle, PA is highly resilient to antimicrobial action. The effect of **6f** alone or in combination with antibiotics on biofilm viability was investigated. As with all PA QS inhibitors, their effect on biofilm viability was negligible, however, their potentiation of the antibiofilm effect was clearly evident. For ciprofloxacin the impact was greater after 6 h of treatment and to a lesser extent at 24 h. In contrast, for tobramycin, the effect was greater after 6 h than 2 h of incubation. The reasons behind the different results obtained with the two antibiotics may be related to their different modes of action or the biofilm models used. In the case of ciprofloxacin there is a possibility that the antibiotic may have attained the maximal effect after 24 h masking the synergistic activity of **6f**.

Up to this point, **6f** appeared as a promising candidate for further *in vivo* testing to compromise PA virulence and hence infection, an aspect that could be particularly beneficial for antibiotic resistant strains within the complex environment of, e.g., the CF lung,^40^ where it is common practice to use inhaled therapies for localized enhanced delivery of treatments and to avoid systemic exposure and unnecessary toxicity or adverse effects.^41^ Therefore, the study proceeded to evaluate compound **6f** stability, toxicity, and its pharmacokinetic profile after lung administration. The cytotoxicity of **6f** for eukaryotic cells was determined and a safe cytotoxic profile established for A549 lung epithelial cells up to 100 μM.

The hepatic stability of **6f** was investigated in our previous report;^42^**6f** performed inadequately in hepatic stability testing when exposed to human and rat microsomes where the **6f** half-life was determined to be relatively short. These results were further corroborated in a PK profiling study of **6f** following intratracheal administration in rats as a coarse suspension. Compound **6f** (previously reported as SEN089)^42^ showed a rapid clearance from lung to plasma as well as fast systemic clearance indicating a lower potential for further *in vivo* testing. Previous research by Scütz et al. reported reduction of bacterial load in murine mucoid lung infection model with their QSI4 PqsR inhibitor despite its short half-life of approximately 0.85 h.^43^ Alternative approaches for the delivery of these inhibitors are currently being sought that enhance both delivery and retention of the PqsR antagonist such as employing nanoparticles and polymers to control drug release.^44^ Another alternative would be the use of inhibitors with a basic nature as **16** which may exhibit enhanced binding to lung tissues and therefore a longer half-life.^24^

## Experimental Procedures

Chemical synthesis: commercially available starting materials, reagents and solvents were purchased from commercial sources (Sigma-Aldrich, Alpha Aesar, Fisher Scientific, or Fluorochem), and used without further purification. Nuclear magnetic resonance*:*^1^H NMR and ^13^C NMR, were obtained at rtusing a Bruker AV400, spectrometer operating at 400 MHz. The samples were prepared in deuterated solvent; DMSO-*d*_6_. Chemical shifts (δ) were recorded in ppm relative to trimethylsilan (TMS) and coupling constants (*J*) were recorded in Hz. Abbreviations used in the description of spectra are s (singlet), br (broad), d (doublet), dd (doublet of doublets), t (triplet), q (quartet), sp septet, and m (multiplet). The spectra were analyzed using Topspin 3.0 software. Mass spectrometry: LCMS data were recorded on a Shimadzu UFLCXR HPLC system coupled to an Applied Biosystems API2000 electrospray ionization mass spectrometer (ESI-MS). The column used was a Phenomenex Gemini-NX 3 μm 110 A° C-18, 50 mm × 2 mm thermostated at 40 °C. The flow rate was 0.5 mL/min of a solvent system of increasing gradient over 5 min of acetonitrile (5–95%) in water, each containing 0.1% formic acid. UV detection was at 220 and 254 nm. *m*/*z* values are reported in Daltons to one decimal place and retention times (*t*_R_) are provided in minutes to two decimal places. All final compounds reported here have purity of over 95% when analyzed by LCMS. All high-resolution mass spectra (HRMS)- time-of-flight electrospray were recorded on a Waters 2795 spectrometer by electrospray ionization (TOF ES) and the LC-MS spectra were performed on a Shimadzu UFLCXR system coupled to an Applied Biosystems API2000, and visualized at 220 nm (channel 2) and 254 nm (channel 1). Chromatography*:* Thin-layer chromatography (TLC) was performed, UV light, and standard TLC stains were used to visualize the Merck Silica gel 60 A ˙F254 plates. Compounds were purified via column chromatography using either a peristaltic pump or normal phase Interchim Puriflash prepacked cartridges consisting of 50 μm silica, or a glass column using Merck Geduran silica gel 60 A (230–240 μm). Column size selected was generally 40–60 times the loading quantity. Chiral HPLC method: An isocratic gradient of 15:85/ethanol:hexane over 20 min at a flow rate of 2 mL/min. Eluent detection was monitored by UV absorbance at 254 nm on a Dionex UltiMate 3000 system with a Lux Cellulose-4 column (250 mm × 4.6 mm, Phenomenex).

### Bacterial Strains and Growth Conditions

The *P. aeruginosa* strains and plasmids used in this study are shown in SI, Table S1. Bacteria were grown in lysogeny broth (LB) at 37 °C, unless stated otherwise. Where required, tetracycline (Tc) was added to the medium at 125 μg/mL. Synthetic alkylquinolones were added at the concentrations indicated.

### Bioluminescent Reporter Assay

Strains PA14 mCTX::P_*pqsA*_-*lux* and PAO1-L mCTX::P_*pqsA*_-*lux* were used to detect PqsR-controlled activation of the *pqsA* promoter, as previously described,^45^ and the assay was performed according to a published method.^46^ For initial screening, the compounds were tested at a concentration of 10 μM, which was prepared from a 10 mM stock, in DMSO.

### Pyocyanin Quantification

The experiment was performed following a published protocol with minor modifications. Strains were cultured into 5 mL of fresh medium overnight. Compounds were assayed at 3 × IC_50_s concentration, for 16 h, at 37 °C (Kuhner LT W Shaker, Adolf Kuhner AG, Basel, Switzerland). Cells were centrifuged at 10 000 RCF for 10 min (Allegra 64R centrifuge, Beckman Coulter, High Wycombe, UK), and the supernatant was transferred to 15 mL falcon tubes with a HSW 10 mL Soft-Ject Syringe and a 0.22 μM Sartorius syringe-driven filter (Fisher Brand, Loughborough, UK). Pyocyanin pigment was extracted into chloroform by mixing 7.5 mL of supernatant with 4.5 mL of chloroform. Pyocyanin was further extracted into 1.5 mL of 0.2 M HCL, which gave a pink/red solution, and the absorbance was measured at 520 nm.^47^

### Alkyl Quinoline Quantification

For each test sample, 100 μL of sterile filtered supernatant was spiked with 10 μL of an internal standard solution (10 μM d4-PQS in MeOH) and diluted with water to a total volume of 500 μL. Samples were then extracted three times with an 0.5 mL aliquot of ethyl acetate, vortex mixing the aqueous/organic mix for 2 min, then removing the organic phase once the layers had successfully partitioned. For each sample, the combined organic extracts were dried under vacuum and redissolved in 100 μL of MeOH prior to analysis. For the LC-MS/MS analysis of supernatant extracts, the chromatography was achieved using a Shimadzu series 10AD VP LC system (Columbia, MD, USA). The LC column, maintained at 40 °C, was a Phenomenex Gemini C18 (3.0 μm, 100 mm × 3.0 mm) (Macclesfield, Cheshire, UK) with an appropriate guard column. Mobile phase A was 0.1% (v/v) formic acid in water containing 2 mM 2-picolinic acid, and mobile phase B 0.1% (v/v) formic acid in methanol. The flow rate throughout the chromatographic separation was 450 μL/min. After an injection of a 2 μL/sample, a binary gradient, beginning initially at 30% B, increased linearly to 99% B over 5 min. The composition remained at 99% B for 3 min, decreased to 30% B over 1 min, and stayed at this composition for 4 min, to allow for column equilibration. The MS system used for analyte detection was an Applied Biosystems Qtrap 4000 hybrid triple-quadrupole linear ion trap mass spectrometer (Foster City, CA, USA), equipped with an electrospray ionization (ESI) interface. Instrument control, data collection, and analysis were conducted using Analyst software (Foster City, CA, USA). The MS analysis was achieved with positive electrospray (+ES) multiple reaction monitoring (MRM) screening of the LC eluent for specific AQ analytes. Where chromatographic peaks for HHQ, HQNO, and PQS were detected, a peak area was determined, and analyte peak area/internal standard peak area calcdulated.

### Biofilm Viability Assay

#### Method 1

Biofilms were grown on glass coverslips (13 mm Ø, no. 1.5 thickness) in a rolling biofilm bioreactor system^48^ (20 rpm) in FAB 10 mM glucose medium, inoculated with diluted (OD600 nm = 0.01) bacteria from overnight cultures in LB. For the biofilm samples that were treated with **6f**, a concentration of 10 μM was supplemented to the bioreactor’ media at the start of the experiments. The biofilms were cultivated at 30 °C for 24 h, then washed in PBS to remove loosely attached cells and incubated for a further 6 or 24 h in fresh medium supplemented with various treatments. These included free ciprofloxacin 60 μg/mL (× 300 the MIC of planktonic *P. aeruginosa* cells,^44^**6f** at 20 μM and ciprofloxacin in combination with **6f**. Biofilms exposed to each treatment were washed in PBS, and the viability of attached cells was evaluated by fluorescent staining using the LIVE/DEAD BacLight bacterial viability kit (Molecular Probes, Life Technologies) according to manufacturer instructions. Following staining, coverslips were rinsed with distilled water and imaged using a LSM700 AxioObserver (Carl Zeiss, Germany) confocal laser scanning microscope (CLSM). Viable and nonviable biofilm biomass quantification from image stacks of biofilms was done with Fiji-ImageJ software. Live/dead ratios were established for each treatment and compared to untreated controls.

#### Method 2

Biofilms were cultivated on borosilicate glass coverslips in Petri dishes. *P. aeruginosa* strains, PAO1-W, was transformed with plasmid pMMG, which constitutively expresses GFP from the Ptac promoter.^50^ The tagged strain was grown at 37 °C, for 16 h, in 2 mL of RPMI-1640 (Lonza, Slough, UK), supplemented with 20 mM d-glucose (Sigma–Aldrich, Dorset, UK) and 2 μM FeCl_3_ (Sigma–Aldrich, Dorset, UK). Cultures were diluted 1:100 in fresh medium and allowed to grow for a further 4 h, or until an OD600 of 0.5 was reached. The mid logarithmic cultures were diluted to an OD600 of 0.01 in 25 mL of RPMI, supplemented with glucose and FeCl_3_, and inoculated into Petri dishes containing UV sterilized borosilicate glass coverslips (22 mm × 22 mm, thickness no1) (VWR, Lutterworth, UK). Bacterial cells were seeded at 37 ◦C under static conditions for 1.5 h, and compound **6f** was supplemented to the **6f** treated samples (**6f**, **6f** + Tob samples) at a concentration of 1 μM before dishes were transferred to a shaker at 60 rpm and 37 ◦C for 16 h to form mature biofilms. Tobramycin and propidium iodide were added to the 16 h old cultures at concentrations of 100 μg/mL and 2 μM, respectively, followed by further incubation for 2 or 6 h. Coverslips were examined under a laser scanning fluorescent microscope (LSM2, Zeiss, Oberkochen, Germany). Biofilms were visualized using egfp mode at an excitation wavelength of 488 nm with emission wavelength of 510 nm. Imaging was carried out using Zen 2011 imaging software (Zeiss, Oberkochen, Germany). A total of 5 Z-stacked images were collected per coverslip. Sampling was conducted at random from the central portion of each coverslip.

#### Cytotoxicity Study

Cell viability was assessed according to Nimesh et al.^51^ on A549 adenocarcinomic human alveolar basal epithelial cell line. The viability of the A549 cell line was assessed after overnight incubation with three concentrations (1, 50, and 100 μM) of the corresponding compound using alamar blue (resazurin) dye. Cells were maintained in the Dulbecco’s Modified Essential Medium (DMEM) supplemented with 10% fetal bovine serum (FBS) and 1% penicillin–streptomycin–neomycin (PSN). After that, 100 μL of suspended cells with a final concentration (1 × 10^4^ cells/wall) were seeded in 96-well plates with 100 μL of (1, 50, 100 μM) concentrations of the tested compound was dispensed in sextuplicate. In addition, the highest concentration of the solvent vehicle (1% DMSO) was employed as a negative control; 100 μM of K_2_Cr_2_O_7_ was added as a positive control. The plate was then incubated at 37 °C, 5% carbon dioxide for 24 h. After 24 h of incubation, 20 μL of alamar blue dye was added to the corresponding wells. The plates were further incubated for 4–6 h and the intensity of the fluorescence was measured using an excitation light of 510 nm and measuring the fluorescence output at 590 nm. The cell viability in each well was normalized to the DMSO reading.

#### Crystallography

Protein samples were prepared as reported before in Ilangovan et al.^26^ PqsR94-309 was produced in BL21 (DE3) and purified by Ni-NTA chromatography and size exclusion using a S75 16/60 in a running buffer of 20 mM Tris-HCl and 150 mM NaCl (pH = 7.4). Protein was concentrated to 6 mg/mL and used to set up 24 well sitting and hanging drops. Crystals grew in 0.1 M Ttrisodium citrate (pH range 5.8–6.2), 0.2 M ammonium acetate, and MPD (3–8%). Antagonist was introduced by soaking the crystals in excess ligand (>10×) for 24 h prior to cryo-cooling. To aid solubility, ligands were dissolved in a multicomponent solvent mixture (Ciccone, 2015). Diffraction experiments were performed on i24 and i04. Data was processed with DIALS and reduced with AIMLESS. Molecular replacement was performed with PHASER, ligand fitting with COOT, and refinement completed with REFMAC and PHENIX.

#### Data Management and Analysis

Instant JChem was used for structure database management, Search and Prediction, Instant JChem 16.2.15.0 2016, ChemAxon (http://www.chemaxon.com). Sigmoidal dose–response curves and the representation of all data were prepared using GraphPad Prism 9.0.2. Molecular modeling was performed using OpenEye Scientific Software Inc.^52^ and Schrödinger Suite (Schrödinger Release 2023-3: Glide, Schrödinger, LLC, New York, NY, 2023)

#### Analytical Data

##### Preparation of 2-(4-(3-Azido-2-hydroxypropoxy)phenyl) Acetonitrile (**14a**)

To a solution of 2-(4-(oxiran-2-yloxy)phenyl) acetonitrile **9** (5 g, 0.03 mol) in EtOH (100 mL) was added NaN_3_ (5 g, 0.08 mol) and NH_4_Cl (3.2 g, 0.06 mol). The mixture was stirred at rt overnight. The reaction was then concentrated to dryness, and the residue was dissolved in ethyl acetate and washed with water. The organic phase was concentrated to afford the desired product as a colorless oil, which was used in the next step without further purification. Colorless oil (6 g, 98% yield). ^1^H NMR (400 MHz, DMSO-*d*_6_) δ 7.31–7.22 (m, 2H), 7.01–6.95 (m, 2H), 5.56 (d, *J* = 5.2 Hz, 1H), 4.01 (m, 1H), 3.97–3.89 (m, 4H), 0.86 (d, *J* = 2.6 Hz, 1H), 0.14–0.01 (m, 1H). ^13^C NMR (101 MHz, DMSO-*d*_6_) δ 158.29, 129.77, 129.77, 123.72, 119.97, 115.43, 115.43, 69.89, 68.75, 53.70, 22.02.

##### Preparation of 2-(4-(3-Azido-2-hydroxypropoxy)-3-fluorophenyl) Acetonitrile (**14n**)

The title compound was prepared in similar manner as described for **14a** using **13n** (1 g, 4 mmol) as the starting material. Colorless oil (1.1 g, 92% yield). ^1^H NMR (400 MHz, DMSO-*d*_6_) δ 7.27–7.18 (m, 2H), 7.13 (dd, *J* = 8.8, 2.0 Hz, 1H), 5.61 (dd, *J* = 5.0, 2.4 Hz, 1H), 4.03 (dt, *J* = 4.1, 2.0 Hz, 3H), 3.97 (s, 2H), 3.44–3.34 (m, 2H). ^13^C NMR (101 MHz, DMSO-*d*_6_) δ 151.95 (d, *J* = 247.2 Hz), 146.18 (d, *J* = 10.2 Hz), 124.98 (d, *J* = 2.7 Hz), 119.58, 116.49 (d, *J* = 19.7 Hz), 116.04 (d, *J* = 1.5 Hz), 70.98, 68.64, 53.62, 21.90.

##### Preparation of 2-(4-(3-Azido-2-hydroxypropoxy)-2-fluorophenyl) Acetonitrile (**14o**)

The title compound was prepared in similar manner as described for **14a** by utilizing **13o** (1 g, 4 mmol) as the starting material. Colorless oil (1.1 g, 92% yield). ^1^H NMR (400 MHz, DMSO-*d*_6_) δ 7.36 (t, *J* = 8.8 Hz, 1H), 6.99–6.79 (m, 2H), 5.58 (d, *J* = 5.1 Hz, 1H), 4.09–3.98 (m, 1H), 3.95 (s, 4H), 3.46–3.35 (m, 2H). ^13^C NMR (101 MHz, DMSO-*d*_6_) δ 161.13 (d, *J* = 208.5 Hz), 159.88 (d, *J* = 26.1 Hz), 131.32 (d, *J* = 4.9 Hz), 118.82, 111.72 (d, *J* = 3.1 Hz), 110.61 (d, *J* = 15.5 Hz), 102.84 (d, *J* = 24.3 Hz), 70.40, 68.61, 53.59, 16.63.

##### Preparation of 2-(4-(2-((*tert*-Butyldimethylsilyl) oxy)-3-isothiocyanatopropoxy)phenyl) Acetonitrile (**15a**)

To a solution of TBDMS-Cl (7.5 g, 0.05 mol) in DMF (100 mL) was added imidazole (6.5 g, 0.1 mol). The mixture was stirred for 1 h at rt, then compound **14a** (9 g, 0.04 mol) was added to the reaction mixture and stirred overnight. The reaction was concentrated to dryness, and the desired product was isolated using column chromatography, eluting the desired compound with 80:20 petroleum ether:ethyl acetate. Brown oil (11g, 85% yield). ^1^H NMR (400 MHz, DMSO-*d*_6_) δ 7.27 (d, *J* = 8.6 Hz, 2H), 6.93 (d, *J* = 8.6 Hz, 2H), 4.08 (m, 1H), 4.04–3.90 (m, 3H), 3.83 (dd, *J* = 10.1, 7.0 Hz, 1H), 3.34–3.18 (m, 2H), 0.89 (s, 9H), 0.13 (d, *J* = 13.7 Hz, 6H). ^13^C NMR (101 MHz, DMSO-*d*_6_) δ 158.11, 129.83, 129.83, 123.81, 119.94, 115.26, 115.26, 70.80, 69.89, 54.09, 26.07, 26.07, 26.07, 18.22, −4.21, −4.53. To the silyl protected compound (4.2 g, 12 mmol) in THF (20 mL) was added Ph_3_P (6.5 g, 18 mmol) and stirred at rt for 2 h. Then 20% water was added and stirred at rt overnight. The reaction was concentrated to the dryness, and the desired product was isolated using column chromatography with 100% ethyl acetate. Colorless oil (2.1 g, 55% yield). ^1^H NMR (400 MHz, DMSO-*d*_6_) δ 7.26 (d, *J* = 8.6 Hz, 2H), 7.01–6.86 (m, 2H), 4.07 (dd, *J* = 9.8, 3.2 Hz, 1H), 3.98–3.88 (m, 3H), 3.82 (dd, *J* = 9.8, 7.1 Hz, 1H), 3.3 (s, 1H), 2.70–2.58 (m, 2H), 1.57 (s, 1H), 0.87 (s, 9H), 0.08 (d, *J* = 12.9 Hz, 6H). ^13^C NMR (101 MHz, DMSO-*d*_6_) δ 158.50, 129.29, 129.29, 123.42, 120.02, 115.20, 115.20, 73.39, 70.99, 45.61, 26.26, 26.26, 26.26, 21.97, 18.37, −3.97, −4.20. To a solution of this oil (3 g, 9 mmol) in DCM was added Thio-CDI (5 g, 28 mmol) and stirred overnight at rt under inert atmosphere. The reaction was concentrated to the dryness, and the desired product was isolated using column chromatography eluting the desired compound with 80:20 petroleum ether:ethyl acetate. Colorless oil (0.7 g, 63% yield). ^1^H NMR (400 MHz, DMSO-*d*_6_) δ 7.33–7.21 (m, 2H), 7.04–6.92 (m, 2H), 4.29 (qd, *J* = 5.8, 4.0 Hz, 1H), 4.02–3.88 (m, 5H), 3.78 (dd, *J* = 14.7, 5.8 Hz, 1H), 0.89 (s, 9H), 0.13 (d, *J* = 15.1 Hz, 6H). ^13^C NMR (101 MHz, DMSO-*d*_6_) δ 158.02, 129.86, 129.86, 129.59, 123.97, 119.96, 115.33, 115.33, 69.71, 69.60, 48.85, 26.10, 26.10, 26.10, 21.99, 18.18, −4.16, −4.44.

##### Preparation of 2-(4-(2-((*tert*-Butyldimethylsilyl) oxy)-3-isothiocyanatopropoxy)-3-fluorophenyl) Acetonitrile (**13n**)

The title compound was prepared in similar manner as described for **15n** Silyl protection: 2-(4-(3-azido-2-((*tert*-butyldimethylsilyl)oxy)propoxy)phenyl)acetonitrile Colorless oil (0.9 g, 62% yield). ^1^H NMR (400 MHz, DMSO-*d*_6_) δ 7.26–7.18 (m, 2H), 7.12 (ddd, *J* = 8.3, 2.2, 1.0 Hz, 1H), 4.29–4.19 (m, 1H), 4.10–3.99 (m, 2H), 3.97 (s, 2H), 3.55 (dd, *J* = 12.8, 3.6 Hz, 1H), 3.33 (m, 1H), 0.87 (d, *J* = 4.7 Hz, 9H), 0.11 (dd, *J* = 18.7, 6.1 Hz, 6H). ^13^C NMR (101 MHz, DMSO-*d*_6_) δ 151.82 (d, *J* = 245.3 Hz), 146.06 (d, *J* = 11.2 Hz), 124.90 (d, *J* = 4.9 Hz), 119.53, 116.49 (d, *J* = 18.6 Hz), 115.64 (d, *J* = 1.8 Hz), 71.07, 70.72, 54.01, 26.07, 26.04, 26.04, 21.90, 18.19, −4.40, −4.45. 2-(4-(3-Amino-2-((*tert*-butyldimethylsilyl) oxy) propoxy)-3-fluorophenyl) acetonitrile: Colorless oil (0.4 g, 47% yield). ^1^H NMR (400 MHz, DMSO-*d*_6_) δ 7.26–7.09 (m, 3H), 4.14 (td, *J* = 9.2, 3.0 Hz, 1H), 4.03–3.86 (m, 4H), 2.67 (dd, *J* = 5.3, 2.6 Hz, 2H), 0.92–0.77 (m, 9H), 0.14 to −0.02 (m, 6H). ^13^C NMR (101 MHz, DMSO-*d*_6_) δ 151.84 (d, *J* = 243.4 Hz), 146.40 (d, *J* = 10.6 Hz), 124.83 (d, *J* = 3.6 Hz), 124.25 (d, *J* = 6.7 Hz) 119.55, 116.38 (d, *J* = 23.2 Hz), 115.32 (d, *J* = 1.8 Hz), 73.18, 71.99, 45.48, 26.17, 26.17, 26.17, 21.89, 18.29, −4.32, −4.43.

##### 2-(4-(2-((*tert*-Butyldimethylsilyl) oxy)-3-isothiocyanatopropoxy)-3-fluorophenyl) Acetonitrile **(15n**)

Colorless oil (0.2 g, 44% yield). ^1^H NMR (400 MHz, DMSO-*d*_6_) δ 7.29–7.19 (m, 2H), 7.13 (ddd, *J* = 8.4, 2.1, 0.9 Hz, 1H), 4.32 (tt, *J* = 6.1, 4.1 Hz, 1H), 4.15–4.01 (m, 2H), 3.93 (dd, *J* = 14.8, 3.8 Hz, 1H), 3.77 (dd, *J* = 14.7, 5.7 Hz, 1H), 0.88 (s, 9H), 0.17–0.07 (m, 6H). ^13^C NMR (101 MHz, DMSO-*d*_6_) δ 151.81 (d, *J* = 246.3 Hz), 145.96 (d, *J* = 11.5 Hz), 129.78, 125.03 (d, J = 3.7 Hz) 124.92 (d, *J* = 6.8 Hz), 119.55, 116.47 (d, *J* = 18.1 Hz), 115.81(d, *J* = 1.5 Hz), 70.83, 69.55, 48.76, 26.05, 26.05, 26.05, 21.90, 18.14, −4.57, −4.57.

##### Preparation of 2-(4-(2-((*tert*-Butyldimethylsilyl) oxy)-3-isothiocyanatopropoxy)-2-fluorophenyl) Acetonitrile (**15o**)

The title compound was prepared in similar manner as described for **15a**. 2-(4-(3-Azido-2-((*tert*-butyldimethylsilyl) oxy)propoxy)-2-fluorophenyl) acetonitrile: Colorless oil (0.8 g, 54% yield). ^1^H NMR (400 MHz, DMSO-*d*_6_) δ 7.37 (t, *J* = 8.8 Hz, 1H), 6.95–6.79 (m, 2H), 4.20 (ddt, *J* = 7.9, 5.7, 2.7 Hz, 1H), 4.03 (dd, *J* = 10.0, 4.4 Hz, 1H), 3.96 (d, *J* = 8.0 Hz, 3H), 3.53 (dd, *J* = 12.9, 3.6 Hz, 1H), 3.43–3.23 (m, 1H), 0.88 (s, 9H), 0.12 (d, *J* = 14.0 Hz, 6H). ^13^C NMR (101 MHz, DMSO-*d*_6_) δ 162.22 (d, *J* = 230.6 Hz), 159.93 (d, *J* = 4.6 Hz), 131.38 (d, *J* = 5.3 Hz), 118.77, 111.63 (d, *J* = 3.2 Hz), 110.86 (d, *J* = 17.7 Hz), 102.65 (d, *J* = 24.8 Hz), 70.65, 70.41, 54.00, 26.06, 26.06, 26.06, 18.21, 16.63, −4.25, −4.53. 2-(4-(3-Amino-2-((*tert*-butyldimethylsilyl) oxy) propoxy)-2-fluorophenyl) acetonitrile: Colorless oil (0.6 g, 71% yield). ^1^H NMR (400 MHz, DMSO-*d*_6_) δ 7.36 (td, *J* = 8.8, 6.2 Hz, 1H), 6.94–6.77 (m, 2H), 4.09 (td, *J* = 10.4, 9.9, 3.4 Hz, 2H), 4.02–3.90 (m, 3H), 3.90–3.79 (m, 1H), 2.69–2.59 (m, 2H), 0.90–0.83 (m, 9H), 0.08 (d, *J* = 13.3 Hz, 6H). ^13^C NMR (101 MHz, DMSO-*d*_6_) δ 162.22 (d, *J* = 230.6 Hz), 159.93 (d, *J* = 4.6 Hz), 131.38 (d, *J* = 5.3 Hz), 118.77, 111.63 (d, *J* = 3.2 Hz), 110.86 (d, *J* = 17.7 Hz), 102.65 (d, *J* = 24.8 Hz), 70.65, 70.41, 54.00, 26.06, 18.21, 16.63, −4.25, −4.53. 2-(4-(2-((*tert*-Butyldimethylsilyl) oxy)-3-isothiocyanatopropoxy)-2-fluorophenyl) acetonitrile (**15o**): Colorless oil (0.4 g, 44% yield). ^1^H NMR (400 MHz, DMSO-*d*_6_) δ 7.37 (m, 1H), 6.93 (m, 1H), 6.84 (m, 1H), 4.34–4.19 (m, 1H), 4.12–4.00 (m, 1H), 4.00–3.89 (m, 3H), 3.83–3.66 (m, 2H), 0.88 (d, *J* = 4.7 Hz, 9H), 0.17–0.05 (m, 6H). ^13^C NMR (101 MHz, DMSO-*d*_6_) δ 161.07 (d, *J* = 228.5 Hz), 159.79 (d, *J* = 6.6 Hz), 131.46 (d, *J* = 5.3 Hz), 129.64, 118.78, 111.65 (d, *J* = 4.6 Hz), 110.74 (d, *J* = 17.2 Hz), 102.96 (d, *J* = 23.9 Hz), 71.02, 70.44, 69.45, 48.77, 47.04, 26.11, 18.22, −4.18, −4.36.

#### General Procedure 1 for Preparation of (**6a**–**6u**)

To a solution of **17a**–**17t** (1 equiv) in EtOH (20 mL) was added the corresponding isothiocyanate **15a**, **15n**, **15o**, and **15v** (1 equiv) and stirred overnight at 70 °C. The reaction was concentrated and then dissolved in DMF (20 mL). DIC (1.2 equiv) and Et_3_N (2 equiv) were added to the mixture and stirred overnight at 80 °C. The reaction was concentrated to dryness and the desired product was isolated using column chromatography eluting the desired compound with 50:50 petroleum ether:ethyl acetate. The product was then dissolved in MeOH (50 mL) with 20% TFA and stirred for overnight at rt. Upon completion, the mixture was neutralized using Et_3_N and concentrated and extracted with mixture of EtOAc and water. The organic layer was concentrated and purified using column chromatography eluting the desired compound with 20:80 petroleum ether:ethyl acetate.

##### 2-(4-(3-((6-Chloro-1-methyl-1*H*-benzo[*d*]imidazol-2-yl)amino)-2-hydroxypropoxy)phenyl) Acetonitrile (**6a**)

The title compound was prepared according to general procedure 1. ^1^H NMR (400 MHz, DMSO-*d*_6_) δ 7.31–7.21 (m, 3H), 7.15 (d, *J* = 8.4 Hz, 1H), 6.95 (ddt, *J* = 8.3, 5.6, 2.5 Hz, 4H), 4.13 (m, 1H), 4.08–3.96 (m, 2H), 3.94 (s, 2H), 3.64–3.56 (m, 2H), 3.55 (s, 3H). ^13^C NMR (101 MHz, DMSO-*d*_6_) δ 159.4, 155.1, 132.3, 129.9, 128.3, 126.7, 126.7, 122.7, 120.8, 115.7, 113.3, 107.7, 69.6, 68.1, 46.4, 29.2, 21.9. LCMS *m*/*z* calcd for C_19_H_20_ClN_4_O_2_ + [M + H]^+^: 371.1, found 371.1 with *t*_R_ 2.15 min. HRMS *m*/*z* calcd for C_19_H_20_ClN_4_O_2_ + [M + H]^+^: 371.1270, found 371.1279 with *t*_R_ 2.15 min.

##### 2-(4-(3-((5-Chloro-1-methyl-1*H*-benzo[*d*]imidazol-2-yl)amino)-2-hydroxypropoxy)phenyl) Acetonitrile (**6b**)

The title compound was prepared according to general procedure 1. ^1^H NMR (400 MHz, DMSO-*d*_6_) δ 7.71 (s, 1H), 7.33–7.23 (m, 4H), 7.07 (dd, *J* = 8.4, 2.0 Hz, 1H), 7.01–6.93 (m, 2H), 4.13 (m, 1H), 4.08–3.96 (m, 2H), 3.94 (s, 2H), 3.64–3.56 (m, 2H), 3.55 (s, 3H). ^13^C NMR (101 MHz, DMSO-*d*_6_) δ 158.52, 155.07, 133.39, 129.75, 129.75, 128.50, 126.88, 126.03, 123.56, 120.02, 115.43, 115.43, 113.73, 109.71, 70.63, 68.19, 46.46, 29.25, 21.98. LCMS *m*/*z* calcd for C_19_H_20_ClN_4_O_2_^+^ [M + H]^+^: 371.1, found 371.1 with *t*_R_ 2.12 min. HRMS *m*/*z* calcd for C_19_H_20_ClN_4_O_2_^+^ [M + H]^+^: 371.1270, found 371.1273.

##### 2-(4-(3-((5,6-Dichloro-1-methyl-1*H*-benzo[*d*]imidazol-2-yl) amino)-2-hydroxypropoxy) phenyl) Acetonitrile (**6c**)

The title compound was prepared according to general procedure 1. ^1^H NMR (400 MHz, DMSO-*d*_6_) δ 7.47 (s, 1H), 7.36 (s, 1H), 7.26 (d, *J* = 8.7 Hz, 2H), 7.10 (t, *J* = 5.7 Hz, 1H), 6.97 (d, *J* = 8.7 Hz, 2H), 5.44 (d, *J* = 4.9 Hz, 1H), 4.13 (h, *J* = 5.5 Hz, 1H), 4.02 (dd, *J* = 10.0, 4.3 Hz, 1H), 3.95 (d, *J* = 10.2 Hz, 3H), 3.59–3.40 (m, 5H). ^13^C NMR (101 MHz, DMSO-*d*_6_) δ 158.59, 157.45, 142.90, 135.76, 129.74, 130.10, 123.50, 122.74, 120.48, 120.02, 115.96, 115.66, 115.44, 109.27, 70.89, 68.17, 46.34, 29.11, 21.98. LCMS *m*/*z* calcd for C_19_H_19_Cl_2_N_4_O_2_^+^ [M + H]^+^: 405.0, found 404.7 with t_R_ 2.25 min. HRMS *m*/*z* calcd for C_19_H_19_Cl_2_N_4_O_2_^+^ [M + H]^+^: 405.0880, found 405.0886.

##### 2-(4-(2-Hydroxy-3-((1-ethyl-1*H*-benzo [*d*]imidazol-2-yl)amino)propoxy)phenyl) Acetonitrile (**6e**)

The title compound was prepared according to general procedure 1. ^1^H NMR (400 MHz, DMSO-*d*_6_) δ 7.31–7.21 (m, 3H), 7.15 (d, *J* = 8.4 Hz, 1H), 6.95 (ddt, *J* = 8.3, 5.6, 2.5 Hz, 4H), 5.55 (d, *J* = 4.9 Hz, 1H), 4.16–3.88 (m, 4H), 3.93 (s, 2H), 3.59–3.39 (m, 2H), 1.17 (t, *J* = 7.1 Hz, 3H). ^13^C NMR (101 MHz, DMSO) δ 158.12, 155.25, 141.26, 135.15, 129.26, 123.01, 122.56, 120.08, 119.54, 115.63, 114.94, 107.45, 70.44, 67.92, 45.88, 36.21, 21.49, 13.81. HRMS *m*/*z* calcd for C_20_H_22_ClN_4_O_2_^+^ [M + H]^+^: 385.1426, found 385.1431.

##### 2-(4-(3-((6-Chloro-1-isopropyl-1*H*-benzo[*d*]imidazol-2-yl)amino)-2-hydroxypropoxy)phenyl) Acetonitrile (**6f**)

The title compound was prepared according to general procedure 1. ^1^H NMR (400 MHz, DMSO-*d*_6_) δ 7.38 (d, *J* = 2.0 Hz, 1H), 7.29–7.22 (m, 2H), 7.17 (d, *J* = 8.3 Hz, 1H), 6.96 (dd, *J* = 8.7, 2.4 Hz, 3H), 6.80 (t, *J* = 5.6 Hz, 1H), 5.56 (d, *J* = 4.9 Hz, 1H), 4.63 (hept, *J* = 6.8 Hz, 1H), 4.12 (p, *J* = 5.4 Hz, 1H), 4.01 (dd, *J* = 10.0, 4.3 Hz, 1H), 3.94 (d, *J* = 6.3 Hz, 3H), 3.62–3.40 (m, 2H), 1.47 (d, *J* = 6.8 Hz, 6H). ^13^C NMR (101 MHz, DMSO) δ 158.62, 155.52, 142.11, 134.19, 129.75, 123.49, 122.80, 120.48, 120.03, 116.40, 115.44, 109.81, 70.99, 68.38, 46.68, 45.88, 21.99, 20.67. LCMS *m*/*z* calcd for C_21_H_24_ClN_4_O_2_^+^ [M + H]^+^: 399.2, found 399.1 with *t*_R_ 2.23 min. HRMS *m*/*z* calcd for C_21_H_24_ClN_4_O_2_^+^ [M + H]^+^: 399.1583, found 399.1591.

##### 2-(4-(3-((1-(*tert*-Butyl)-6-chloro-1*H*-benzo[*d*]imidazol-2-yl)amino)-2-hydroxypropoxy)phenyl) Acetonitrile (**6g**)

The title compound was prepared according to general procedure 1. ^1^H NMR (400 MHz, DMSO-*d*_6_) δ 7.50 (d, *J* = 1.9 Hz, 1H), 7.28–7.23 (m, 2H), 7.18 (d, *J* = 8.4 Hz, 1H), 6.99–6.94 (m, 3H), 6.03 (t, *J* = 5.4 Hz, 1H), 5.63 (s, 1H), 4.20–4.12 (m, 1H), 4.01 (dd, *J* = 10.0, 4.6 Hz, 1H), 3.95 (d, *J* = 9.0 Hz, 3H), 3.66–3.43 (m, 2H), 1.75 (s, 9H). ^13^C NMR (101 MHz, DMSO-*d*_6_) δ 158.59, 156.22, 141.72, 135.47, 129.84, 129.76, 123.52, 122.74, 120.53, 120.02, 116.55, 115.47, 115.42, 112.49, 71.26, 68.14, 58.31, 47.32, 30.02, 21.98. LCMS *m*/*z* calcd for C_22_H_26_ClN_4_O_2_^+^ [M + H]^+^: 413.1, found 412.8 with *t*_R_ 2.36 min. HRMS *m*/*z* calcd for C_22_H_26_ClN_4_O_2_^+^ [M + H]^+^: 413.1739, found 413.1746.

##### 2-(4-(3-((6-Chloro-1-neopentyl-1*H*-benzo[*d*]imidazol-2-yl)amino)-2-hydroxypropoxy)phenyl) Acetonitrile (**6h**)

The title compound was prepared according to general procedure 1. ^1^H NMR (400 MHz, DMSO-*d*_6_) δ 7.30 (d, *J* = 2.0 Hz, 1H), 7.28–7.20 (m, 2H), 7.16 (d, *J* = 8.3 Hz, 1H), 6.94 (dd, *J* = 8.5, 2.1 Hz, 3H), 6.71 (t, *J* = 5.7 Hz, 1H), 5.61 (s, 1H), 4.14 (m, 1H), 4.00 (dd, *J* = 10.0, 4.4 Hz, 1H), 3.92 (dd, *J* = 9.8, 6.1 Hz, 3H), 3.84 (s, 2H), 3.51 (m, 2H), 0.95 (s, 9H). ^13^C NMR (101 MHz, DMSO-*d*_6_) δ 158.60, 156.89, 141.44, 137.24, 129.74, 129.74, 123.49, 122.83, 120.55, 120.02, 115.99, 115.39, 115.39, 109.38, 70.92, 68.49, 52.17, 46.43, 35.35, 28.24, 28.24, 24.57, 21.98. LCMS *m*/*z* calcd for C_23_H_28_ClN_4_O_2_^+^ [M + H]^+^: 427.1, found 427.2 with *t*_R_ 2.46 min. HRMS *m*/*z* calcd for C_23_H_28_ClN_4_O_2_^+^ [M + H]^+^: 427.1896, found 427.1894.

##### 2-(4-(3-((6-Chloro-1-cyclopropyl-1*H*-benzo[*d*]imidazol-2-yl)amino)-2-hydroxypropoxy)phenyl) Acetonitrile (**6i**)

The title compound was prepared according to general procedure 1. ^1^H NMR (400 MHz, DMSO-*d*_6_) δ 7.29–7.23 (m, 2H), 7.22–7.11 (m, 2H), 7.00–6.94 (m, 3H), 6.57 (t, *J* = 5.7 Hz, 1H), 5.55 (d, *J* = 4.9 Hz, 1H), 4.15 (q, *J* = 5.2, 4.7 Hz, 1H), 4.07–4.00 (m, 1H), 4.00–3.90 (m, 3H), 3.63–3.42 (m, 2H), 3.00 (tt, *J* = 7.0, 3.7 Hz, 1H), 1.21–1.10 (m, 2H), 0.97–0.80 (m, 2H). ^13^C NMR (101 MHz, DMSO-*d*_6_) δ 158.59, 156.95, 141.44, 136.54, 129.75, 123.52, 123.00, 120.81, 120.03, 116.30, 115.45, 108.57, 71.06, 68.23, 46.44, 22.99, 21.98, 7.07, 7.03. LCMS *m*/*z* calcd for C_21_H_22_ClN_4_O_2_^+^ [M + H]^+^: 397.1, found 386.8 with 2.21 *t*_R_ min.

##### 2-(4-(3-((6-Chloro-1-cyclobutyl-1*H*-benzo[*d*]imidazol-2-yl) amino)-2-hydroxypropoxy)phenyl) Acetonitrile (**6j**)

The title compound was prepared according to general procedure 1. ^1^H NMR (400 MHz, DMSO-*d*_6_) δ 7.46 (d, *J* = 2.1 Hz, 1H), 7.30–7.13 (m, 3H), 7.03–6.91 (m, 3H), 6.70 (t, *J* = 5.6 Hz, 1H), 5.52 (d, *J* = 4.9 Hz, 1H), 4.88 (m, 1H), 4.13 (m, 1H), 4.01 (dd, *J* = 10.0, 4.4 Hz, 1H), 3.97–3.90 (m, 3H), 3.54 (dt, *J* = 13.4, 5.6 Hz, 1H), 3.43 (ddd, *J* = 13.4, 6.8, 5.2 Hz, 1H), 2.79–2.66 (m, 2H), 2.38 (m, 2.2 Hz, 2H), 1.94 (m, 1H), 1.76 (qt, *J* = 10.5, 8.2 Hz, 1H). ^13^C NMR (101 MHz, DMSO-*d*_6_) δ 158.60, 155.79, 142.06, 134.87, 129.74, 129.74, 123.50, 122.94, 120.76, 120.02, 116.58, 115.44, 115.44, 109.66, 70.98, 68.29, 47.88, 46.63, 28.38, 28.38, 21.99, 14.91. LCMS *m*/*z* calcd for C_22_H_24_ClN_4_O_2_^+^ [M + H]^+^: 411.1, found 410.8.2 with *t*_R_ 2.39 min. LCMS *m*/*z* calcd for C_22_H_24_ClN_4_O_2_^+^ [M + H]^+^: 411.1583, found 411.1583.

##### 2-(4-(3-((6-Chloro-1-cyclopentyl-1*H*-benzo[*d*]imidazol-2-yl)amino)-2-hydroxypropoxy)phenyl) Acetonitrile (**6k**)

The title compound was prepared according to general procedure 1. ^1^H NMR (400 MHz, DMSO-*d*_6_) δ 7.29–7.24 (m, 2H), 7.21–7.17 (m, 2H), 6.99–6.93 (m, 3H), 6.84 (t, *J* = 5.6 Hz, 1H), 5.53 (d, *J* = 5.1 Hz, 1H), 4.76 (p, *J* = 8.6 Hz, 1H), 4.12 (dd, *J* = 8.2, 3.5 Hz, 1H), 4.08–3.98 (m, 1H), 3.95 (d, *J* = 9.5 Hz, 3H), 3.58–3.38 (m, 2H), 2.06–1.88 (m, 6H), 1.68 (s, 2H). ^13^C NMR (101 MHz, DMSO-*d*_6_) δ 158.60, 156.14, 142.23, 133.78, 129.75, 129.75, 123.50, 122.77, 120.61, 120.02, 116.60, 115.39, 115.39, 109.41, 70.97, 68.34, 54.52, 46.67, 28.92, 28.92, 24.73, 24.73, 21.98. LCMS *m*/*z* calcd for C_23_H_26_ClN_4_O_2_^+^ [M + H]^+^: 425.1, found 424.6 with *t*_R_ 2.33 min. HRMS *m*/*z* calcd for C_23_H_26_ClN_4_O_2_^+^ [M + H]^+^: 425.1739, found 425.1757.

##### 2-(4-(3-((6-Chloro-1-cyclohexyl-1*H*-benzo[*d*]imidazol-2-yl)amino)-2-hydroxypropoxy)phenyl) Acetonitrile (**6l**)

The title compound was prepared according to general procedure 1. ^1^H NMR (400 MHz, DMSO-*d*_6_) δ 7.41 (d, *J* = 2.0 Hz, 1H), 7.32 (d, *J* = 5.0 Hz, 1H), 7.28–7.22 (m, 1H), 7.17 (d, *J* = 8.3 Hz, 1H), 6.96 (dd, *J* = 8.6, 2.0 Hz, 3H), 6.88 (t, *J* = 5.8 Hz, 1H), 5.60 (s, 1H), 4.49 (d, *J* = 5.6 Hz, 1H), 4.27–4.10 (m, 2H), 4.01 (dd, *J* = 10.1, 4.4 Hz, 1H), 3.94 (d, *J* = 13.7 Hz, 3H), 3.56 (dd, *J* = 9.5, 4.1 Hz, 1H), 2.16–2.00 (m, 2H), 1.84 (d, *J* = 10.9 Hz, 2H), 1.69 (dd, *J* = 27.8, 9.9 Hz, 3H), 1.51–1.34 (m, 3H). ^13^C NMR (101 MHz, DMSO-*d*_6_) δ 158.58, 155.56, 141.86, 134.37, 129.75, 129.75, 123.48, 122.87, 120.50, 120.02, 116.36, 115.45, 115.45, 110.14, 70.98, 68.39, 63.36, 53.71, 46.69, 30.31, 25.91, 24.84, 24.56, 21.97. LCMS *m*/*z* calcd for C_24_H_28_ClN_4_O_2_^+^ [M + H]^+^: 439.1, found 439.7 with *t*_R_ 2.40 min. HRMS *m*/*z* calcd for C_24_H_28_ClN_4_O_2_^+^ [M + H]^+^: 439.1896, found 439.1905.

##### 2-(4-(3-((1-(2-(Benzyloxy)ethyl)-6-chloro-1*H*-benzo[*d*]imidazol-2-yl)amino)-2-hydroxypropoxy)phenyl) Acetonitrile (**6m**)

The title compound was prepared according to general procedure 1. ^1^H NMR (400 MHz, DMSO-*d*_6_) δ 7.32 (d, *J* = 2.0 Hz, 1H), 7.26 (ddt, *J* = 11.2, 6.8, 2.5 Hz, 5H), 7.20–7.14 (m, 3H), 6.95 (td, *J* = 8.1, 7.4, 2.1 Hz, 3H), 6.87 (t, *J* = 5.7 Hz, 1H), 5.56 (s, 1H), 4.45 (s, 2H), 4.24 (t, *J* = 5.2 Hz, 2H), 4.11 (p, *J* = 5.8 Hz, 1H), 3.99 (dd, *J* = 9.9, 4.4 Hz, 1H), 3.95 (s, 2H), 3.92–3.89 (m, 1H), 3.68 (t, *J* = 5.1 Hz, 2H), 3.61–3.39 (m, 2H). ^13^C NMR (101 MHz, DMSO-*d*_6_) δ 158.59, 156.26, 141.53, 138.53, 136.49, 129.73, 129.73, 128.62, 128.62, 127.80, 127.50, 127.50, 123.47, 123.08, 120.64, 120.02, 116.09, 115.41, 115.41, 108.75, 72.43, 70.81, 68.52, 68.36, 46.36, 42.27, 21.98. LCMS *m*/*z* calcd for C_27_H_27_ClN_4_O_3_^+^ [M + H]^+^: 491.1, found 490.6 with *t*_R_ 2.37 min.

##### 2-(4-(3-((6-Chloro-1-isopropyl-1*H*-benzo[*d*]imidazol-2-yl)amino)-2-hydroxypropoxy)-3-fluorophenyl) Acetonitrile (**6n**)

The title compound was prepared according to general procedure 1. ^1^H NMR (400 MHz, DMSO-*d*_6_) δ 7.38 (d, *J* = 2.0 Hz, 1H), 7.25–7.15 (m, 3H), 7.11 (dd, *J* = 8.6, 2.0 Hz, 1H), 6.96 (dd, *J* = 8.4, 2.0 Hz, 1H), 6.82 (t, *J* = 5.6 Hz, 1H), 5.62 (d, *J* = 4.4 Hz, 1H), 4.63 (hept, *J* = 6.9 Hz, 1H), 4.13–3.99 (m, 2H), 3.99 (s, 2H), 3.60–3.41 (m, 2H), 1.47 (d, *J* = 6.8 Hz, 6H). ^13^C NMR (101 MHz, DMSO-*d*_6_) δ 155.50, 151.94 (d, *J* = 244.5 Hz), 146.53 (d, *J* = 11.2 Hz), 142.07, 134.17, 124.92 (d, *J* = 3.9 Hz), 124.43 (d, *J* = 6.5 Hz), 122.81, 120.48, 119.61, 116.40 (d, *J* = 5.8 Hz), 115.88 (d, *J* = 1.8 Hz), 109.82, 71.99, 68.31, 46.55, 45.88, 21.89, 20.66, 20.66. LCMS *m*/*z* calcd for C_21_H_24_ClFN_4_O_2_^+^ [M + H]^+^: 417.15, found 416.6 with *t*_R_ 2.26 min. HRMS *m*/*z* calcd for C_21_H_24_ClFN_4_O_2_^+^ [M + H]^+^: 417.1489, found 417.1500.

##### 2-(4-(3-((6-Chloro-1-isopropyl-1*H*-benzo[*d*]imidazol-2-yl)amino)-2-hydroxypropoxy)-2-fluorophenyl) Acetonitrile (**6o**)

The title compound was prepared according to general procedure 1. ^1^H NMR (400 MHz, DMSO-*d*_6_) δ 7.38 (d, *J* = 2.0 Hz, 1H), 7.35 (t, *J* = 8.8 Hz, 1H), 7.17 (d, *J* = 8.4 Hz, 1H), 6.99–6.88 (m, 2H), 6.87–6.81 (m, 1H), 6.79 (d, *J* = 5.6 Hz, 1H), 5.57 (s, 1H), 4.63 (m, 1H), 4.14 (t, *J* = 5.7 Hz, 1H), 4.05 (dd, *J* = 10.1, 4.2 Hz, 1H), 4.02 (m, 1H) 3.90 (S, 2H), 3.59–3.39 (m, 2H), 1.47 (d, *J* = 6.9 Hz, 6H). ^13^C NMR (101 MHz, DMSO-*d*_6_) δ 160.98 (d, *J* = 244.8 Hz), 160.42 (d, *J* = 10.9 Hz), 155.46, 142.07, 134.17, 131.32 (d, *J* = 5.3 Hz), 122.81, 120.48, 118.85, 116.39, 111.77 (d, *J* = 3.1 Hz), 110.49 (d, *J* = 16.2 Hz), 109.82, 102.85 (d, *J* = 24.9 Hz), 71.47, 68.18, 46.54, 45.88, 20.66, 20.66, 16.65. LCMS *m*/*z* calcd for C_21_H_24_ClFN_4_O_2_^+^ [M + H]^+^: 417.15, found 416.5 with *t*_R_ 2.24 min. HRMS *m*/*z* calcd for C_21_H_24_ClFN_4_O_2_^+^ [M + H]^+^: 417.1489, found 417.1483.

##### 2-(4-(2-Hydroxy-3-((1-isopropyl-1*H*-benzo[*d*]imidazol-2-yl)amino)propoxy)phenyl) Acetonitrile (**6p**)

The title compound was prepared according to general procedure 1. ^1^H NMR (400 MHz, DMSO-*d*_6_) δ 7.34 (d, *J* = 7.7 Hz, 1H), 7.29–7.23 (m, 2H), 7.20 (d, *J* = 7.7 Hz, 1H), 7.00–6.92 (m, 3H), 6.88 (t, *J* = 7.6, 1.3 Hz, 1H), 6.70 (t, *J* = 5.6 Hz, 1H), 5.78 (s, 1H), 4.63 (p, *J* = 6.8 Hz, 1H), 4.12 (d, *J* = 6.3 Hz, 1H), 4.01 (dd, *J* = 9.9, 4.5 Hz, 1H), 3.95 (d, *J* = 7.9 Hz, 3H), 3.59–3.39 (m, 2H), 1.49 (d, *J* = 6.8 Hz, 6H). ^13^C NMR (101 MHz, DMSO-*d*_6_) δ 158.62, 154.78, 142.96, 133.26, 129.75, 123.49, 120.55, 120.03, 118.82, 115.68, 115.44, 110.18, 70.95, 68.77, 46.77, 45.69, 21.98, 20.84. LCMS *m*/*z* calcd for C_21_H_26_N_4_O_2_^+^ [M + H]^+^: 365.2, found 364.6 with 2.05 *t*_R_ min.

##### 2-(4-(3-((6-Chloro-1-(2-(dimethylamino)ethyl)-1*H*-benzo[*d*]imidazol-2-yl)amino)-2-hydroxypropoxy)phenyl) Acetonitrile (**6q**)

The title compound was prepared according to general procedure 1. ^1^H NMR (400 MHz, DMSO-*d*_6_) δ 7.26 (d, *J* = 8.5 Hz, 2H), 7.14 (ddd, *J* = 8.4, 7.3, 1.5 Hz, 1H), 6.98 (ddd, *J* = 13.9, 7.8, 1.5 Hz, 1H), 6.91–6.84 (m, 3H), 6.64 (dtd, *J* = 9.1, 7.6, 1.5 Hz, 1H), 5.18 (t, *J* = 4.4 Hz, 1H), 4.63–4.49 (m, 1H), 3.94 (s, 2H), 3.89–3.72 (m, 2H), 3.67 (dq, *J* = 13.1, 5.3 Hz, 1H), 3.60–3.44 (m, 1H), 3.27–3.03 (m, 2H), 2.76 (s, 6H). ^13^C NMR (101 MHz, DMSO-*d*_6_) δ 158.40, 145.19, 130.26, 129.74, 129.74, 129.42, 123.58, 120.04, 119.30, 117.69, 117.20, 116.31, 115.41, 115.41, 70.95, 67.28, 60.23, 48.96, 43.64, 21.98, 21.24, 14.56. LCMS *m*/*z* calcd for C_22_H_27_ClN_5_O_2_^+^ [M + H]^+^: 428.1, found 427.9 with *t*_R_ 2.05 min. HRMS *m*/*z* calcd for C_22_H_27_ClN_5_O_2_^+^ [M + H]^+^: 428.1848, found 428.2126.

##### 2-(4-(3-((6-Chloro-1-(1-methylpiperidin-4-yl)-1*H*-benzo[*d*]imidazol-2-yl)amino)-2-hydroxypropoxy)phenyl) Acetonitrile (**6r**)

The title compound was prepared according to general procedure 1. ^1^H NMR (400 MHz, DMSO-*d*_6_) δ 7.26 (d, *J* = 8.6 Hz, 2H), 7.12–7.06 (m, 1H), 7.02 (d, *J* = 7.7 Hz, 1H), 6.94–6.90 (m, 2H), 6.72 (d, *J* = 8.1 Hz, 1H), 6.59 (td, *J* = 7.5, 1.3 Hz, 1H), 5.27 (s, 1H), 4.35 (d, *J* = 7.9 Hz, 1H), 4.07–3.98 (m, 2H), 3.93 (d, *J* = 9.5 Hz, 3H), 3.90–3.81 (m, 1H), 3.71 (s, 1H), 3.53 (dt, *J* = 12.7, 5.7 Hz, 1H), 2.67 (d, *J* = 11.5 Hz, 2H), 2.16 (s, 3H), 1.85 (d, *J* = 12.5 Hz, 2H), 1.37 (q, *J* = 10.1 Hz, 2H). ^13^C NMR (101 MHz, DMSO-*d*_6_) δ 158.46, 143.74, 129.74, 129.74, 128.84, 128.32, 126.89, 123.57, 120.01, 116.49, 115.40, 115.40, 112.50, 71.07, 67.85, 60.23, 54.33, 48.92, 47.98, 46.36, 32.06, 31.17, 21.99. LCMS *m*/*z* calcd for C_24_H_29_ClN_5_O_2_^+^ [M + H]^+^: 454.2, found 454.2 with *t*_R_ 2.08 min. HRMS *m*/*z* calcd for C_24_H_29_ClN_5_O_2_^+^ [M + H]^+^: 454.2005, found 454.2270.

##### 2-(4-(3-((6-Chloro-1-(oxetan-3-yl)-1*H*-benzo[*d*]imidazol-2-yl)amino)-2-hydroxypropoxy)phenyl) Acetonitrile (**6s**)

The title compound was prepared according to general procedure 1. ^1^H NMR (400 MHz, DMSO-*d*_6_) δ 7.67 (d, *J* = 2.0 Hz, 1H), 7.26 (dd, *J* = 8.5, 1.9 Hz, 3H), 7.06 (dd, *J* = 8.4, 2.1 Hz, 1H), 6.97 (d, *J* = 8.6 Hz, 2H), 6.86 (t, *J* = 5.6 Hz, 1H), 5.61 (tt, *J* = 7.6, 5.4 Hz, 1H), 5.46 (s, 1H), 5.11–4.90 (m, 4H), 4.12 (t, *J* = 5.7 Hz, 1H), 4.01 (dd, *J* = 10.0, 4.6 Hz, 1H), 3.98–3.90 (m, 3H), 3.59–3.35 (m, 2H). ^13^C NMR (101 MHz, DMSO-*d*_6_) δ 158.57, 155.77, 142.25, 133.86, 129.74, 123.52, 123.45, 121.32, 120.03, 116.92, 115.45, 109.20, 75.18, 70.86, 68.09, 48.58, 46.60, 21.98. LCMS *m*/*z* calcd for C_21_H_22_ClN_4_O_3_^+^ [M + H]^+^: 413.1 found 412.5 with *t*_R_ 2.17 min.

##### 2-(4-(3-((6-Chloro-1-(2-methoxyethyl)-1*H*-benzo[*d*]imidazol-2-yl)amino)-2-hydroxypropoxy)phenyl) Acetonitrile (**6t**)

The title compound was prepared according to general procedure 1. ^1^H NMR (400 MHz, DMSO-*d*_6_) δ 7.29 (d, *J* = 2.1 Hz, 1H), 7.27 (d, *J* = 1.9 Hz, 1H), 7.25 (s, 1H), 7.16 (d, *J* = 8.3 Hz, 1H), 7.00–6.93 (m, 3H), 6.81 (t, *J* = 5.7 Hz, 1H), 5.50 (d, *J* = 22.6 Hz, 2H), 4.20–4.07 (m, 3H), 3.95 (d, *J* = 8.9 Hz, 3H), 3.68–3.39 (m, 5H), 3.20 (s, 3H). ^13^C NMR (101 MHz, DMSO-*d*_6_) δ 158.60, 156.26, 141.55, 136.43, 129.74, 129.74, 123.50, 123.06, 120.63, 120.02, 116.13, 115.43, 115.43, 108.48, 70.83, 68.24, 58.82, 46.31, 42.18, 23.77, 21.98. LCMS *m*/*z* calcd for C_21_H_24_ClN_4_O_3_^+^ [M + H]^+^: 415.1, found 414.8 with *t*_R_ 2.21 min. LCMS *m*/*z* calcd for C_21_H_24_ClN_4_O_3_^+^ [M + H]^+^: 415.1532, found 415.1547.

##### 2-(4-(3-((6-Chloro-1-(2-hydroxyethyl)-1*H*-benzo[*d*]imidazol-2-yl)amino)-2-hydroxypropoxy)phenyl) Acetonitrile (**6u**)

The title compound was prepared according to general procedure 1. ^1^H NMR (400 MHz, DMSO-*d*_6_) δ 7.31–7.22 (m, 3H), 7.17 (d, *J* = 8.3 Hz, 1H), 7.00–6.93 (m, 3H), 6.81 (t, *J* = 5.7 Hz, 1H), 5.55 (s, 1H), 5.01 (t, *J* = 5.1 Hz, 1H), 4.11 (q, *J* = 5.6 Hz, 1H), 4.03 (dt, *J* = 14.2, 4.9 Hz, 3H), 3.95 (d, *J* = 9.7 Hz, 3H), 3.66 (q, *J* = 5.2 Hz, 2H), 3.59–3.41 (m, 2H). ^13^C NMR (101 MHz, DMSO-*d*_6_) δ 158.59, 156.45, 141.57, 136.68, 129.74, 123.48, 123.00, 120.49, 120.03, 116.08, 115.44, 108.52, 70.84, 68.36, 60.10, 46.39, 44.84, 21.99. LCMS *m*/*z* calcd for C_20_H_22_ClN_4_O_3_^+^ [M + H]^+^: 401.1, found 401.8 with *t*_R_ 2.08 min.

##### Preparation of 2-(4-(2-Amino-3-((6-chloro-1*H*-benzo[*d*]imidazol-2-yl)amino)propoxy)phenyl) Acetonitrile (**18**)

To a stirred solution of **6f** (100 mg, 0.25 mmol) in anhydrous THF (15 mL) Ph_3_P (99 mg, 0.38 mmol) was added under anhydrous condition at rt. After 5 min the mixture was cooled over an ice bath before addition of DIAD (76 mg, 0.38 mmol) followed by diphenyl phosphoryl azide (104 mg, 0.38 mmol). The mixture was stirred for 5 min in ice bath, then another 5 min at rt and was then allowed to stir at 45 °C overnight. The mixture was purified using column chromatography eluting the desired compound with 40:60 ethyl acetate:petroleum ether. Yellow solid (64 mg, 62%). The resulting solid (60 mg, 0.15 mmol) was then added to a solution of Ph_3_P (78 mg, 0.3 mmol) in 10 mL THF and stirred for 2 h at 65 °C. Then 2 mL of water with 0.5 mL of ammonium hydroxide was added and stirred overnight at rt. The mixture was purified using column chromatography eluting the desired compound with 90:10 ethyl acetate:MeOH containing 1% NH_3_ (0.7 N). White solid (19 mg, 33%). ^1^H NMR (400 MHz, DMSO-*d*_6_) δ 7.39 (d, *J* = 2.0 Hz, 1H), 7.30–7.22 (m, 2H), 7.18 (d, *J* = 8.4 Hz, 1H), 7.01–6.91 (m, 3H), 6.81 (t, *J* = 5.6 Hz, 1H) 6.3 (m, 2H), 4.64 (hept, *J* = 6.8 Hz, 1H), 4.14 (h, *J* = 5.3 Hz, 1H), 4.06–3.90 (m, 4H), 3.55 (dt, *J* = 13.5, 5.6 Hz, 1H), 3.45 (ddd, *J* = 13.5, 6.7, 5.3 Hz, 1H), 1.48 (d, *J* = 6.8 Hz, 6H). LCMS *m*/*z* calcd for C_21_H_24_ClN_5_O [M + H]^+^: 397.2 found 398 with *t*_R_ 2.31 min.

## Supporting Information Available

Table of strains used in this study, figure of protein–ligand binding site, Figures of data related to final compounds HRMS, ^1^H NMR, ^13^C NMR, and LCMS (PDF)
Molecular formula strings (CSV)

## Abbreviations Used

AI: autoinducer
AQ: 2-alkyl-4(1*H*)-quinolone derived compound
Cip: ciprofloxacin
CLSM: confocal laser scanning microscope
CF: cystic fibrosis
DIAD: diisopropyl azodicarboxylate
DIC: *N*,*N*′-diisopropylcarbodiimide
HHQ: 2-heptyl-4-hydroxyquinoline
HQNO: 2-heptyl- 4-hydroxyquinoline *N*-oxide
LBD: ligand binding domain
PA: *Pseudomonas aeruginosa*
pqs: Pseudomonas quinolone signal
PQS: 2-heptyl-3-hydroxy-4(1*H*)-quinolone
TBDMS: *tert*-butyldimethylsilyl chloride
QS: quorum sensing
SD: standard deviation
WHO: World Health Organisation
