# Supplementary material for: Design, Synthesis, and Evaluation of New 1H-Benzo[d]imidazole Based PqsR Inhibitors as Adjuvant Therapy for Pseudomonas aeruginosa Infections
Source: J Med Chem. 2024 Jan 3;67(2):1008–23. doi: 10.1021/acs.jmedchem.3c00973 (PMC10823468; doi:10.1021/acs.jmedchem.3c00973)
Supplement: Supplementary file 2 — jm3c00973_si_002.pdf [file jm3c00973_si_002.pdf]

**Supporting Information**

# Design, Synthesis and Evaluation of New 1*H*-benzo[*d*]imidazole based PqsR Inhibitors as Adjuvant Therapy for *Pseudomonas aeruginosa* Infections

*Fadi Soukarieh[*a,1,3], Alaa Mashabi[a,2,ɼ], William Richardson[2], Eduard Vico Oton[1‡], Manuel Romero[1,3|], Jean-Frédéric Dubern[1], Shaun N. Robertson[1,3], Simone Lucanto[1,3], Zoe Markham-Lee[2], Tomás Sou[4‖], Irena Kukavica-Ibrulj[5], Roger C. Levesque[5], Christel A.S. Bergstrom[4], Nigel Halliday[1], Barrie Kellam[2], Jonas Emsley[2,3], Stephan Heeb[1], Paul Williams[1,3], Michael J. Stocks[*2,3], and Miguel Cámara[*1,3].*

[a] These authors contributed equally to this study.

[1] School of Life Sciences, University of Nottingham Biodiscovery Institute, University of Nottingham, Nottingham, NG7 2RD, UK.

[2] School of Pharmacy, University of Nottingham Biodiscovery Institute, University of Nottingham, Nottingham, NG7 2RD, UK.

[3] The National Biofilms Innovation Centre, University of Nottingham Biodiscovery Institute, University of Nottingham, Nottingham, NG7 2RD, UK.

[4] Department of Pharmacy, Uppsala University, Uppsala, SE-751 23, Sweden

[5] Institut de Biologie Intégrative et des Systèmes, Université Laval, Quebec G1V 0A6, Canada.

Corresponding Authors

Dr Fadi Soukarieh

fadi.soukarieh@nottingham.ac.uk

Professor Michael Stocks

michael.stocks@nottingham.ac.uk

Professor Miguel Cámara,

miguel.camara@nottingham.ac.uk

# Table of Contents

**Table S1** **S4**

**Figures S1-S41** **S5**

**References** **S37**

**Table S1.** Bacterial strains and plasmids used in this study

| **Strain or plasmid** | **Relevant characteristics** | **Reference or origin** |
|---|---|---|
| PAO1-L | Wound isolate, wild type PAO1, Lausanne subline. | B. Holloway, *via* D. Haas |
| PAO1-L mCTX::$P_{pqsA}$-*lux* | PAO1-L with chromosomal mini-CTX::$P_{pqsA}$-*lux* insertion; $Tc^R$ | [1] |
| PAO1-N | Wound isolate, wild type PAO1, Nottingham subline | [2] |
| PA14 | Burn isolate, wild type UCBPP-PA14 | [3, 4] |
| PALESB58 | CF isolate | [5] |
| IPCD1451 | CF isolate, >95% related to PAO1, IPCD ID 1451 | [6, 7] |
| IPCD1350 | CF isolate, >95% related to PA14, IPCD ID 1350 | [6, 7] |
| IPCD1331 | CF isolate, >95% related to PAK, IPCD ID 1331 | [6, 7] |
| IPCD48 | CF isolate, >95% related to PA7, IPCD ID 48 | [6-8] |
| PA14 mCTX::$P_{pqsA}$-*lux* | PA14 with chromosomal mini-CTX::$P_{pqsA}$-*lux* insertion; $Tc^R$ | [1] |
| <u>Plasmids</u> | | |
| mini-CTX::$P_{pqsA}$-*lux* | R6K-based mini-CTX suicide plasmid for the chromosomal insertion of a $P_{pqsA}$-*lux* transcriptional reporter fusion; $Tc^R$ | [9] |
| pMMG | pME6032Δ*lacI* constitutively expressing GFP from the $P_{tac}$ promoter | [10] |

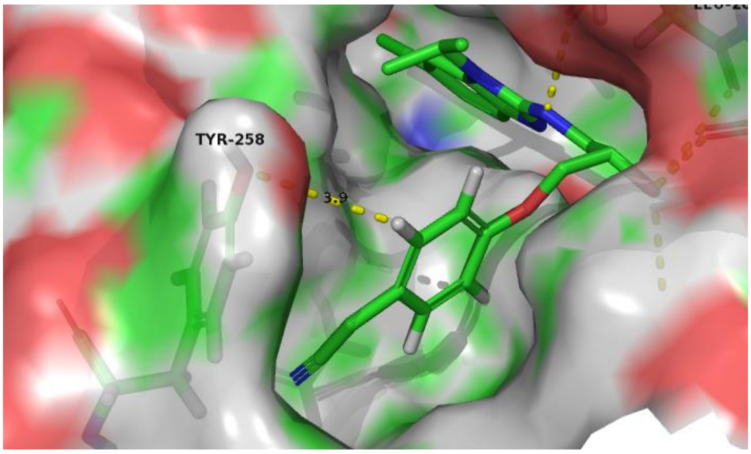


Figure S1: Schematic representation of the measurement between the *ortho*- position of the phenoxy ring of **6f** and the oxygen atom of the Tyrosine 258 (PqsR ligand binding domain).

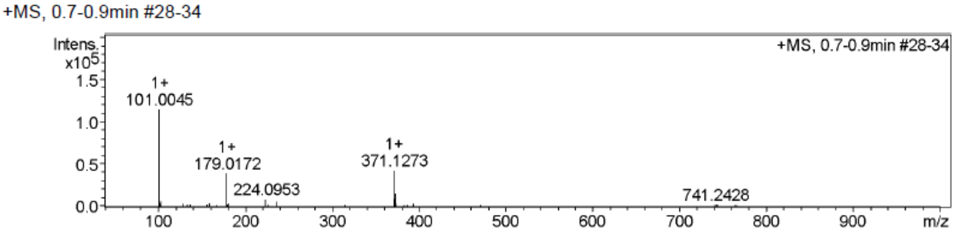


| # | m/z | I % |
|---|---|---|
| 1 | 101.0045 | 100.0 |
| 2 | 102.0068 | 3.4 |
| 3 | 103.0006 | 5.9 |
| 4 | 129.0145 | 2.9 |
| 5 | 137.0024 | 2.2 |
| 6 | 157.0359 | 2.1 |
| 7 | 158.9647 | 4.1 |
| 8 | 179.0172 | 34.0 |
| 9 | 180.0192 | 2.2 |
| 10 | 181.0141 | 3.5 |
| 11 | 224.0953 | 7.3 |
| 12 | 226.0920 | 2.6 |
| 13 | 236.9784 | 5.5 |
| 14 | 371.1273 | 37.0 |
| 15 | 372.1300 | 8.5 |
| 16 | 373.1250 | 13.4 |
| 17 | 374.1275 | 2.8 |
| 18 | 393.1090 | 2.8 |
| 19 | 741.2428 | 2.6 |
| 20 | 743.2422 | 2.0 |

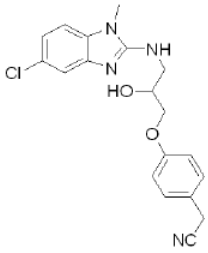


**Generate Molecular Formula Parameters**

| Charge | Tolerance | sigma limit | H/C Ratio | Electron Conf. | Nitrogen Rule | Chrom.BackGround | Calibration |
|---|---|---|---|---|---|---|---|
| +1 | 6 ppm | 0.08 | 3 - 0 | both | false | false | TRUE |

**Expected Formula** C19 H19 N4 O2 **Adduct(s):** H, Na, NH4, C3H5N2, radical

Note: Sigma fits < 0.05 indicates high probability of correct MF

Figure S2. HRMS spectrum of **6b**.

+MS, 0.7-0.9min #28-34

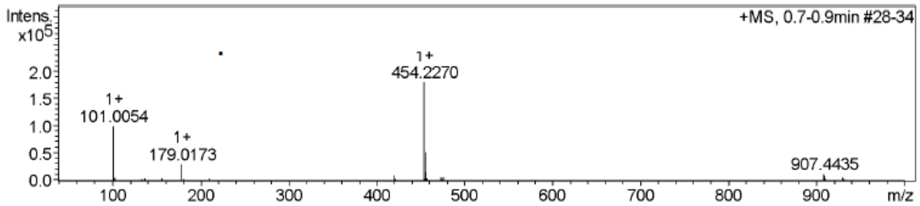


| # | m/z | I % |
|---|---|---|
| 1 | 101.0054 | 55.1 |
| 2 | 102.0076 | 1.8 |
| 3 | 103.0014 | 3.2 |
| 4 | 137.0025 | 2.0 |
| 5 | 157.0361 | 2.0 |
| 6 | 179.0173 | 16.6 |
| 7 | 181.0129 | 1.8 |
| 8 | 210.6240 | 2.1 |
| 9 | 420.2391 | 4.9 |
| 10 | 454.2270 | 100.0 |
| 11 | 455.2300 | 28.7 |
| 12 | 456.2274 | 9.2 |
| 13 | 457.2282 | 2.0 |
| 14 | 473.2203 | 3.9 |
| 15 | 476.2095 | 3.6 |
| 16 | 907.4435 | 7.1 |
| 17 | 908.4488 | 4.2 |
| 18 | 909.4483 | 2.0 |
| 19 | 929.4252 | 2.7 |
| 20 | 930.4261 | 1.6 |

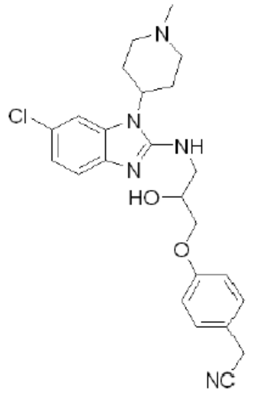


**Generate Molecular Formula Parameters**

| Charge | Tolerance | sigma limit | H/C Ratio | Electron Conf. | Nitrogen Rule | Chrom.BackGround | Calibration |
|---|---|---|---|---|---|---|---|
| +1 | 6 ppm | 0.08 | 3 - 0 | both | false | false | TRUE |

**Expected Formula** C24 H28 Cl1 N5 O2 **Adduct(s):** H, Na, NH4, C3H5N2, radical

Note: Sigma fits < 0.05 indicates high probability of correct MF

Figure S3. HRMS spectrum of **6r**.

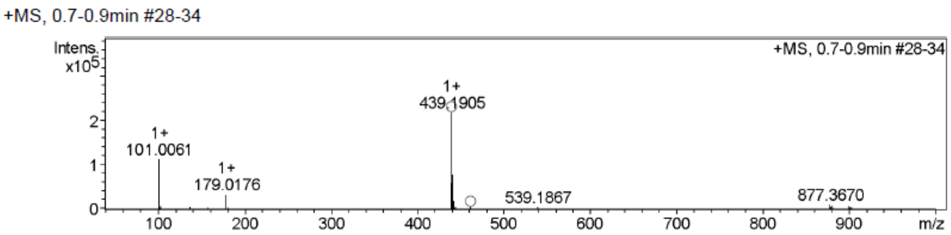


| # | m/z | I % |
|---|---|---|
| 1 | 101.0061 | 51.9 |
| 2 | 102.0088 | 1.8 |
| 3 | 103.0023 | 3.0 |
| 4 | 137.0034 | 2.4 |
| 5 | 179.0176 | 14.8 |
| 6 | 181.0136 | 1.5 |
| 7 | 439.1905 | 100.0 |
| 8 | 440.1926 | 27.4 |
| 9 | 441.1879 | 35.4 |
| 10 | 442.1904 | 9.0 |
| 11 | 444.1688 | 2.2 |
| 12 | 461.1712 | 2.5 |
| 13 | 539.1867 | 2.1 |
| 14 | 877.3670 | 4.3 |
| 15 | 878.3705 | 2.5 |
| 16 | 879.3682 | 3.4 |
| 17 | 880.3683 | 1.7 |
| 18 | 899.3485 | 3.1 |
| 19 | 900.3522 | 1.8 |
| 20 | 901.3516 | 2.4 |

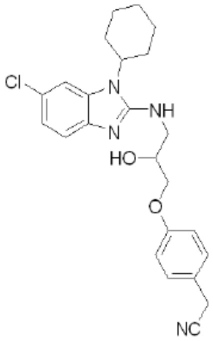


**Generate Molecular Formula Parameters**

| Charge | Tolerance | sigma limit | H/C Ratio | Electron Conf. | Nitrogen Rule | Chrom.BackGround | Calibration |
|---|---|---|---|---|---|---|---|
| +1 | 6 ppm | 0.08 | 3 - 0 | both | false | false | TRUE |

**Expected Formula** C24 H27 Cl1 N4 O2 **Adduct(s):** H, Na, NH4, C3H5N2, radical

| # | meas. m/z | theo. m/z | \|Err\|[ppm] | Sigma | Formula | Adduct | Adduct Mass |
|---|---|---|---|---|---|---|---|
| 1 | 439.1905 | 439.1895 | 2.10 | 0.0043 | C24H28ClN4O2 | M+H | 1.0078 |
| 1 | 461.1712 | 461.1715 | 0.60 | 0.0121 | C24H27ClN4NaO2 | M+Na | 22.9898 |

Note: Sigma fits < 0.05 indicates high probability of correct MF

Figure S4. HRMS spectrum of **6l**.

+MS, 0.7-0.9min #28-34

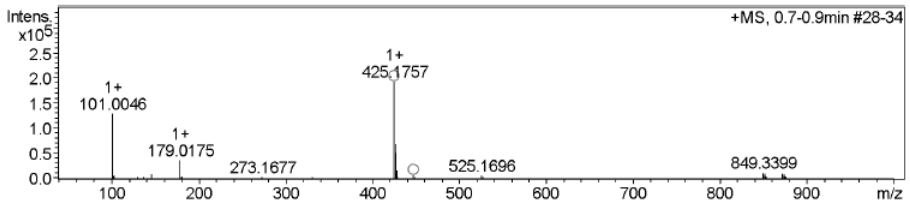


| # | m/z | I % |
|---|---|---|
| 1 | 101.0046 | 66.4 |
| 2 | 102.0065 | 2.3 |
| 3 | 103.0007 | 3.7 |
| 4 | 146.1549 | 4.4 |
| 5 | 179.0175 | 18.7 |
| 6 | 181.0139 | 2.0 |
| 7 | 425.1757 | 100.0 |
| 8 | 426.1781 | 26.6 |
| 9 | 427.1732 | 35.9 |
| 10 | 428.1752 | 9.1 |
| 11 | 447.1562 | 3.5 |
| 12 | 525.1696 | 2.7 |
| 13 | 849.3399 | 6.3 |
| 14 | 850.3423 | 3.6 |
| 15 | 851.3395 | 5.1 |
| 16 | 852.3412 | 2.4 |
| 17 | 871.3205 | 5.3 |
| 18 | 872.3266 | 2.7 |
| 19 | 873.3179 | 4.2 |
| 20 | 874.3188 | 2.0 |

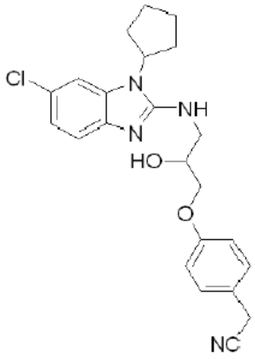


**Generate Molecular Formula Parameters**

| Charge | Tolerance | sigma limit | H/C Ratio | Electron Conf. | Nitrogen Rule | Chrom.BackGround | Calibration |
|---|---|---|---|---|---|---|---|
| +1 | 6 ppm | 0.08 | 3 - 0 | both | false | false | TRUE |

**Expected Formula** C23 H25 Cl1 N4 O2 **Adduct(s):** H, Na, NH4, C3H5N2, radical

| # | meas. m/z | theo. m/z | \|Err\|[ppm] | Sigma | Formula | Adduct | Adduct Mass |
|---|---|---|---|---|---|---|---|
| 1 | 425.1757 | 425.1739 | 4.40 | 0.0011 | C23H26ClN4O2 | M+H | 1.0078 |
| 1 | 447.1562 | 447.1558 | 0.90 | 0.0200 | C23H25ClN4NaO2 | M+Na | 22.9898 |

Note: Sigma fits < 0.05 indicates high probability of correct MF

Figure S5. HRMS spectrum of **6k**.

+MS, 0.7-0.9min #28-34

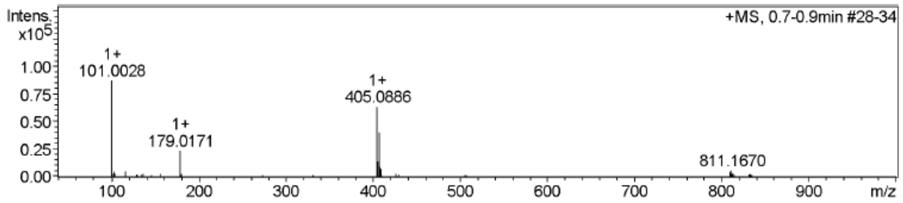


| # | m/z | I % |
|---|---|---|
| 1 | 101.0028 | 100.0 |
| 2 | 102.0054 | 3.3 |
| 3 | 102.9988 | 5.9 |
| 4 | 104.1069 | 4.0 |
| 5 | 116.9773 | 6.4 |
| 6 | 137.0016 | 2.9 |
| 7 | 157.0359 | 2.9 |
| 8 | 179.0171 | 27.3 |
| 9 | 181.0142 | 2.9 |
| 10 | 405.0886 | 72.6 |
| 11 | 406.0916 | 16.0 |
| 12 | 407.0859 | 46.7 |
| 13 | 408.0869 | 10.8 |
| 14 | 409.0846 | 8.4 |
| 15 | 427.0733 | 2.8 |
| 16 | 809.1675 | 5.1 |
| 17 | 811.1670 | 6.8 |
| 18 | 812.1694 | 2.9 |
| 19 | 813.1632 | 3.5 |
| 20 | 833.1458 | 3.2 |

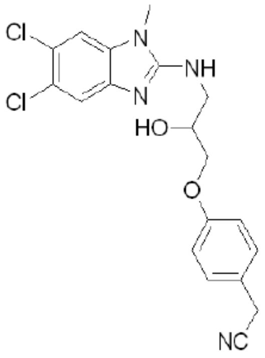


**Generate Molecular Formula Paramete**

| Charge | Tolerance | sigma limit | H/C Ratio | Electron Conf. | Nitrogen Rule | Chrom.BackGround | Calibration |
|---|---|---|---|---|---|---|---|
| +1 | 6 ppm | 0.08 | 3 - 0 | both | false | false | TRUE |

**Expected Formula** C21 H22 Cl2 N4 O2 **Adduct(s):** H, Na, NH4, C3H5N2, radical

Note: Sigma fits < 0.05 indicates high probability of correct MF

Figure S6. HRMS spectrum of **6c**.

+MS, 0.7-0.9min #28-34

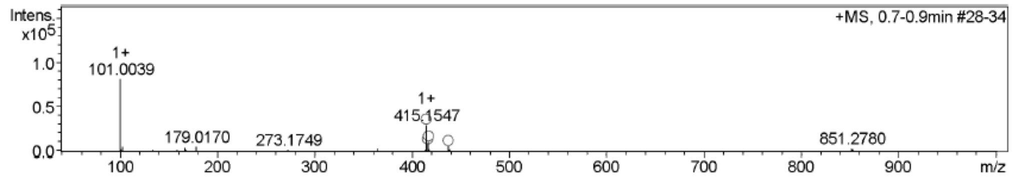


| # | m/z | I % |
|---|---|---|
| 1 | 101.0039 | 100.0 |
| 2 | 102.0065 | 3.3 |
| 3 | 102.9999 | 6.0 |
| 4 | 134.0813 | 2.1 |
| 5 | 167.1158 | 5.5 |
| 6 | 168.9902 | 1.5 |
| 7 | 179.0170 | 6.5 |
| 8 | 273.1749 | 2.1 |
| 9 | 365.1078 | 3.9 |
| 10 | 413.1776 | 1.9 |
| 11 | 415.1547 | 36.4 |
| 12 | 416.1563 | 9.1 |
| 13 | 417.1518 | 12.9 |
| 14 | 418.1531 | 3.1 |
| 15 | 437.1360 | 7.3 |
| 16 | 438.1403 | 1.7 |
| 17 | 439.1336 | 2.7 |
| 18 | 851.2780 | 4.2 |
| 19 | 852.2757 | 2.2 |
| 20 | 853.2769 | 3.2 |

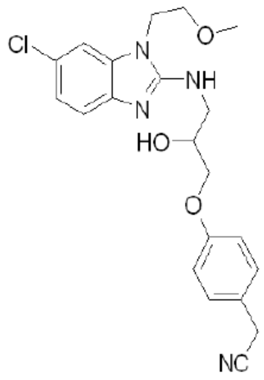


**Generate Molecular Formula Parameters**

No of Most Intense MS Peaks Analysed 5

| Charge | Tolerance | sigma_limit | Calibration |
|---|---|---|---|
| +1 | 6 ppm | 0.08 | TRUE |

**Formula Min** C 1 H 0 N 0 O 0 **Formula Max** C50 H100 N10 O10 H Na NH4 C3H5N2

| # | meas. m/z | theo. m/z | \|Err\|[ppm] | Sigma | Formula |
|---|---|---|---|---|---|
| 2 | 417.1518 | 417.1520 | 0.50 | 0.0038 | C 19 H 20 N 7 Na 1 O 3 |
| | | 417.1533 | 3.70 | 0.0040 | C 21 H 22 N 4 Na 1 O 4 |
| | | 417.1531 | 3.10 | 0.0040 | C 20 H 23 N 3 O 7 |
| | | 417.1530 | 3.10 | 0.0042 | C 19 H 17 N 10 O 2 |
| | | 417.1517 | 0.20 | 0.0061 | C 17 H 15 N 13 O 1 |

Note: Sigma fits < 0.05 indicates high probability of correct MF

Figure S7. HRMS spectrum of **6t**.

| | | | |
|---|---|---|---|
| Sample-ID | pazfs2_005 | Lab | C13 |
| Submitter | Fadi Soukarieh (pazfs2) | Supervisor | Mike Stocks (pazmjs) |
| Analysis Name | pazfs2_005_653078_7_01_133763.d | Acquisition Date | 8/9/2023 10:43:23 AM |
| Ionisation Mode | ESI Positive | Instrument | Bruker MicroTOF |

+MS, 0.7-0.9min #27-33

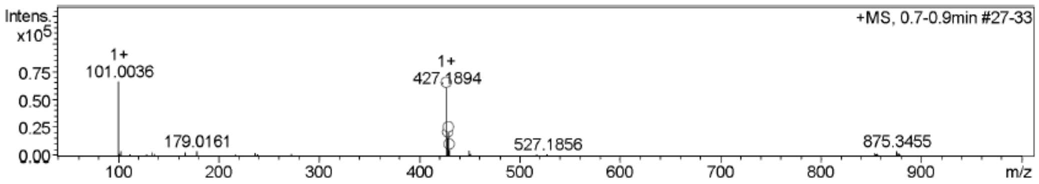


| # | m/z | I % |
|---|---|---|
| 1 | 101.0036 | 100.0 |
| 2 | 102.0060 | 3.4 |
| 3 | 102.9999 | 5.8 |
| 4 | 134.0808 | 5.1 |
| 5 | 137.0016 | 2.8 |
| 6 | 167.1152 | 5.1 |
| 7 | 179.0161 | 5.9 |
| 8 | 237.1141 | 4.2 |
| 9 | 273.1758 | 2.8 |
| 10 | 427.1894 | 91.0 |
| 11 | 428.1919 | 23.9 |
| 12 | 429.1874 | 32.0 |
| 13 | 430.1906 | 8.0 |
| 14 | 449.1710 | 7.1 |
| 15 | 451.1675 | 2.7 |
| 16 | 853.3696 | 4.3 |
| 17 | 855.3724 | 3.1 |
| 18 | 875.3455 | 5.6 |
| 19 | 876.3515 | 3.0 |
| 20 | 877.3517 | 4.4 |

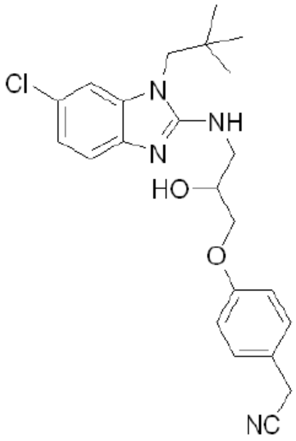


**Generate Molecular Formula**

tense MS Peaks Analysed 5

| Charge | Tolerance | sigma_lim | Calibration |
|---|---|---|---|
| +1 | 6 ppm | 0.08 | TRUE |

**Formula Min** C 1 H 0 N 0 O 0 **Formula Max** C50 H100 N10 O10 H Na NH4 C3H5N2

| # | meas. m/z | theo. m/z | \|Err\|[ppm] | Sigma | Formula |
|---|---|---|---|---|---|
| 2 | 429.1874 | 429.1894 | 4.80 | 0.0029 | C 22 H 27 N 3 O 6 |
| | | 429.1884 | 2.30 | 0.0032 | C 22 H 30 Na 1 O 7 |
| | | 429.1881 | 1.70 | 0.0050 | C 20 H 25 N 6 O 5 |
| | | 429.1884 | 2.30 | 0.0065 | C 21 H 24 N 7 Na 1 O 2 |
| | | 429.1870 | 0.80 | 0.0083 | C 19 H 22 N 10 Na 1 O 1 |
| 4 | 430.1906 | 430.1881 | 5.70 | 0.0167 | C 11 H 25 N 11 Na 1 O 6 |
| | | 430.1895 | 2.60 | 0.0168 | C 13 H 27 N 8 Na 1 O 7 |

Figure S8. HRMS spectrum of **6h**.

| | | | |
|---|---|---|---|
| Sample-ID | pazfs2_006 | Lab | C13 |
| Submitter | Fadi Soukarieh (pazfs2) | Supervisor | Mike Stocks (pazmjs) |
| Analysis Name | pazfs2_006_653079_8_01_133764.d | Acquisition Date | 8/9/2023 10:45:53 AM |
| Ionisation Mode | ESI Positive | Instrument | Bruker MicroTOF |

+MS, 0.7-0.9min #28-34

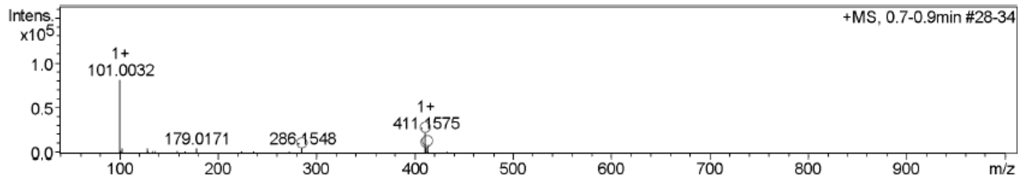


| # | m/z | I % |
|---|---|---|
| 1 | 101.0032 | 100.0 |
| 2 | 102.0056 | 3.5 |
| 3 | 102.9990 | 6.3 |
| 4 | 129.0125 | 5.8 |
| 5 | 130.0110 | 1.8 |
| 6 | 134.0808 | 3.3 |
| 7 | 137.0010 | 2.9 |
| 8 | 158.9633 | 2.9 |
| 9 | 167.1170 | 1.8 |
| 10 | 179.0171 | 6.1 |
| 11 | 225.0114 | 1.8 |
| 12 | 236.9792 | 1.8 |
| 13 | 273.1758 | 2.1 |
| 14 | 286.1548 | 6.4 |
| 15 | 292.9943 | 1.5 |
| 16 | 411.1575 | 26.9 |
| 17 | 412.1616 | 7.1 |
| 18 | 413.1560 | 9.4 |
| 19 | 414.1579 | 2.5 |
| 20 | 433.1407 | 2.3 |

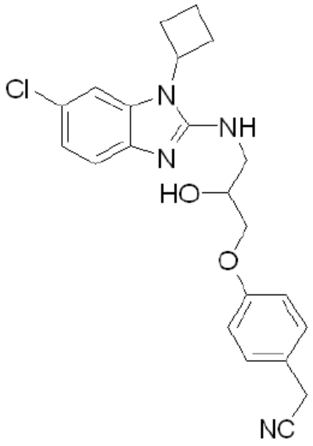


**Generate Molecular Formula P**

[illegible]MS Peaks Analysed 5

| Charge | Tolerance | sigma_limit | Calibration |
|---|---|---|---|
| +1 | 6 ppm | 0.08 | TRUE |

**Formula Min** C 1 H 0 N 0 O 0 **Formula Max** C50 H100 N10 O10 H Na NH4 C3H5N2

| # | meas. m/z | theo. m/z | \|Err\|[ppm] | Sigma | Formula |
|---|---|---|---|---|---|
| 2 | 413.1560 | 413.1584 | 5.90 | 0.0088 | C 22 H 22 N 4 Na 1 O 3 |
| | | 413.1581 | 5.30 | 0.0114 | C 20 H 17 N 10 O 1 |
| | | 413.1581 | 5.30 | 0.0122 | C 21 H 23 N 3 O 6 |
| | | 413.1571 | 2.70 | 0.0144 | C 20 H 20 N 7 Na 1 O 2 |
| | | 413.1568 | 2.00 | 0.0167 | C 18 H 15 N 13 |
| 4 | 286.1548 | 286.1550 | 0.80 | 0.0061 | C 16 H 20 N 3 O 2 |
| | | 286.1563 | 5.50 | 0.0079 | C 18 H 22 O 3 |

Figure S9. HRMS spectrum of **6j**.

| Sample-ID | pazfs2_007 | Lab | C13 |
|---|---|---|---|
| Submitter | Fadi Soukarieh (pazfs2) | Supervisor | Mike Stocks (pazmjs) |
| Analysis Name | pazfs2_007_653080_9_01_133765.d | Acquisition Date | 8/9/2023 10:48:23 AM |
| Ionisation Mode | ESI Positive | Instrument | Bruker MicroTOF |

+MS, 0.7-0.9min #28-34

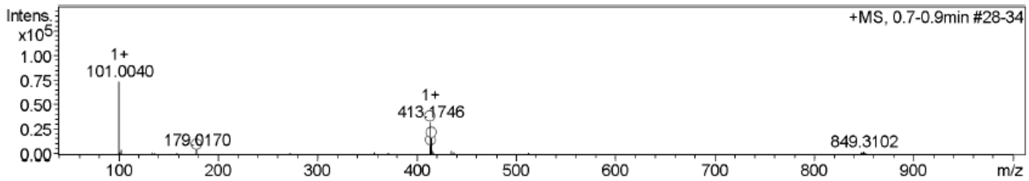


| # | m/z | I % |
|---|---|---|
| 1 | 101.0040 | 100.0 |
| 2 | 102.0061 | 3.5 |
| 3 | 103.0002 | 5.9 |
| 4 | 134.0816 | 3.3 |
| 5 | 137.0021 | 1.8 |
| 6 | 179.0170 | 6.0 |
| 7 | 273.1757 | 1.8 |
| 8 | 357.1091 | 3.3 |
| 9 | 413.1746 | 44.6 |
| 10 | 414.1762 | 11.7 |
| 11 | 415.1666 | 22.4 |
| 12 | 416.1689 | 5.5 |
| 13 | 417.1542 | 3.4 |
| 14 | 435.1555 | 5.6 |
| 15 | 437.1462 | 3.1 |
| 16 | 513.1700 | 1.8 |
| 17 | 847.3205 | 3.0 |
| 18 | 849.3102 | 3.7 |
| 19 | 850.3139 | 1.8 |
| 20 | 851.3059 | 1.7 |

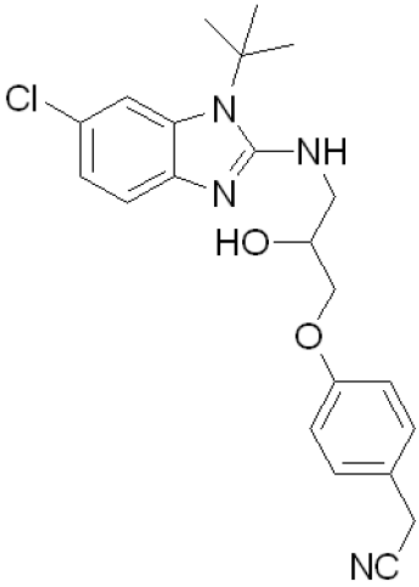


**Generate Molecular Formula Parameter:**

| Charge | Tolerance | sigma_limit | C |
|---|---|---|---|
| +1 | 6 ppm | 0.08 | |

**Formula Min** C 1 H 0 N 0 O 0

| # | meas. m/z | theo. m/z | \|Err\|[ppm] | | |
|---|---|---|---|---|---|
| 1 | 413.1746 | 413.1721 | 6.10 | | |
| | | 413.1723 | 5.40 | 0.0267 | C 25 H 26 Na 1 O 4 |
| | | 413.1734 | 2.80 | 0.0293 | C 25 H 23 N 3 O 3 |
| | | 413.1737 | 2.20 | 0.0323 | C 26 H 22 N 4 Na 1 |
| | | 413.1747 | 0.40 | 0.0352 | C 27 H 25 O 4 |
| 2 | 415.1666 | 415.1642 | 5.90 | 0.0305 | C 24 H 20 N 6 Na 1 |
| | | 415.1652 | 3.30 | 0.0332 | C 25 H 23 N 2 O 4 |

Figure S10. HRMS spectrum of **6g**.

| | | | |
|---|---|---|---|
| Sample-ID | pazfs2_008 | Lab | C13 |
| Submitter | Fadi Soukarieh (pazfs2) | Supervisor | Mike Stocks (pazmjs) |
| Analysis Name | pazfs2_008_653081_10_01_133766.d | Acquisition Date | 8/9/2023 10:50:52 AM |
| Ionisation Mode | ESI Positive | Instrument | Bruker MicroTOF |

+MS, 0.7-0.9min #28-34

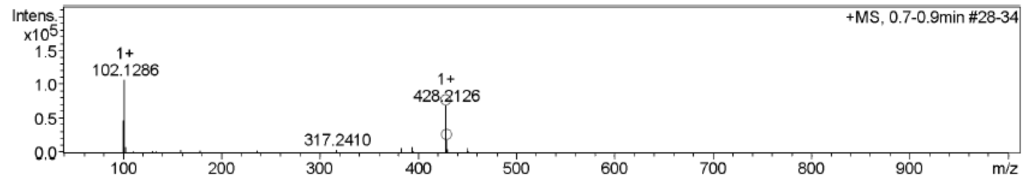


| # | m/z | I % |
|---|---|---|
| 1 | 101.0041 | 44.2 |
| 2 | 102.1286 | 100.0 |
| 3 | 103.0004 | 2.9 |
| 4 | 103.1316 | 7.9 |
| 5 | 111.0932 | 1.7 |
| 6 | 130.1593 | 2.2 |
| 7 | 134.0810 | 1.8 |
| 8 | 158.9636 | 3.7 |
| 9 | 179.0170 | 3.0 |
| 10 | 236.9775 | 2.5 |
| 11 | 317.2410 | 4.0 |
| 12 | 383.1532 | 6.6 |
| 13 | 384.1596 | 1.5 |
| 14 | 394.2239 | 7.7 |
| 15 | 395.2284 | 1.9 |
| 16 | 428.2126 | 64.7 |
| 17 | 429.2150 | 17.0 |
| 18 | 430.2134 | 5.5 |
| 19 | 450.1931 | 5.9 |
| 20 | 451.1972 | 1.6 |

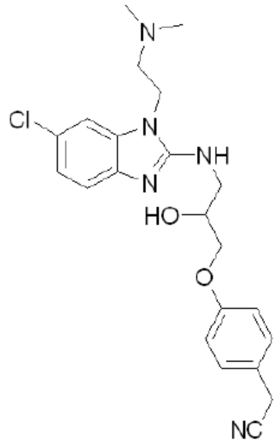


**Generate Molecular Formula Parameters**

No of Most Intense MS Peaks Analysed 5

| Charge | Tolerance | sigma_limit | Calibration |
|---|---|---|---|
| +1 | 6 ppm | 0.08 | TRUE |

**Formula Min** C 1 H 0 N 0 O 0 **Formula Max** C50 H100 N10 O10 H Na NH4 C3H5N2

| # | meas. m/z | theo. m/z | \|Err\|[ppm] | Sigma | Formula |
|---|---|---|---|---|---|
| 1 | 428.2126 | 428.2142 | 3.80 | 0.0348 | C 19 H 29 N 6 Na 1 O 4 |
| | | 428.2140 | 3.10 | 0.0372 | C 17 H 24 N 12 O 2 |
| | | 428.2140 | 3.10 | 0.0379 | C 18 H 30 N 5 O 7 |
| | | 428.2129 | 0.70 | 0.0406 | C 17 H 27 N 9 Na 1 O 3 |
| | | 428.2111 | 3.70 | 0.0415 | C 30 H 29 Na 1 O 1 |
| 2 | 429.2150 | 429.2175 | 6.00 | 0.0110 | C 28 H 28 N 3 Na 1 |
| | | 429.2173 | 5.30 | 0.0150 | C 27 H 29 N 2 O 3 |

Figure S11. HRMS spectrum of **6q**.

| | | | |
|---|---|---|---|
| Sample-ID | pazfs2_021 | Lab | C13 |
| Submitter | Fadi Soukarieh (pazfs2) | Supervisor | Mike Stocks (pazmjs) |
| Analysis Name | pazfs2_021_653086_12_01_133769.d | Acquisition Date | 8/9/2023 12:21:18 PM |
| Ionisation Mode | ESI Positive | Instrument | Bruker MicroTOF |

+MS, 0.7-0.9min #27-33

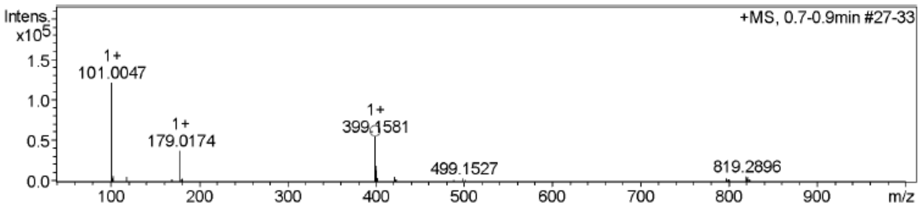


| # | m/z | I % |
|---|---|---|
| 1 | 101.0047 | 100.0 |
| 2 | 102.0070 | 3.4 |
| 3 | 103.0011 | 5.8 |
| 4 | 118.1239 | 4.7 |
| 5 | 168.9908 | 2.1 |
| 6 | 179.0174 | 31.2 |
| 7 | 180.0189 | 2.0 |
| 8 | 181.0132 | 3.1 |
| 9 | 399.1581 | 45.2 |
| 10 | 400.1610 | 11.2 |
| 11 | 401.1542 | 15.9 |
| 12 | 402.1572 | 3.7 |
| 13 | 421.1396 | 4.8 |
| 14 | 499.1527 | 3.2 |
| 15 | 797.3108 | 3.3 |
| 16 | 799.3009 | 2.5 |
| 17 | 819.2896 | 5.6 |
| 18 | 820.2915 | 2.8 |
| 19 | 821.2826 | 4.4 |
| 20 | 822.2857 | 2.0 |

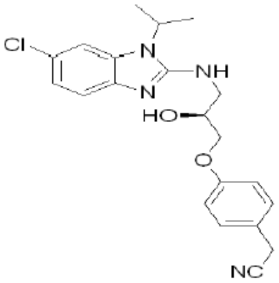


**Generate Molecular Formula Parameters**

| Charge | Tolerance | sigma limit | H/C Ratio | Electron Conf. | Nitrogen Rule | Chrom.BackGround | Calibration |
|---|---|---|---|---|---|---|---|
| +1 | 6 ppm | 0.08 | 3 - 0 | both | false | false | TRUE |

**Expected Formula** C21 H24 Cl1 N4 O2 **Adduct(s):** H, Na, NH4, C3H5N2, radical

| # | meas. m/z | theo. m/z | \|Err\|[ppm] | Sigma | Formula | Adduct | Adduct Mass |
|---|---|---|---|---|---|---|---|
| 1 | 399.1581 | 399.1582 | 0.20 | 0.0016 | C21H24ClN4O2 | M+ | -0.0005 |

Note: Sigma fits < 0.05 indicates high probability of correct MF

Figure S12. HRMS spectrum of **6v**.

| | | | |
|---|---|---|---|
| Sample-ID | pazfs2_025 | Lab | C13 |
| Submitter | Fadi Soukarieh (pazfs2) | Supervisor | Mike Stocks (pazmjs) |
| Analysis Name | pazfs2_025_653087_13_01_133770.d | Acquisition Date | 8/9/2023 12:23:48 PM |
| Ionisation Mode | ESI Positive | Instrument | Bruker MicroTOF |

+MS, 0.7-0.9min #28-34

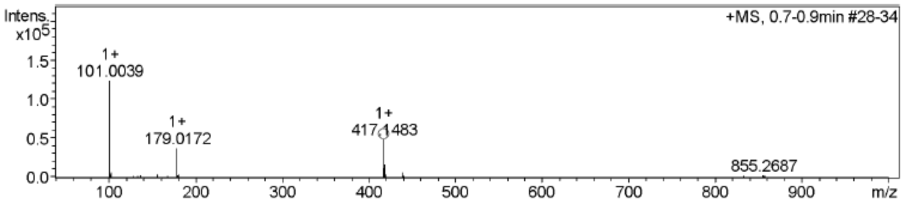


| # | m/z | I % |
|---|---|---|
| 1 | 101.0039 | 100.0 |
| 2 | 102.0063 | 3.4 |
| 3 | 103.0001 | 5.7 |
| 4 | 134.0823 | 1.7 |
| 5 | 137.0021 | 1.9 |
| 6 | 157.0356 | 3.2 |
| 7 | 168.9924 | 1.5 |
| 8 | 179.0172 | 30.0 |
| 9 | 180.0192 | 1.9 |
| 10 | 181.0133 | 3.2 |
| 11 | 417.1483 | 39.7 |
| 12 | 418.1514 | 10.0 |
| 13 | 419.1459 | 13.9 |
| 14 | 420.1485 | 3.3 |
| 15 | 439.1315 | 5.2 |
| 16 | 440.1303 | 1.3 |
| 17 | 441.1285 | 1.8 |
| 18 | 833.2846 | 1.3 |
| 19 | 855.2687 | 2.4 |
| 20 | 857.2652 | 1.9 |

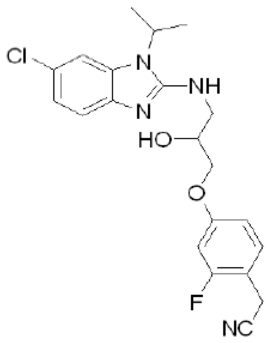


**Generate Molecular Formula Parameters**

| Charge | Tolerance | sigma limit | H/C Ratio | Electron Conf. | Nitrogen Rule | Chrom.BackGround | Calibration |
|---|---|---|---|---|---|---|---|
| +1 | 6 ppm | 0.08 | 3 - 0 | both | false | false | TRUE |

**Expected Formula** C21 H23 Cl1 F1 N4 O2 **Adduct(s):** H, Na, NH4, C3H5N2, radical

| # | meas. m/z | theo. m/z | \|Err\|[ppm] | Sigma | Formula | Adduct | Adduct Mass |
|---|---|---|---|---|---|---|---|
| 1 | 417.1483 | 417.1488 | 1.10 | 0.0039 | C21H23ClFN4O2 | M+ | -0.0005 |

Note: Sigma fits < 0.05 indicates high probability of correct MF

Figure S13. HRMS spectrum of **6o**.

Ionisation Mode ESI Positive Instrument Bruker MicroTOF

+MS, 0.7-0.9min #28-34

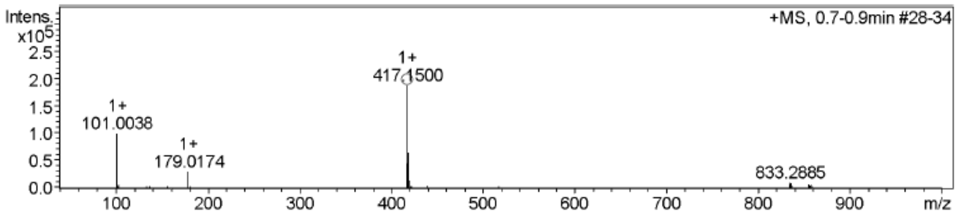


| # | m/z | I % |
|---|---|---|
| 1 | 101.0038 | 53.0 |
| 2 | 102.0062 | 1.8 |
| 3 | 102.9997 | 3.1 |
| 4 | 134.0815 | 1.6 |
| 5 | 157.0362 | 1.8 |
| 6 | 179.0174 | 15.8 |
| 7 | 181.0126 | 1.6 |
| 8 | 417.1500 | 100.0 |
| 9 | 418.1524 | 24.4 |
| 10 | 419.1474 | 34.4 |
| 11 | 420.1503 | 7.8 |
| 12 | 439.1327 | 2.2 |
| 13 | 517.1454 | 1.8 |
| 14 | 833.2885 | 5.6 |
| 15 | 834.2925 | 2.7 |
| 16 | 835.2883 | 4.3 |
| 17 | 836.2868 | 1.9 |
| 18 | 855.2703 | 3.9 |
| 19 | 856.2732 | 2.0 |
| 20 | 857.2697 | 2.9 |

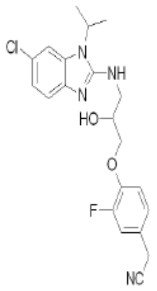


**Generate Molecular Formula Parameters**

| Charge | Tolerance | sigma limit | H/C Ratio | Electron Conf. | Nitrogen Rule | Chrom.BackGround | Calibration |
|---|---|---|---|---|---|---|---|
| +1 | 6 ppm | 0.08 | 3 - 0 | both | false | false | TRUE |

**Expected Formula** C21 H23 Cl1 F1 N4 O2 **Adduct(s):** H, Na, NH4, C3H5N2, radical

| # | meas. m/z | theo. m/z | \|Err\|[ppm] | Sigma | Formula | Adduct | Adduct Mass |
|---|---|---|---|---|---|---|---|
| 1 | 417.1500 | 417.1488 | 2.90 | 0.0042 | C21H23ClFN4O2 | M+ | -0.0005 |

Note: Sigma fits < 0.05 indicates high probability of correct MF

Figure S14. HRMS spectrum of **6n**.

| | | | |
|---|---|---|---|
| Sample-ID | pazfs2_sen086 | Lab | C13 |
| Submitter | Fadi Soukarieh (pazfs2) | Supervisor | Mike Stocks (pazmjs) |
| Analysis Name | pazfs2_sen086_653089_15_01_133772.d | Acquisition Date | 8/9/2023 12:28:48 PM |
| Ionisation Mode | ESI Positive | Instrument | Bruker MicroTOF |

+MS, 0.7-0.9min #28-34

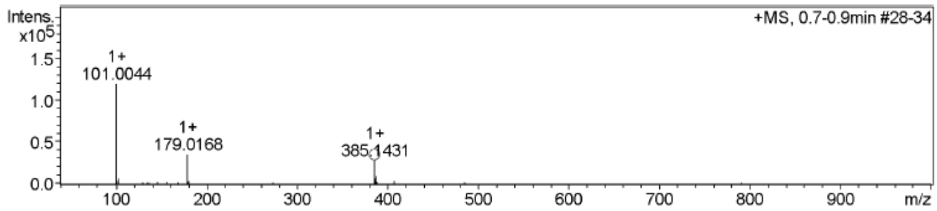


| # | m/z | I % |
|---|---|---|
| 1 | 101.0044 | 100.0 |
| 2 | 102.0071 | 3.3 |
| 3 | 103.0004 | 5.9 |
| 4 | 130.1595 | 1.4 |
| 5 | 134.0815 | 1.5 |
| 6 | 137.0031 | 1.7 |
| 7 | 146.1539 | 2.0 |
| 8 | 157.0351 | 2.6 |
| 9 | 168.9927 | 1.7 |
| 10 | 179.0168 | 29.6 |
| 11 | 180.0202 | 1.9 |
| 12 | 181.0132 | 2.9 |
| 13 | 273.1687 | 1.2 |
| 14 | 385.1431 | 23.8 |
| 15 | 386.1458 | 5.7 |
| 16 | 387.1402 | 8.7 |
| 17 | 388.1407 | 2.0 |
| 18 | 407.1266 | 2.7 |
| 19 | 485.1384 | 1.2 |
| 20 | 791.2580 | 1.3 |

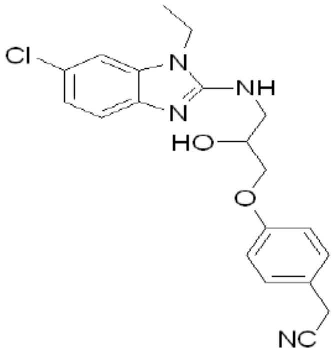


**Generate Molecular Formula Parameters**

| Charge | Tolerance | sigma limit | H/C Ratio | Electron Conf. | Nitrogen Rule | Chrom.BackGround | Calibration |
|---|---|---|---|---|---|---|---|
| +1 | 6 ppm | 0.08 | 3 - 0 | both | false | false | TRUE |

**Expected Formula** C20 H22 Cl1 N4 O2 **Adduct(s):** H, Na, NH4, C3H5N2, radical

| # | meas. m/z | theo. m/z | \|Err\|[ppm] | Sigma | Formula | Adduct | Adduct Mass |
|---|---|---|---|---|---|---|---|
| 1 | 385.1431 | 385.1426 | 1.40 | 0.0073 | C20H22ClN4O2 | M+ | -0.0005 |

Note: Sigma fits < 0.05 indicates high probability of correct MF

Figure S15. HRMS spectrum of **6e**.

| | | | |
|---|---|---|---|
| Sample-ID | pazfs2_SEN086_Me | Lab | C13 |
| Submitter | Fadi Soukarieh (pazfs2) | Supervisor | Mike Stocks (pazmjs) |
| Analysis Name | pazfs2_SEN086_Me_653090_16_01_1337 73.d | Acquisition Date | 8/9/2023 12:31:19 PM |
| Ionisation Mode | ESI Positive | Instrument | Bruker MicroTOF |

+MS, 0.7-0.9min #28-34

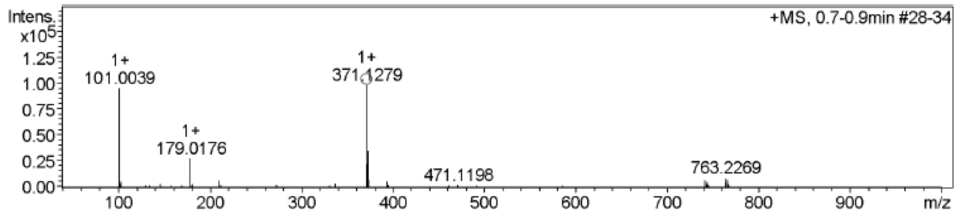


| # | m/z | I % |
|---|---|---|
| 1 | 101.0039 | 96.6 |
| 2 | 102.0062 | 3.3 |
| 3 | 103.0003 | 5.9 |
| 4 | 146.1540 | 2.8 |
| 5 | 179.0176 | 28.0 |
| 6 | 181.0145 | 2.7 |
| 7 | 210.0794 | 6.4 |
| 8 | 337.1651 | 3.7 |
| 9 | 371.1279 | 100.0 |
| 10 | 372.1300 | 23.0 |
| 11 | 373.1256 | 35.3 |
| 12 | 374.1277 | 7.7 |
| 13 | 393.1097 | 6.1 |
| 14 | 741.2448 | 6.7 |
| 15 | 742.2481 | 3.1 |
| 16 | 743.2423 | 5.3 |
| 17 | 763.2269 | 9.4 |
| 18 | 764.2334 | 4.7 |
| 19 | 765.2245 | 7.2 |
| 20 | 766.2307 | 3.0 |

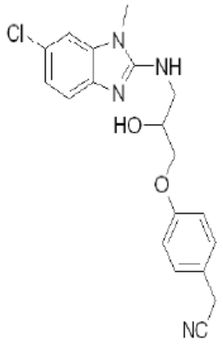


**Generate Molecular Formula Parameters**

| Charge | Tolerance | sigma limit | H/C Ratio | Electron Conf. | Nitrogen Rule | Chrom.BackGround | Calibration |
|---|---|---|---|---|---|---|---|
| +1 | 6 ppm | 0.08 | 3 - 0 | both | false | false | TRUE |

**Expected Formula** C19 H20 Cl1 N4 O2 **Adduct(s):** H, Na, NH4, C3H5N2, radical

| # | meas. m/z | theo. m/z | \|Err\|[ppm] | Sigma | Formula | Adduct | Adduct Mass |
|---|---|---|---|---|---|---|---|
| 1 | 371.1279 | 371.1269 | 2.60 | 0.0041 | C19H20ClN4O2 | M+ | -0.0005 |

Note: Sigma fits < 0.05 indicates high probability of correct MF

Figure S16. HRMS spectrum of **6a**.

+MS, 0.7-0.9min #28-34

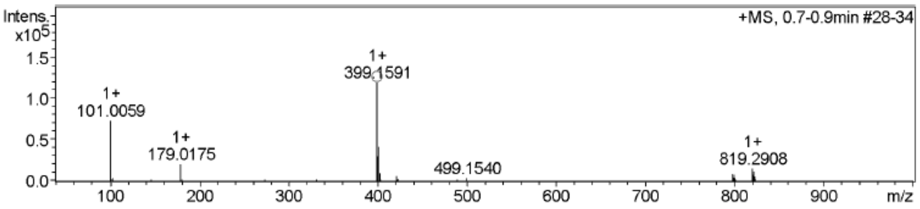


| # | m/z | I % |
|---|---|---|
| 1 | 101.0059 | 61.2 |
| 2 | 102.0085 | 2.2 |
| 3 | 103.0020 | 3.4 |
| 4 | 179.0175 | 17.3 |
| 5 | 399.1591 | 100.0 |
| 6 | 400.1623 | 25.1 |
| 7 | 401.1557 | 34.8 |
| 8 | 402.1583 | 8.6 |
| 9 | 421.1404 | 5.7 |
| 10 | 423.1391 | 2.0 |
| 11 | 499.1540 | 2.8 |
| 12 | 797.3091 | 7.8 |
| 13 | 798.3095 | 4.1 |
| 14 | 799.3053 | 6.5 |
| 15 | 800.3066 | 2.8 |
| 16 | 819.2908 | 12.9 |
| 17 | 820.2933 | 6.5 |
| 18 | 821.2869 | 10.2 |
| 19 | 822.2887 | 4.3 |
| 20 | 823.2854 | 2.6 |

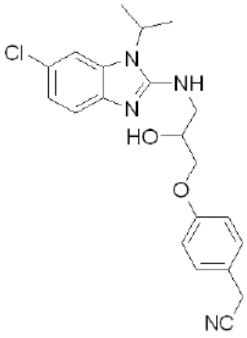


**Generate Molecular Formula Parameters**

| Charge | Tolerance | sigma limit | H/C Ratio | Electron Conf. | Nitrogen Rule | Chrom.BackGround | Calibration |
|---|---|---|---|---|---|---|---|
| +1 | 6 ppm | 0.08 | 3 - 0 | both | false | false | TRUE |

**Expected Formula** C21 H24 Cl1 N4 O2 **Adduct(s):** H, Na, NH4, C3H5N2, radical

| # | meas. m/z | theo. m/z | \|Err\|[ppm] | Sigma | Formula | Adduct | Adduct Mass |
|---|---|---|---|---|---|---|---|
| 1 | 399.1591 | 399.1582 | 2.20 | 0.0037 | C21H24ClN4O2 | M+ | -0.0005 |

Note: Sigma fits < 0.05 indicates high probability of correct MF

Figure S17. HRMS spectrum of **6f**.

| | | | |
|---|---|---|---|
| Sample-ID | pazfs2_SEN089 | Lab | C13 |
| Submitter | Fadi Soukarieh (pazfs2) | Supervisor | Mike Stocks (pazmjs) |
| Analysis Name | pazfs2_SEN089_653091_17_01_133774.d | Acquisition Date | 8/9/2023 12:33:49 PM |
| Ionisation Mode | ESI Positive | Instrument | Bruker MicroTOF |

+MS, 0.7-0.9min #28-34

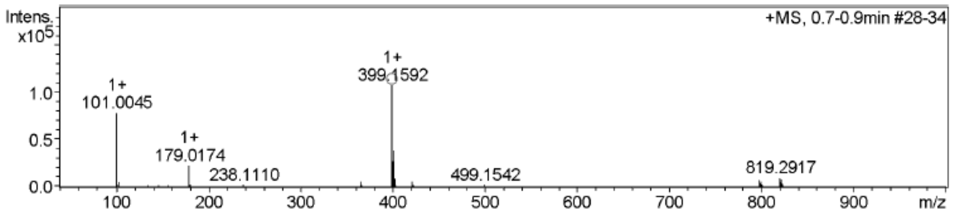


| # | m/z | I % |
|---|---|---|
| 1 | 101.0045 | 72.8 |
| 2 | 102.0069 | 2.5 |
| 3 | 103.0011 | 4.2 |
| 4 | 179.0174 | 20.9 |
| 5 | 238.1110 | 2.1 |
| 6 | 365.1964 | 5.2 |
| 7 | 399.1592 | 100.0 |
| 8 | 400.1616 | 24.9 |
| 9 | 401.1562 | 35.4 |
| 10 | 402.1592 | 8.2 |
| 11 | 421.1400 | 5.4 |
| 12 | 499.1542 | 2.6 |
| 13 | 797.3090 | 6.6 |
| 14 | 798.3108 | 3.4 |
| 15 | 799.3072 | 5.4 |
| 16 | 800.3106 | 2.3 |
| 17 | 819.2917 | 9.2 |
| 18 | 820.2941 | 4.8 |
| 19 | 821.2865 | 7.3 |
| 20 | 822.2841 | 3.1 |

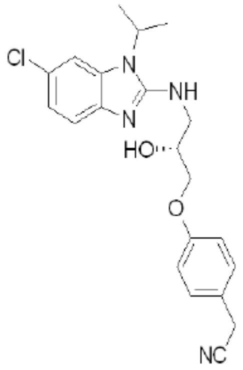


**Generate Molecular Formula Parameters**

| Charge | Tolerance | sigma limit | H/C Ratio | Electron Conf. | Nitrogen Rule | Chrom.BackGround | Calibration |
|---|---|---|---|---|---|---|---|
| +1 | 6 ppm | 0.08 | 3 - 0 | both | false | false | TRUE |

**Expected Formula** C21 H24 Cl1 N4 O2 **Adduct(s):** H, Na, NH4, C3H5N2, radical

| # | meas. m/z | theo. m/z | \|Err\|[ppm] | Sigma | Formula | Adduct | Adduct Mass |
|---|---|---|---|---|---|---|---|
| 1 | 399.1592 | 399.1582 | 2.40 | 0.0025 | C21H24ClN4O2 | M+ | -0.0005 |

Note: Sigma fits < 0.05 indicates high probability of correct MF

Figure S18. HRMS spectrum of **6w**.

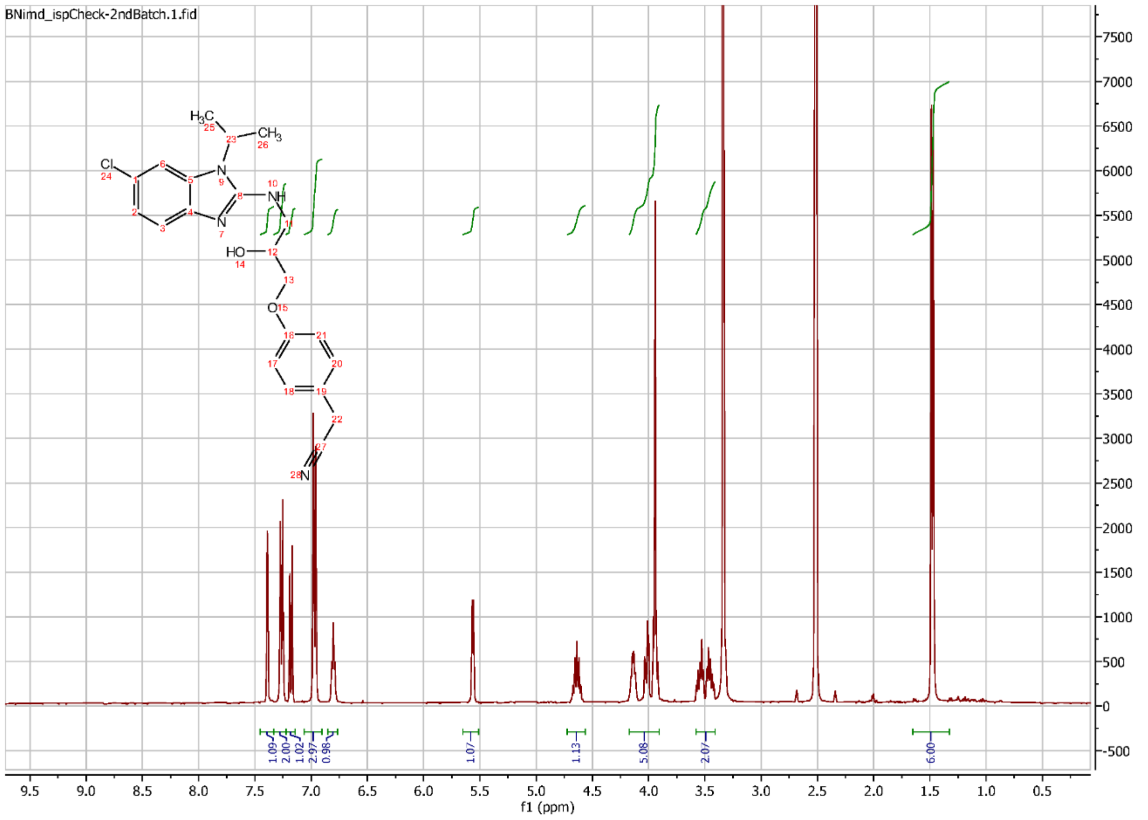

Figure S19. $^{1}$H-NMR spectrum of **6f**.

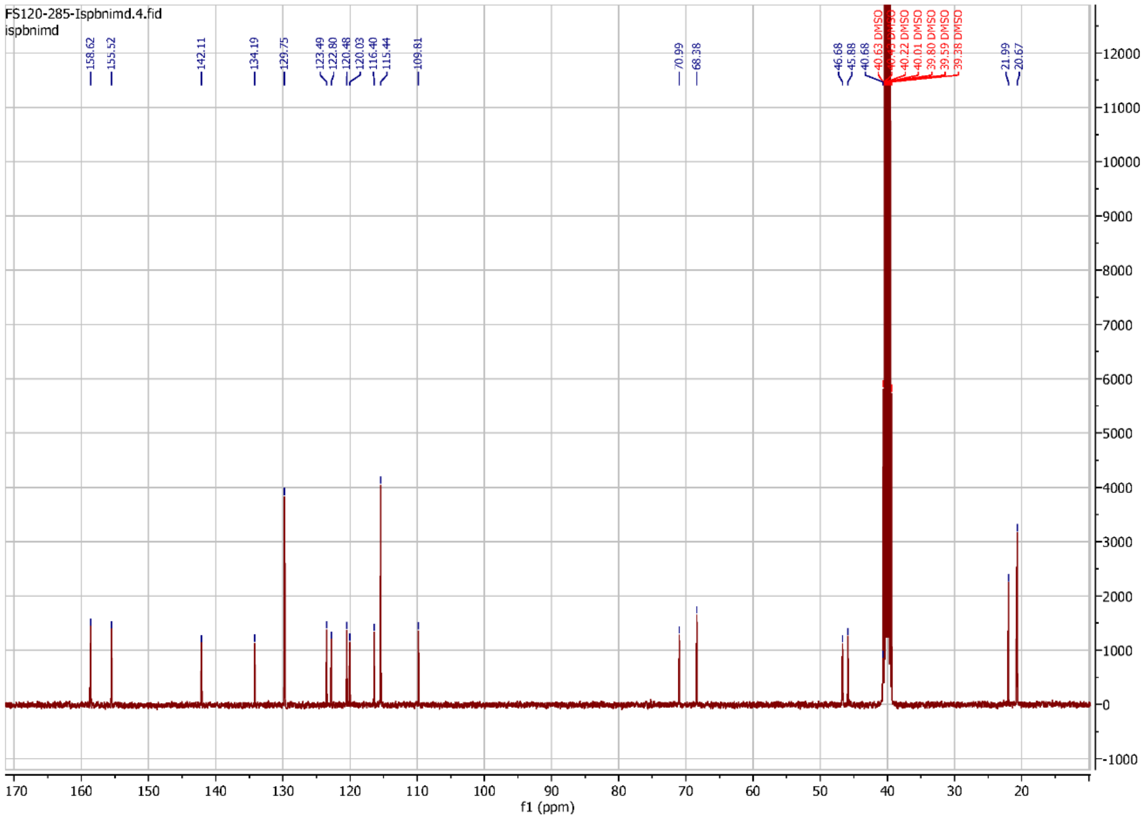

Figure S20. $^{13}$C-NMR spectrum of **6f**.

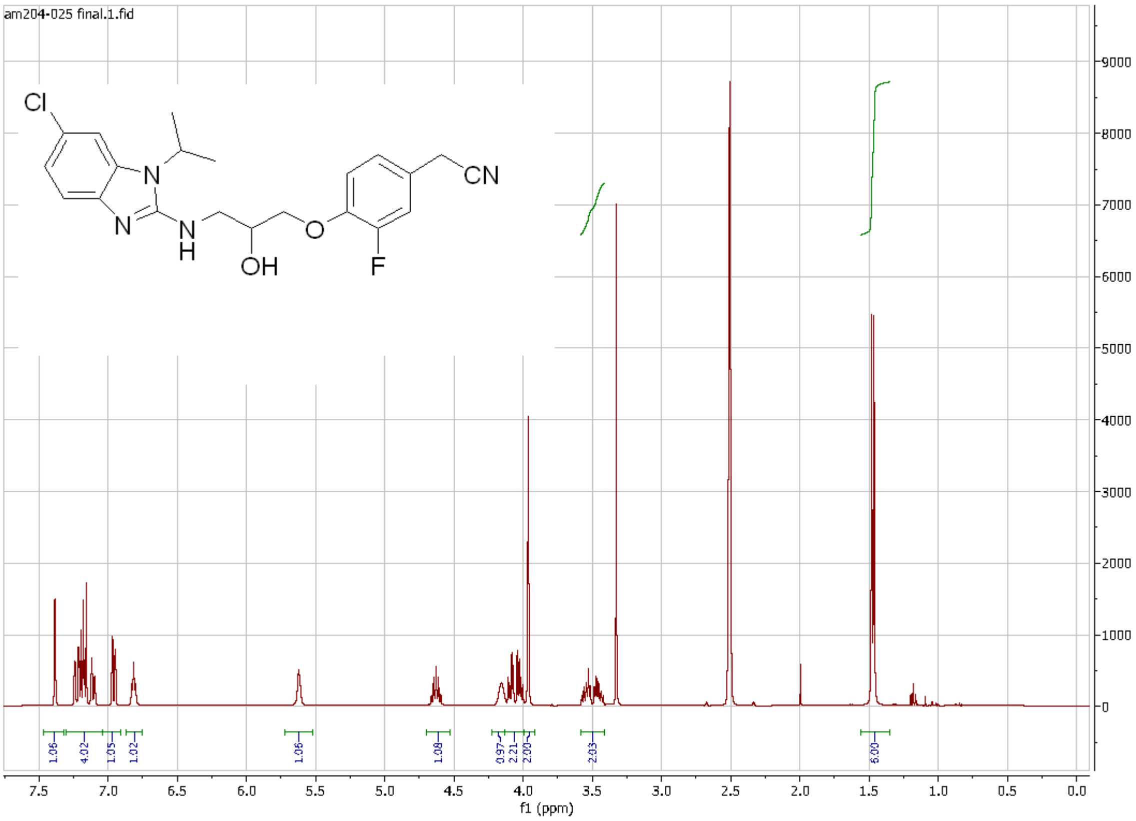


Figure S21. $^{1}$H-NMR spectrum of **6n**.

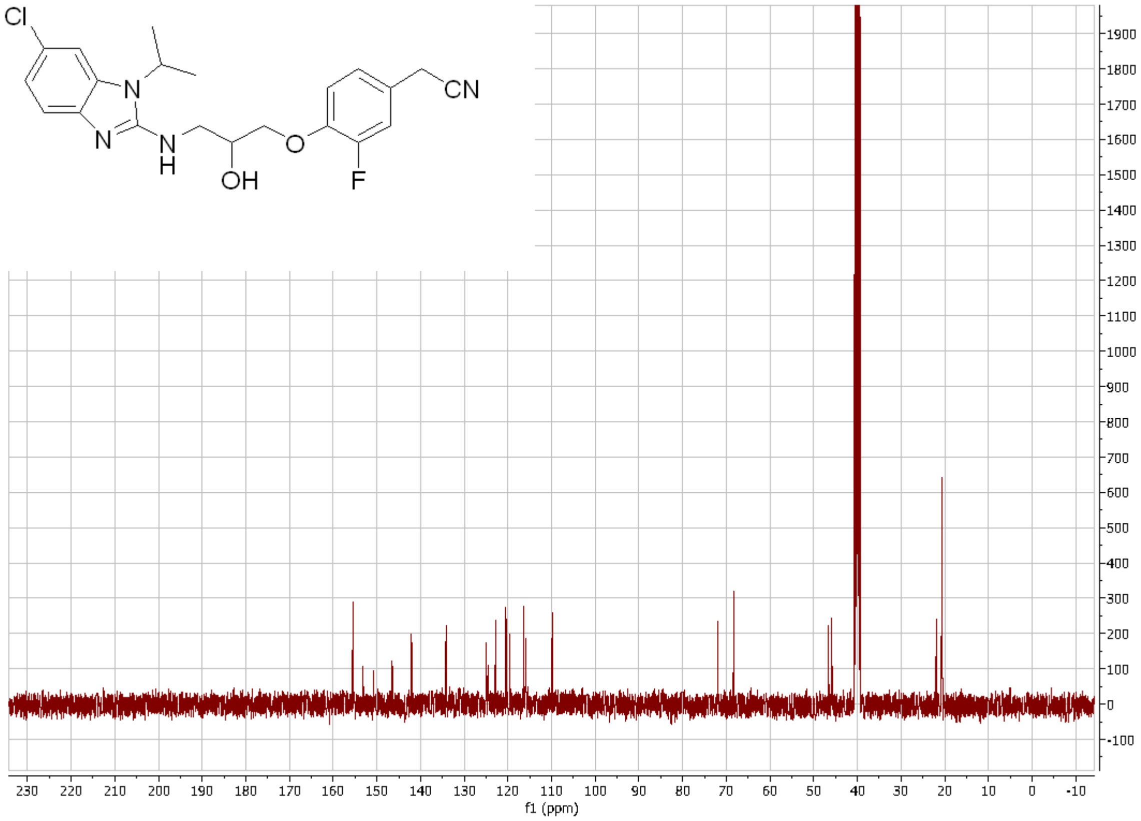


Figure S22. $^{13}$C-NMR spectrum of **6n**.

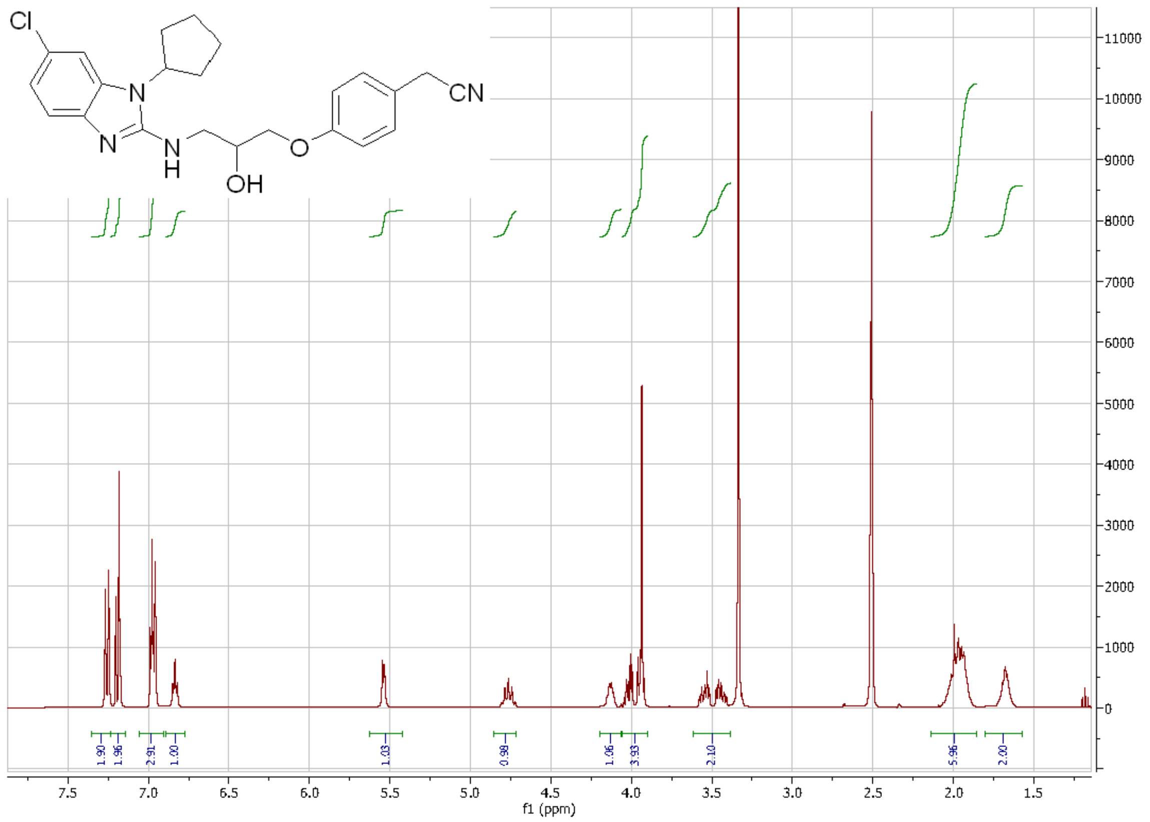

Figure S23. $^{1}$H-NMR spectrum of **6k.**

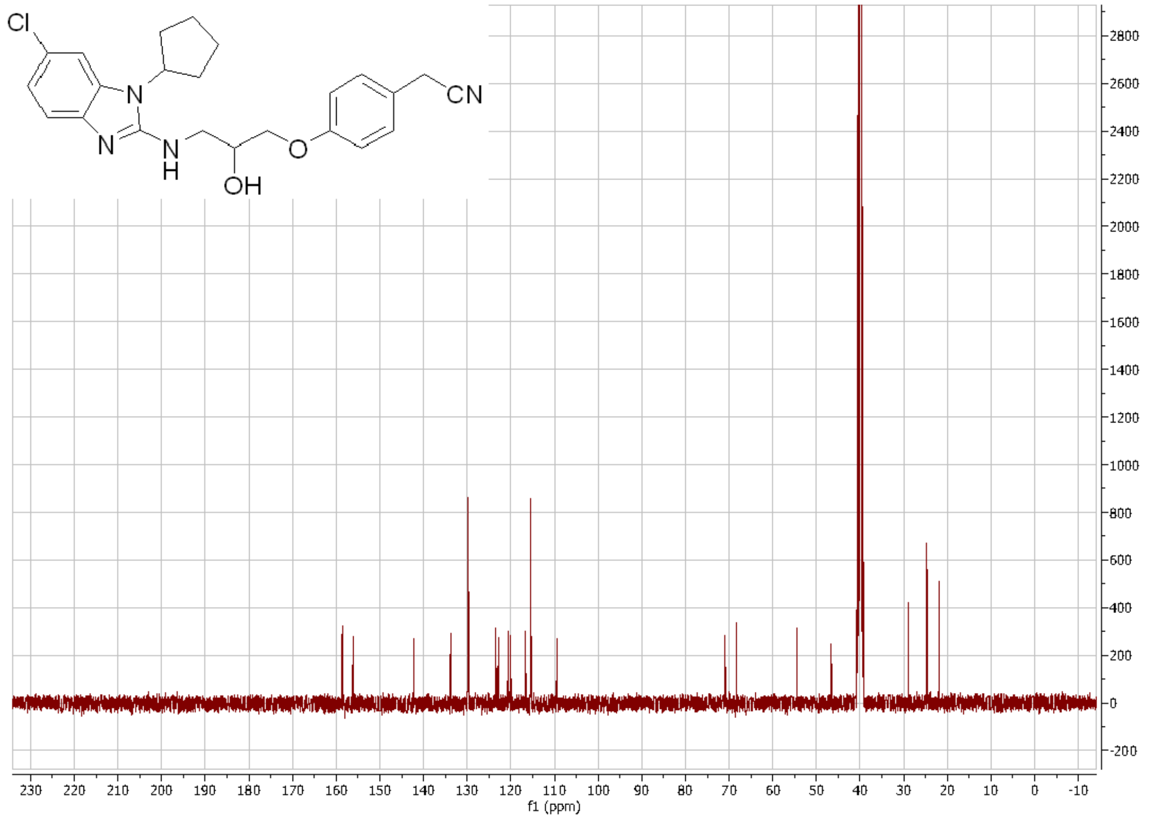

Figure S24. $^{13}$C-NMR spectrum of **6k.**

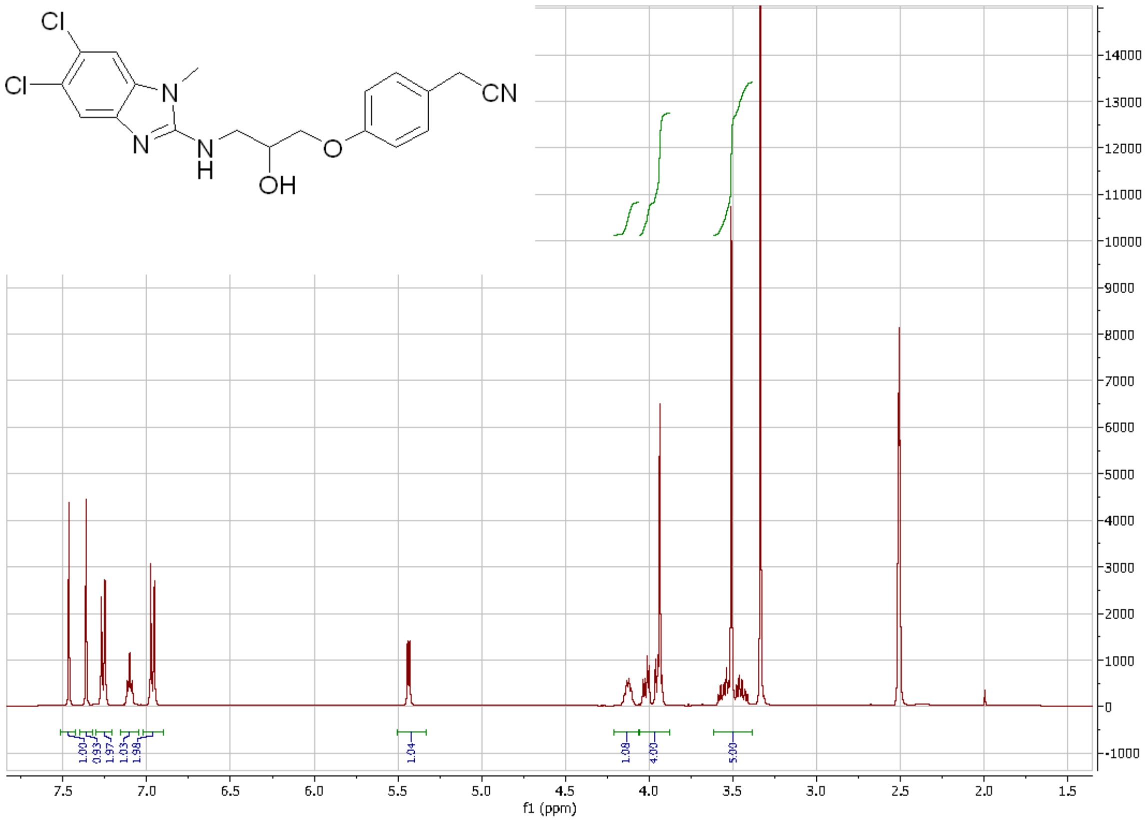


Figure S25. $^{1}$H-NMR spectrum of **6c.**

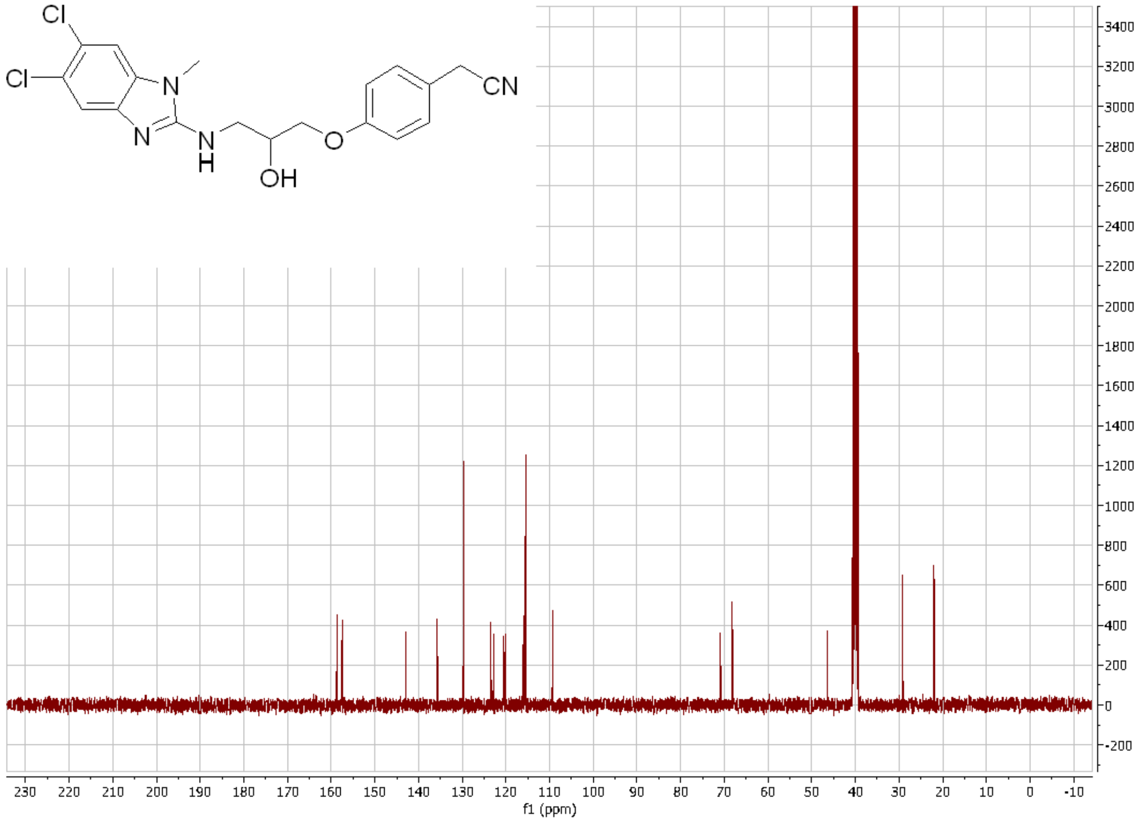


Figure S26. $^{13}$C-NMR spectrum of **6c**

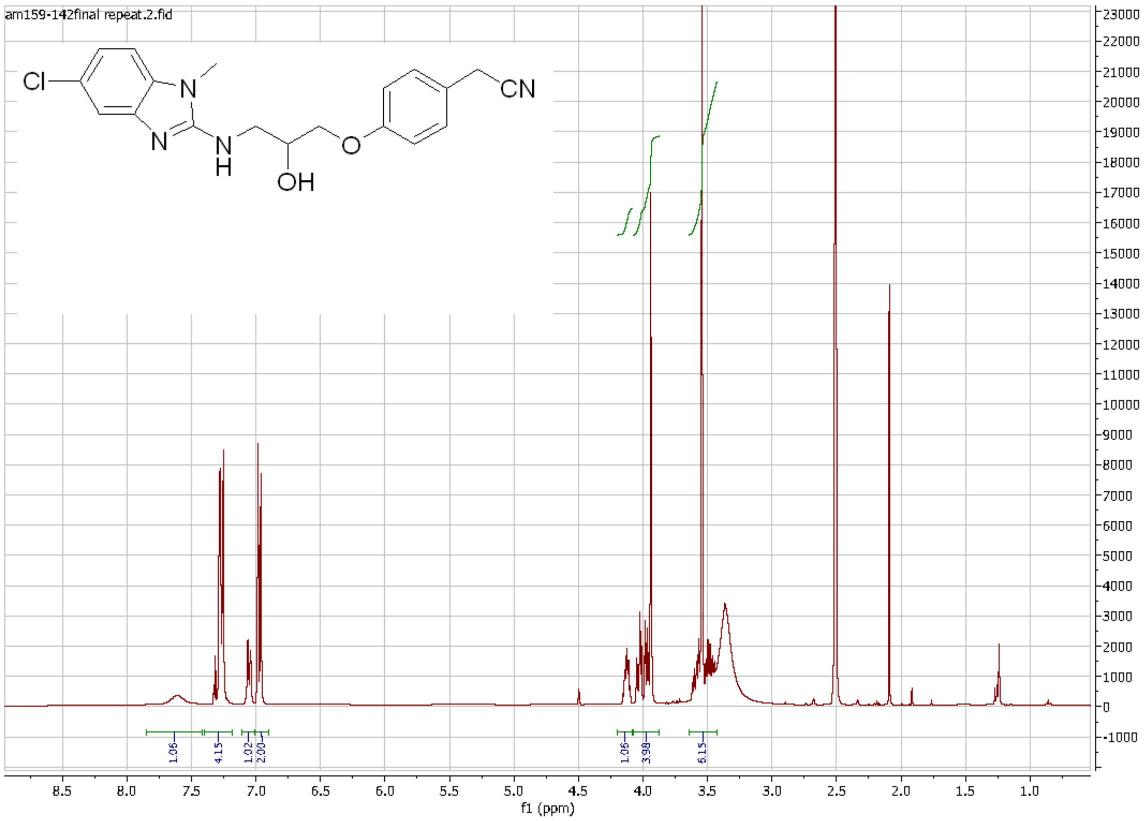

Figure S27. $^{1}$H-NMR spectrum of **6b**.

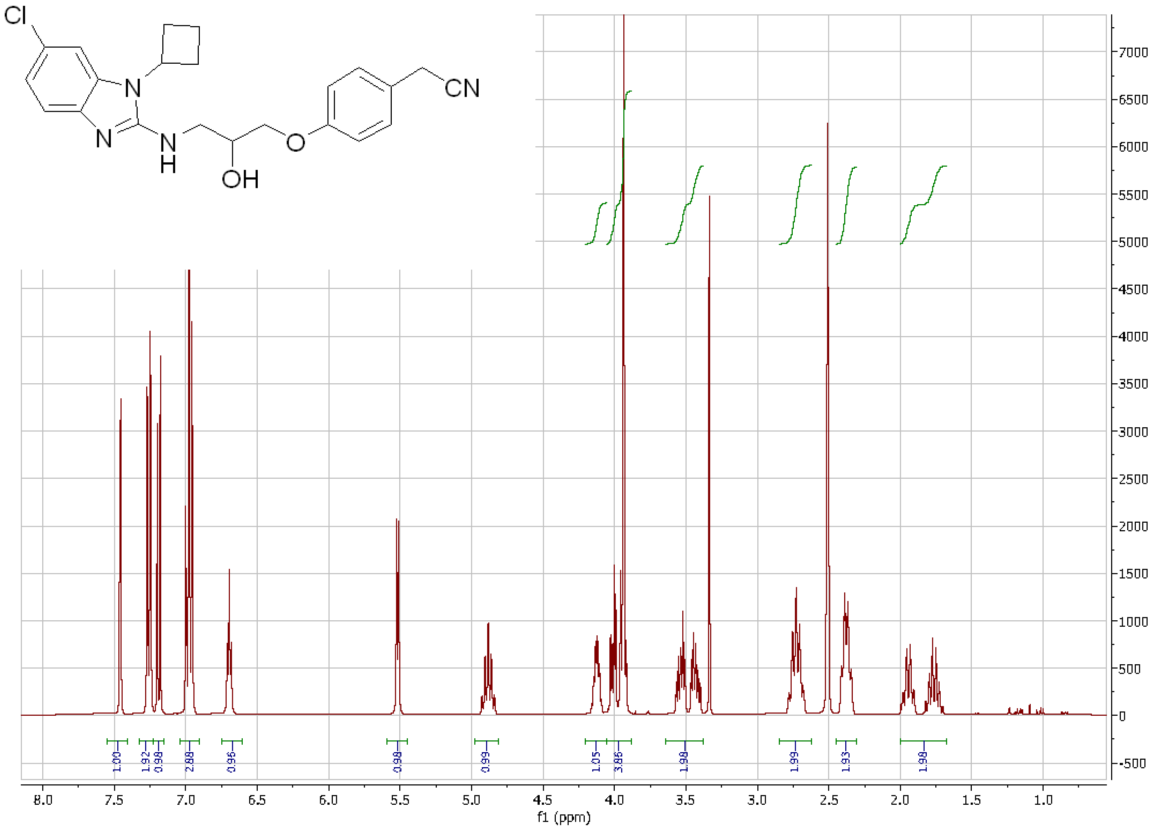

Figure S28. $^{1}$H-NMR spectrum of **6j**

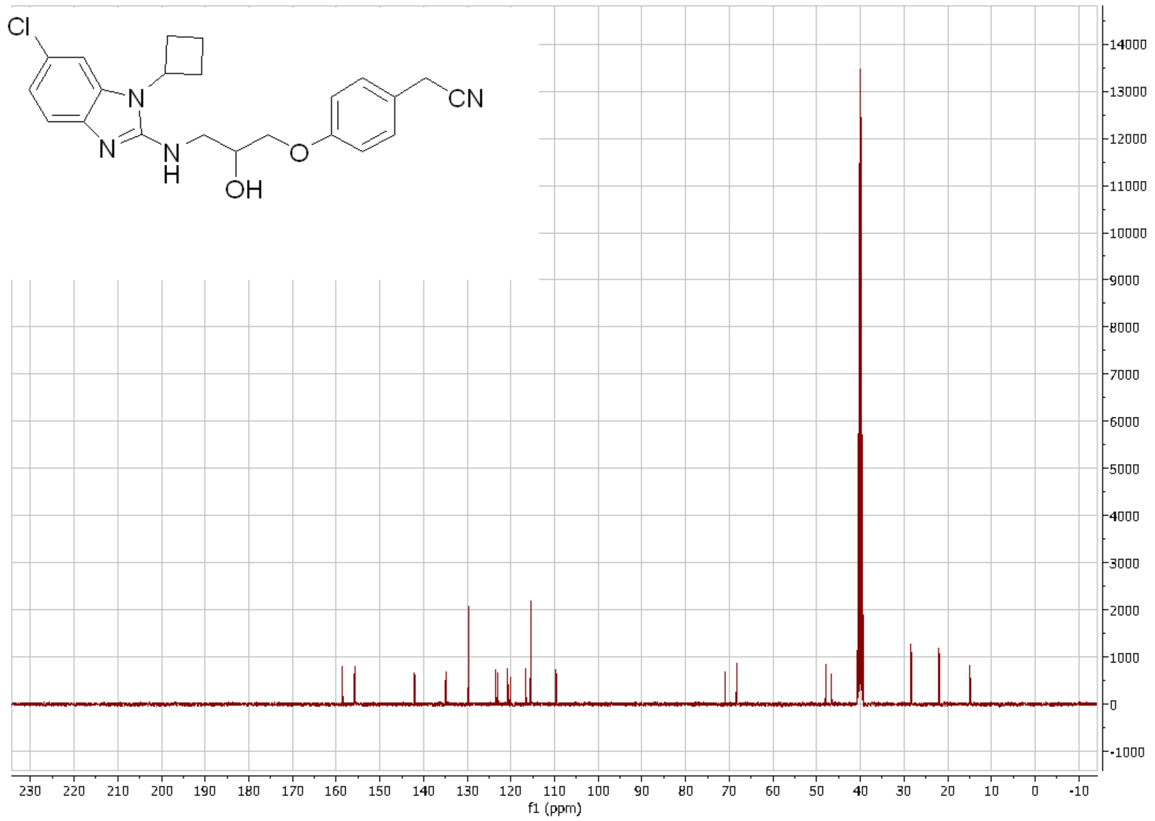


Figure S29. $^{13}$C-NMR spectrum of **6j**.

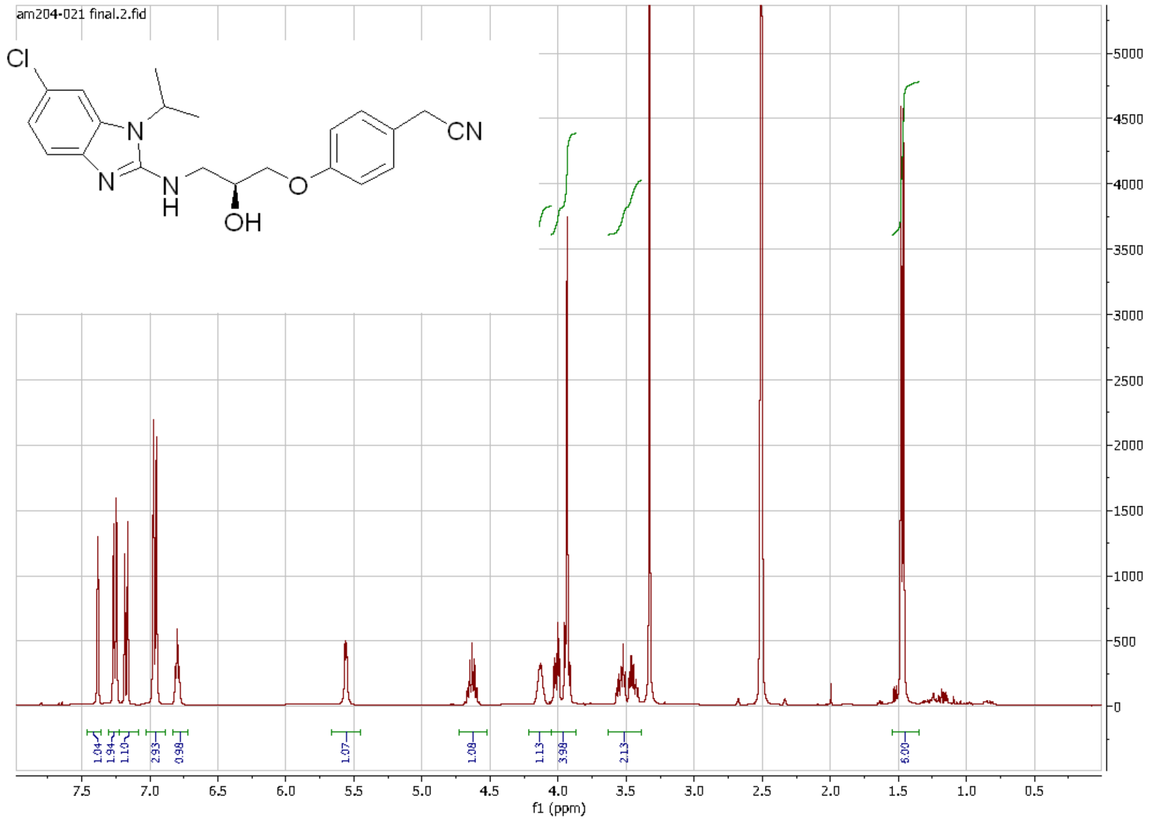


Figure S30. $^{1}$H-NMR spectrum of **6v**.

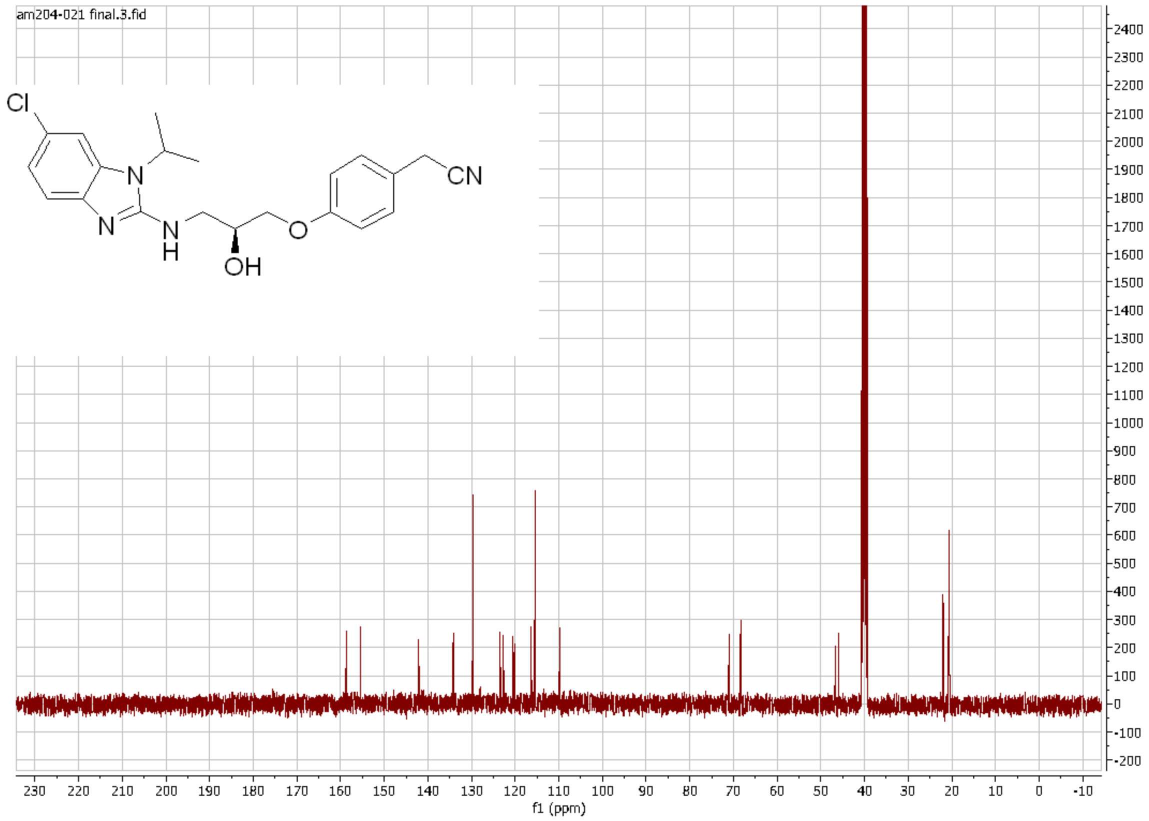


Figure S31. $^{13}$C-NMR spectrum of **6v**.

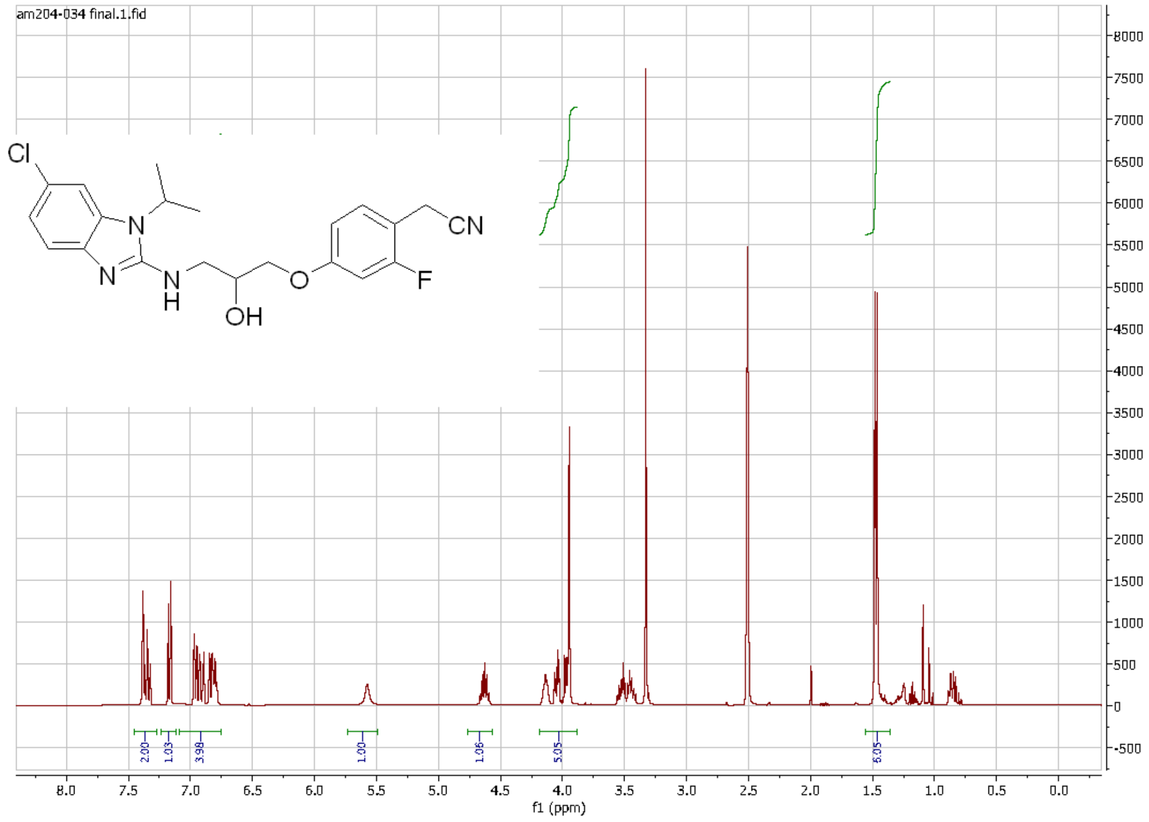


Figure S32. $^{13}$C-NMR spectrum of **6o**.

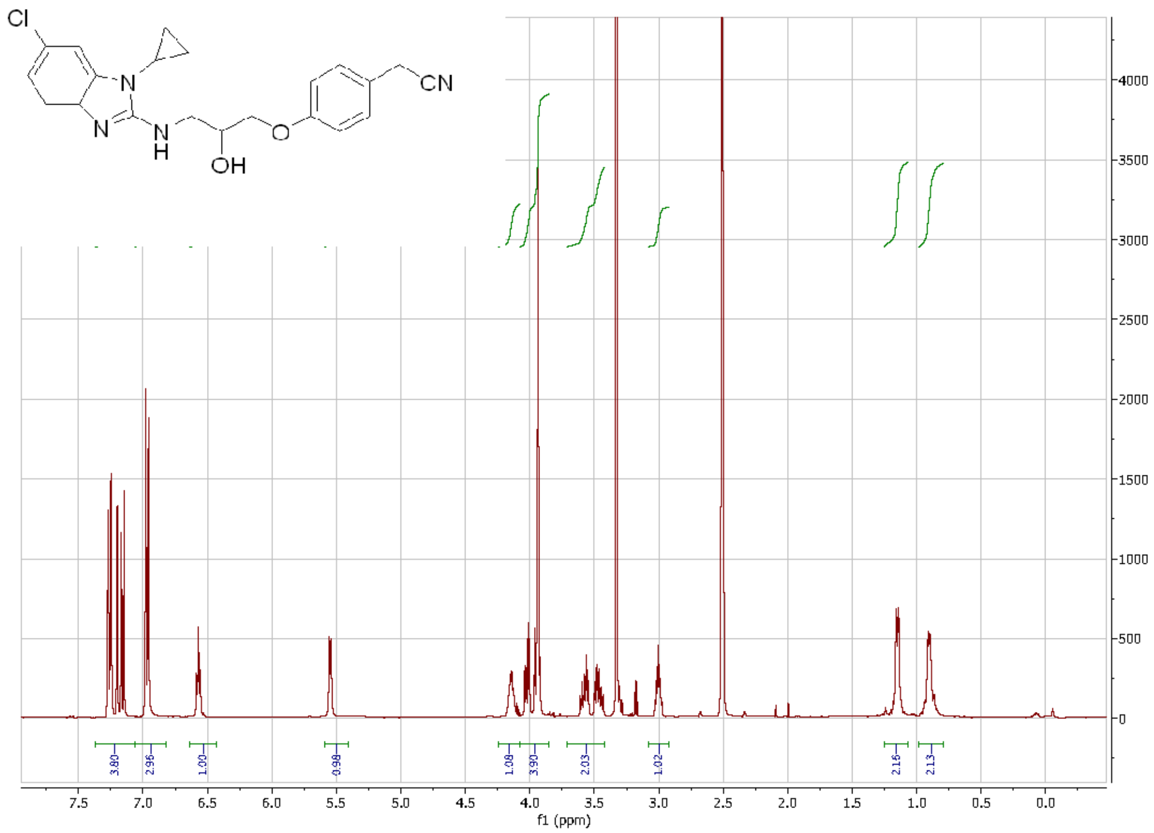


Figure S33. $^{1}$H-NMR spectrum of **6i**.

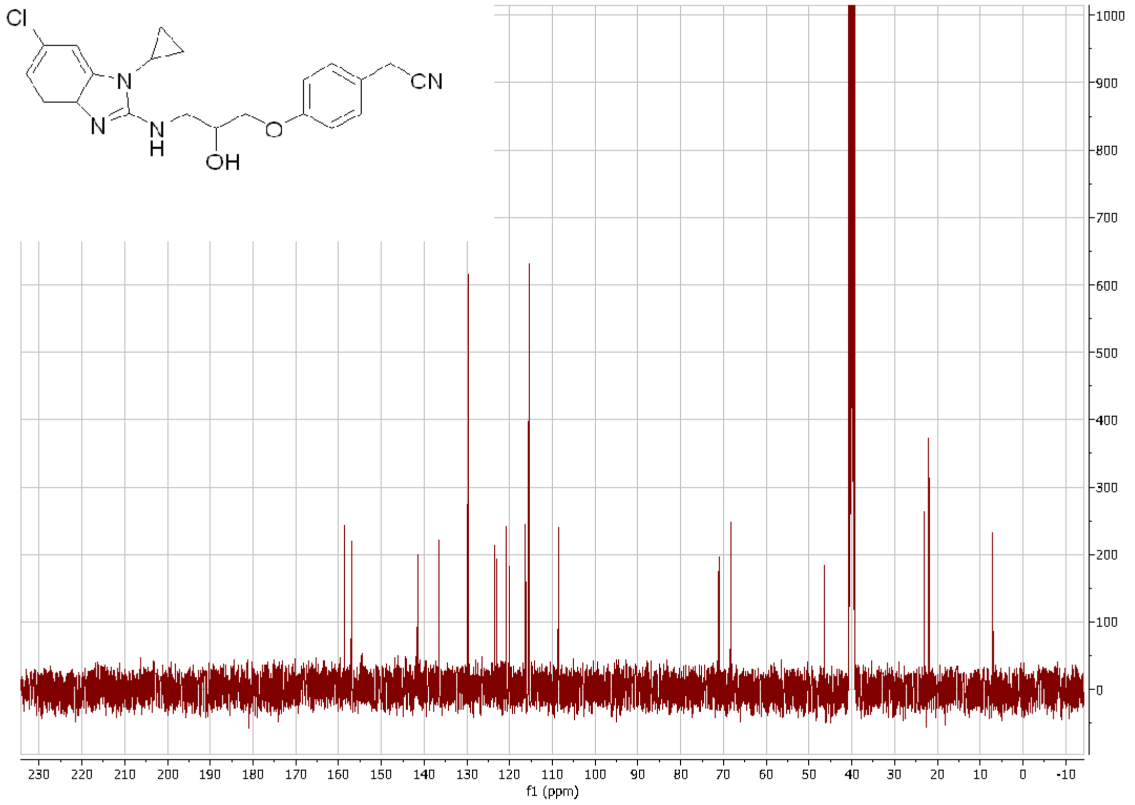

Figure S34. $^{13}$C-NMR spectrum of **6i**.

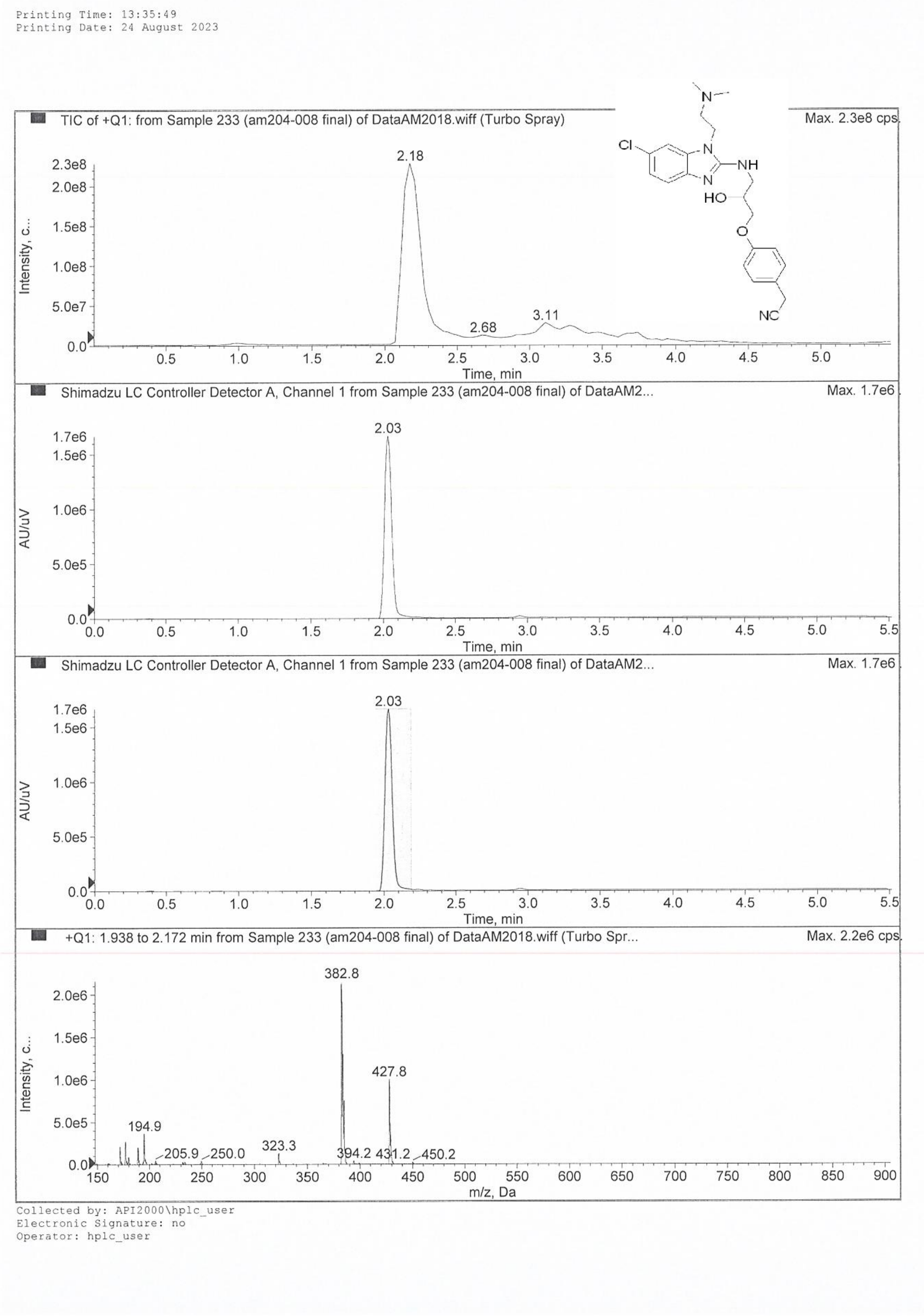


Figure S35. LCMS spectrum of **6q**.

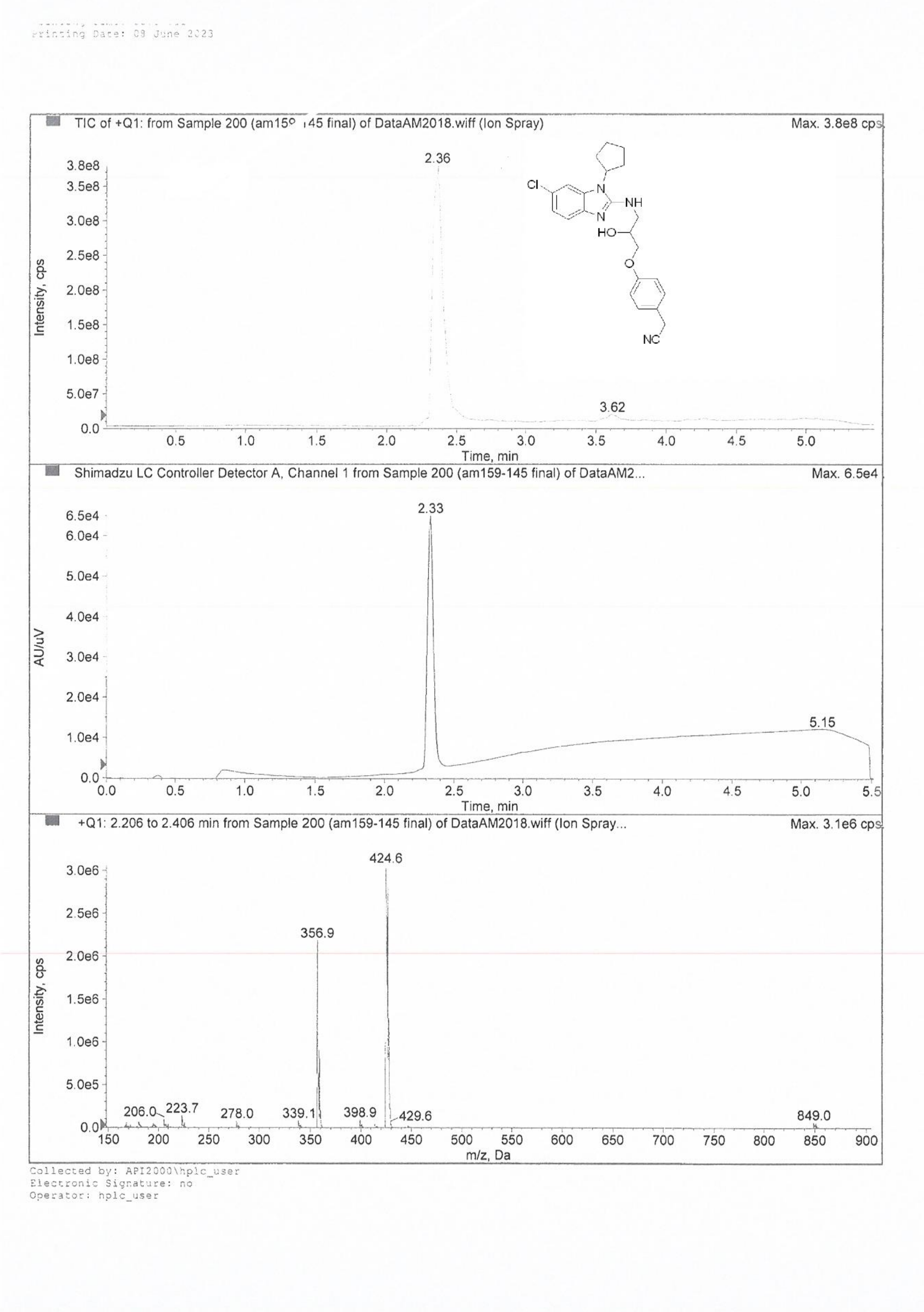


Figure S36. LCMS spectrum of **6k**.

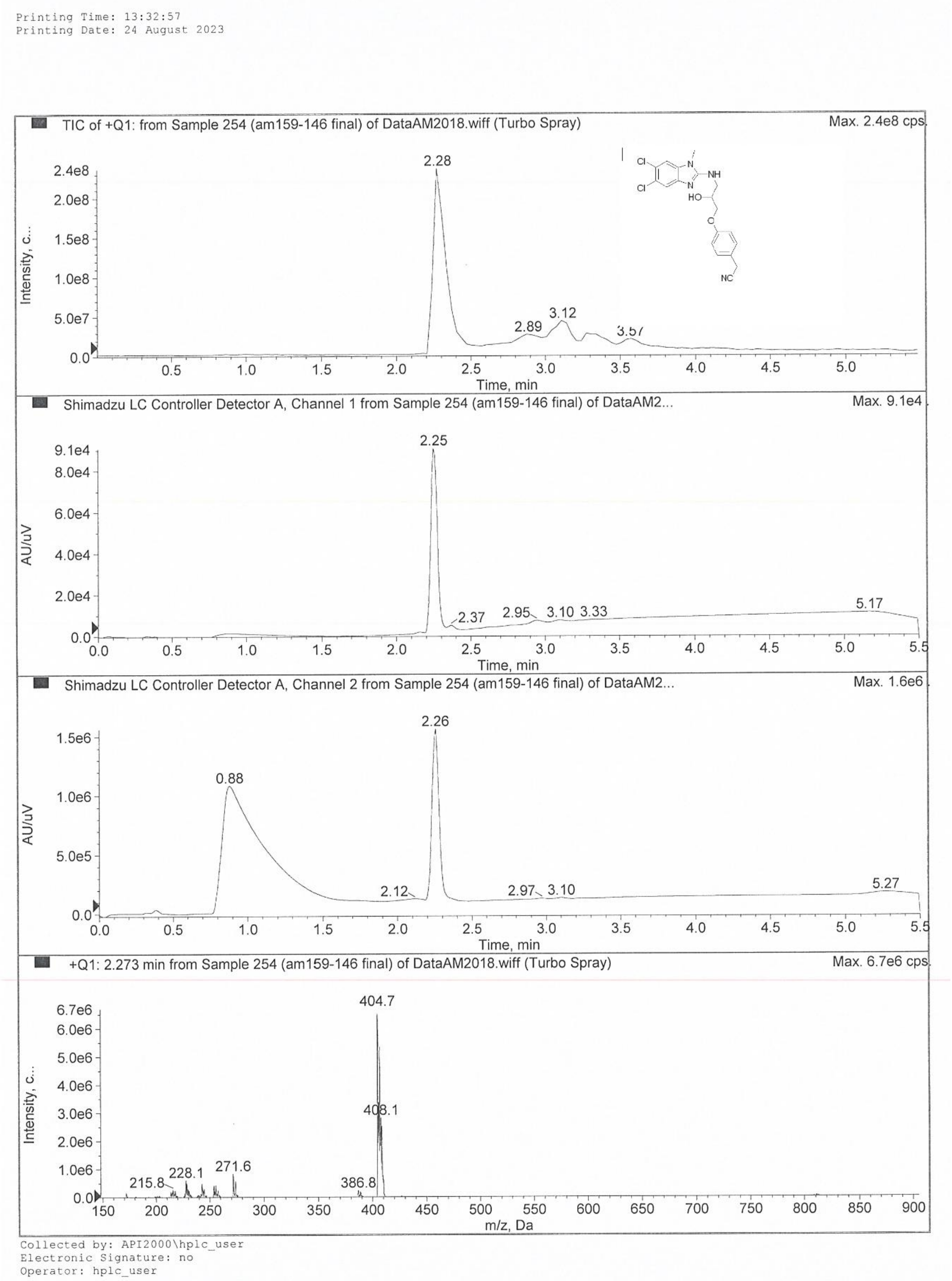


Figure S37. LCMS spectrum of **6c**.

Printing Time: 13:29:55
Printing Date: 24 August 2023

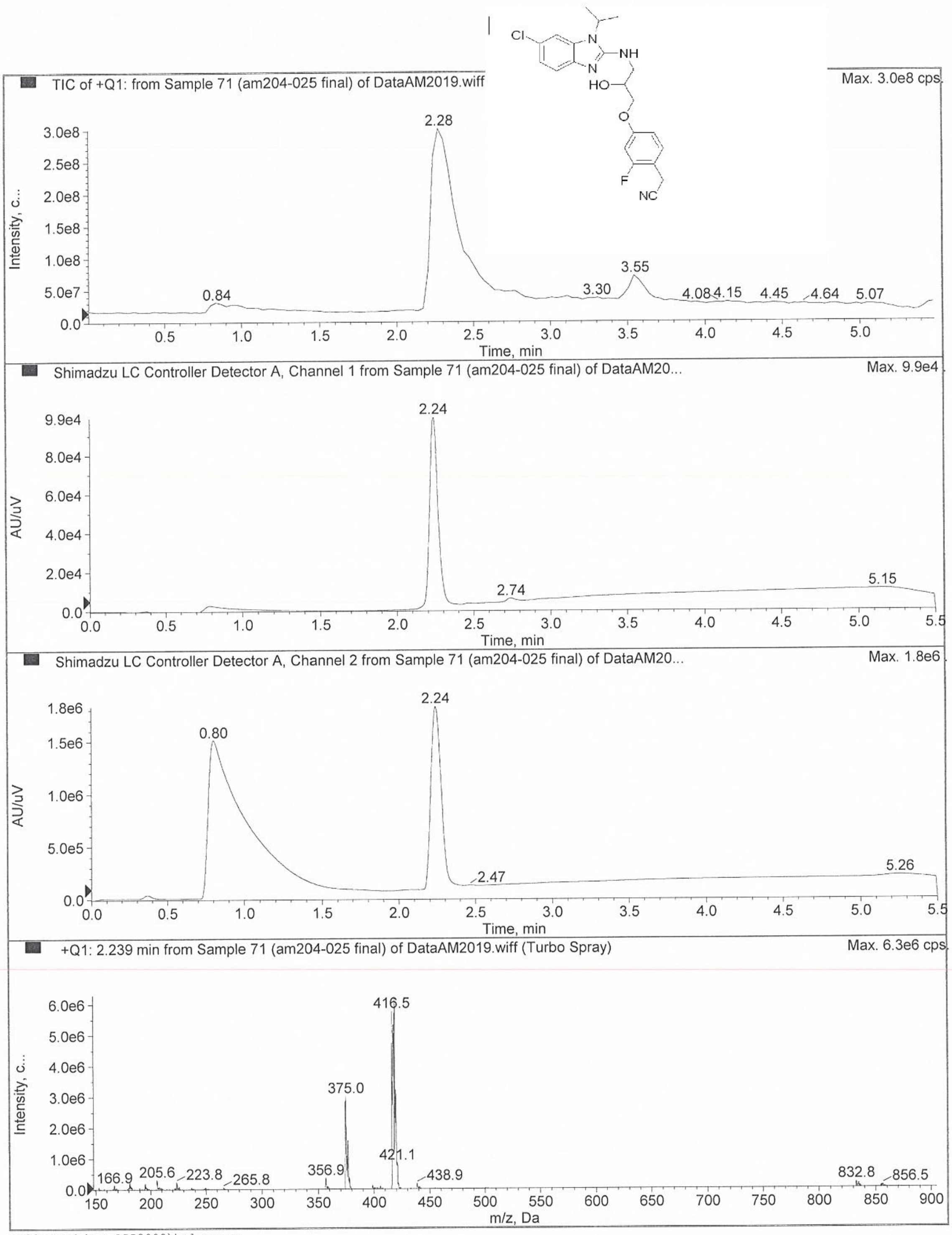


Collected by: API2000\hplc_user
Electronic Signature: no
Operator: hplc_user

Figure S38. LCMS spectrum of **6o**.

Printing Time: 13:27:34
Printing Date: 24 August 2023

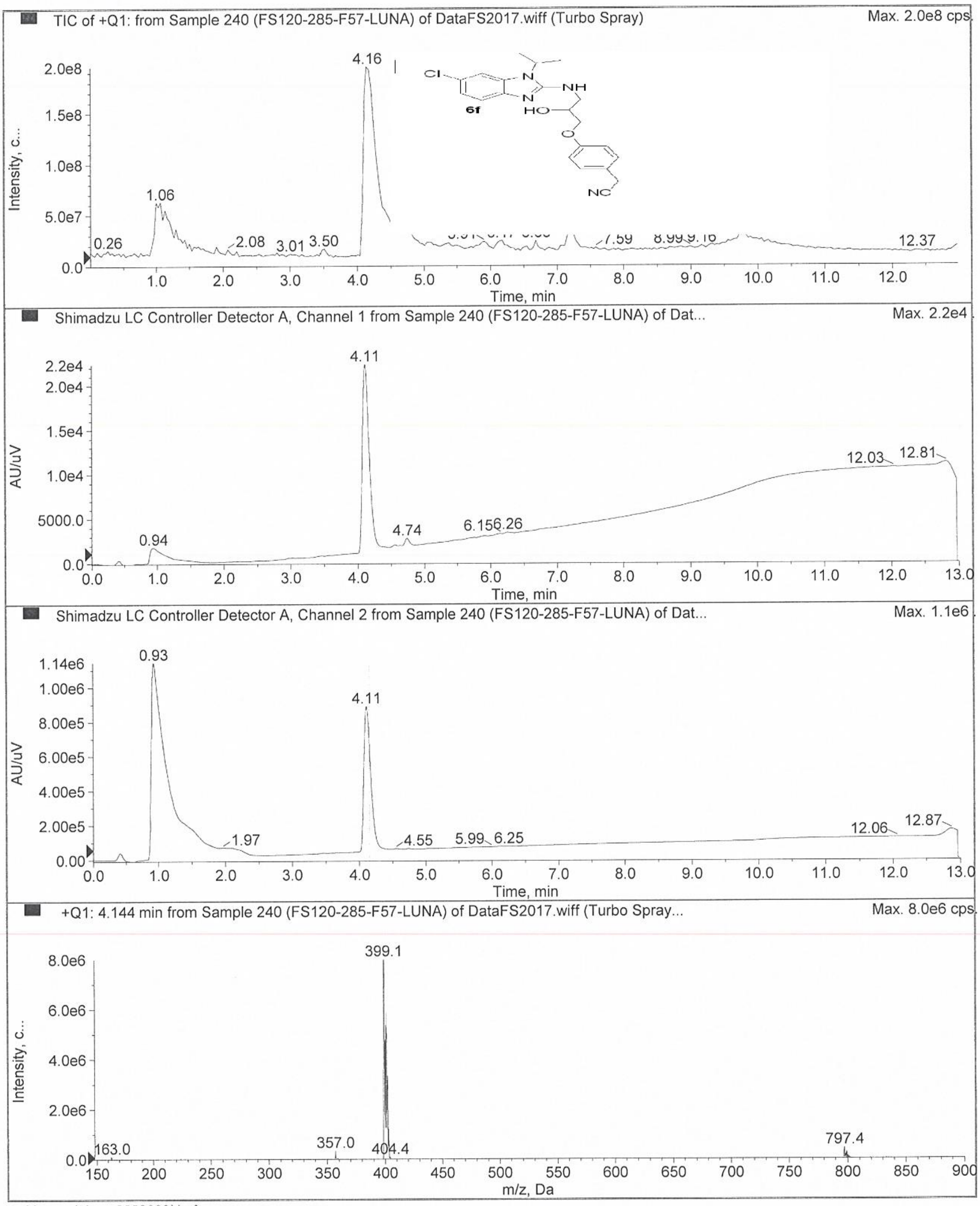


Collected by: API2000\hplc_user
Electronic Signature: no
Operator: hplc_user

Figure S39. LCMS spectrum of **6f**.

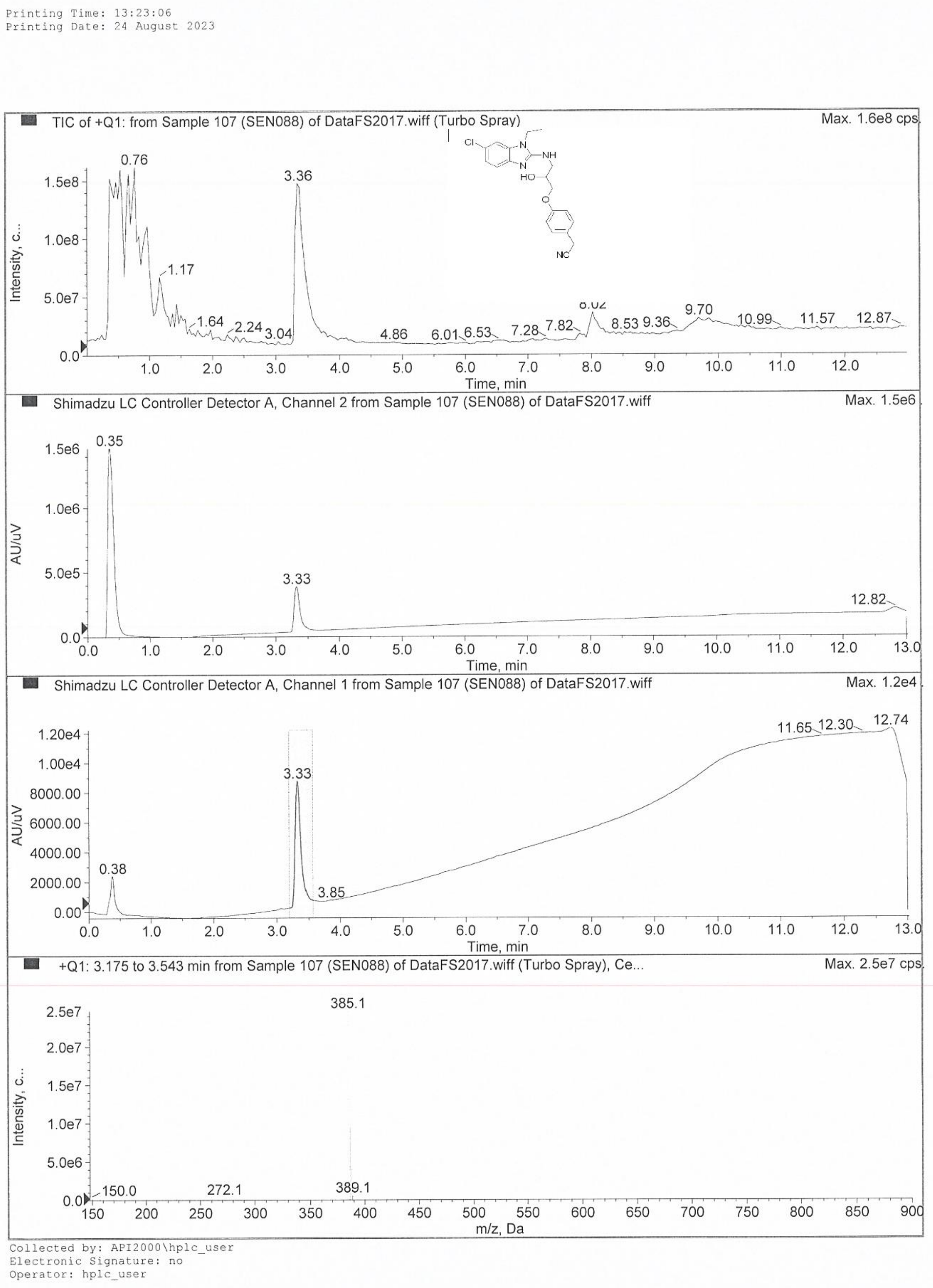


Figure S40. LCMS spectrum of **6e**.

Printing Time: 13:21:15
Printing Date: 24 August 2023

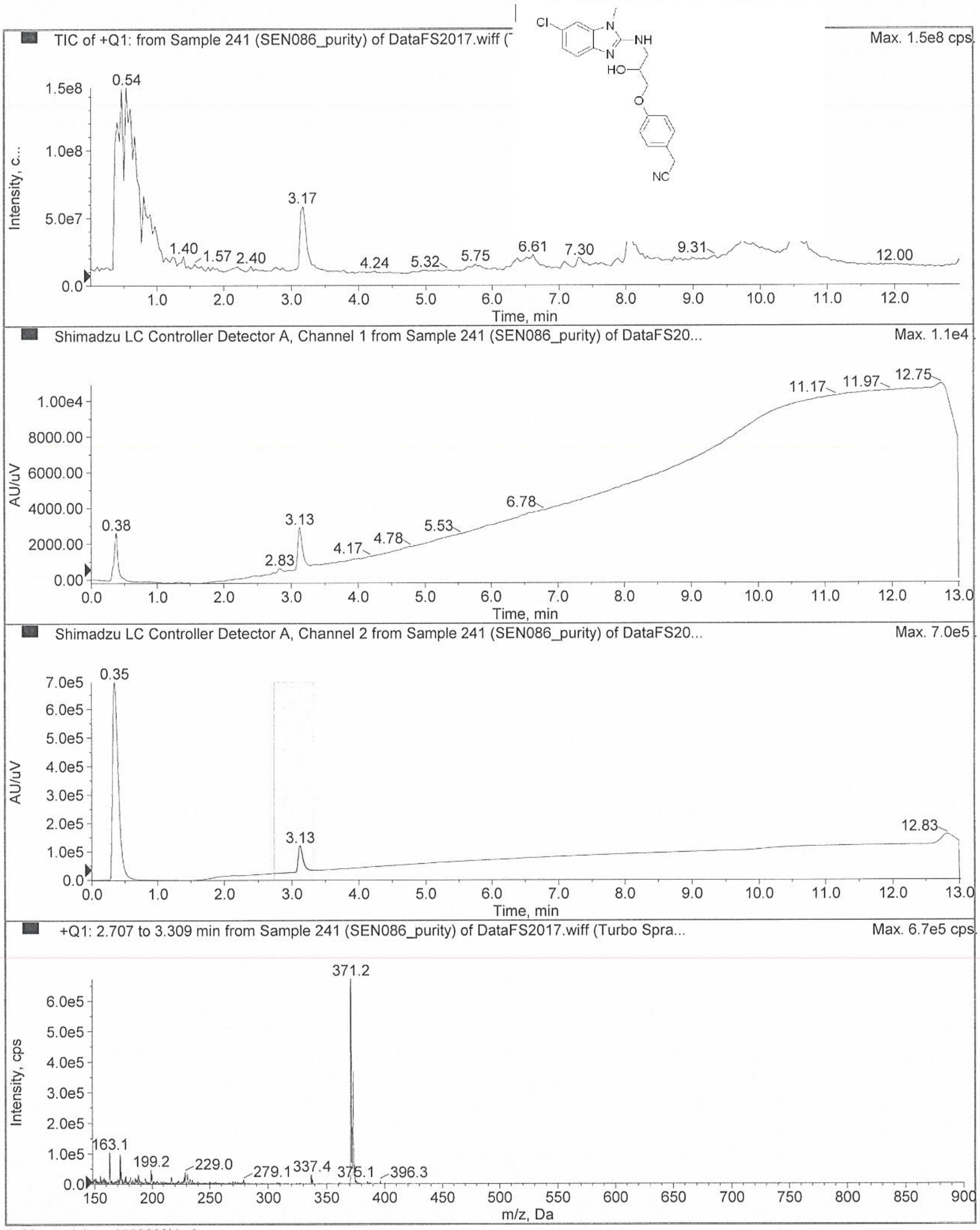


Collected by: API2000\hplc_user
Electronic Signature: no
Operator: hplc_user

Figure S41. LCMS spectrum of **6a**.

**References**


(1) Soukarieh, F.; Vico Oton, E.; Dubern, J. F.; Gomes, J.; Halliday, N.; de Pilar Crespo, M.; Ramirez-Prada, J.; Insuasty, B.; Abonia, R.; Quiroga, J.; et al. In Silico and in Vitro-Guided Identification of Inhibitors of Alkylquinolone-Dependent Quorum Sensing in *Pseudomonas aeruginosa*. *Molecules* **2018**, *23* (2). DOI: 10.3390/molecules23020257.
(2) Dubern, J.-F.; Romero, M.; Mai-Prochnow, A.; Messina, M.; Trampari, E.; Gijzel, H. N.-V.; Chan, K.-G.; Carabelli, A. M.; Barraud, N.; Lazenby, J.; et al. ToxR is a c-di-GMP binding protein that modulates surface-associated behaviour in *Pseudomonas aeruginosa*. *npj Biofilms and Microbiomes* **2022**, *8* (1). DOI: 10.1038/s41522-022-00325-9.
(3) Rahme, L. G.; Stevens, E. J.; Wolfort, S. F.; Shao, J.; Tompkins, R. G.; Ausubel, F. M. Common virulence factors for bacterial pathogenicity in plants and animals. *Science* **1995**, *268* (5219), 1899-1902. DOI: 10.1126/science.7604262.
(4) Lee, D. G.; Urbach, J. M.; Wu, G.; Liberati, N. T.; Feinbaum, R. L.; Miyata, S.; Diggins, L. T.; He, J.; Saucier, M.; Déziel, E.; et al. Genomic analysis reveals that *Pseudomonas aeruginosa* virulence is combinatorial. *Genome Biol* **2006**, *7* (10), R90. DOI: 10.1186/gb-2006-7-10-r90.
(5) Winstanley, C.; Langille, M. G. I.; Fothergill, J. L.; Kukavica-Ibrulj, I.; Paradis-Bleau, C.; Sanschagrin, F.; Thomson, N. R.; Winsor, G. L.; Quail, M. A.; Lennard, N.; et al. Newly introduced genomic prophage islands are critical determinants of in vivo competitiveness in the Liverpool Epidemic Strain of *Pseudomonas aeruginosa*. *Genome Res* **2009**, *19* (1), 12-23. DOI: 10.1101/gr.086082.108.
(6) Freschi, L.; Jeukens, J.; Kukavica-Ibrulj, I.; Boyle, B.; Dupont, M. J.; Laroche, J.; Larose, S.; Maaroufi, H.; Fothergill, J. L.; Moore, M.; et al. Clinical utilization of genomics data produced by the international *Pseudomonas aeruginosa* consortium. *Front Microbiol* **2015**, *6*, 1036. DOI: 10.3389/fmicb.2015.01036.
(7) Freschi, L.; Vincent, A. T.; Jeukens, J.; Emond-Rheault, J. G.; Kukavica-Ibrulj, I.; Dupont, M. J.; Charette, S. J.; Boyle, B.; Levesque, R. C. The *Pseudomonas aeruginosa* Pan-Genome Provides New Insights on Its Population Structure, Horizontal Gene Transfer, and Pathogenicity. *Genome Biol Evol* **2019**, *11* (1), 109-120. DOI: 10.1093/gbe/evy259.
(8) Roy, P. H.; Tetu, S. G.; Larouche, A.; Elbourne, L.; Tremblay, S.; Ren, Q.; Dodson, R.; Harkins, D.; Shay, R.; Watkins, K.; et al. Complete genome sequence of the multiresistant taxonomic outlier *Pseudomonas aeruginosa* PA7. *PLoS One* **2010**, *5* (1), e8842. DOI: 10.1371/journal.pone.0008842.
(9) Diggle, S. P.; Matthijs, S.; Wright, V. J.; Fletcher, M. P.; Chhabra, S. R.; Lamont, I. L.; Kong, X.; Hider, R. C.; Cornelis, P.; Cámara, M.; et al. The *Pseudomonas aeruginosa* 4-quinolone signal molecules HHQ and PQS play multifunctional roles in quorum sensing and iron entrapment. *Chem Biol* **2007**, *14* (1), 87-96. DOI: 10.1016/j.chembiol.2006.11.014.
(10) Popat, R.; Crusz, S. A.; Messina, M.; Williams, P.; West, S. A.; Diggle, S. P. Quorum-sensing and cheating in bacterial biofilms. *Proc Biol Sci* **2012**, *279* (1748), 4765-4771. DOI: 10.1098/rspb.2012.1976.
